# SERPINH1 overexpression in clear cell renal cell carcinoma: association with poor clinical outcome and its potential as a novel prognostic marker

**DOI:** 10.1111/jcmm.13495

**Published:** 2017-12-14

**Authors:** Yijun Qi, Yue Zhang, Zhiqiang Peng, Lei Wang, Kaizhen Wang, Duiping Feng, Junqi He, Junfang Zheng

**Affiliations:** ^1^ Department of Biochemistry and Molecular Biology Capital Medical University Beijing China; ^2^ Department of Urology Beijing Friendship Hospital Capital Medical University Beijing China; ^3^ Department of Radiology First Hospital of Shanxi Medical University Taiyuan China; ^4^ Beijing Key Laboratory for Cancer Invasion and Metastasis Research Beijing International Cooperation Base for Science and Technology on China‐UK Cancer Research Beijing China

**Keywords:** renal cancer, proteomics, transcriptomics, prognostic marker, SERPINH1/HSP47

## Abstract

Precision therapy for clear cell renal cell carcinoma (ccRCC) requires molecular biomarkers ascertaining disease prognosis. In this study, we performed integrated proteomic and transcriptomic screening in all four tumour‐node‐metastasis stages of ccRCC and adjacent normal tissues (*n *=* *18) to investigate differentially expressed genes. Most identified differentially expressed genes revealed a strong association with transforming growth factor‐β level and the epithelial‐to‐mesenchymal transition process. Of them, Serpin peptidase inhibitor clade H member 1 (SERPINH1) revealed the strongest association with poor prognosis and regulation on the expression levels of epithelial‐to‐mesenchymal transition markers. Subsequently, two independent sets (*n *=* *532 and 105) verified the high level of SERPINH1 in ccRCC tissues and its association with reduced overall survival and disease‐free survival in all tumour‐node‐metastasis stages and patients with *von Hippel–Lindau* wild‐type (*VHL*‐WT). SERPINH1 was an independent predictor of poor overall survival (hazard ratio 0.696 for all patients) and disease‐free survival (hazard ratio 0.433 for all patients and 0.362 for patients with *VHL‐* WT) in ccRCC. We have thus shown for the first time that SERPINH1 is an independent precision predictor for unfavourable prognosis in ccRCC. This could assist in identifying patients who need early aggressive management and deepen our understanding of the pathogenesis of *VHL*‐WT ccRCC.

## Introduction

Renal cell carcinoma (RCC) accounts for about 90% of all kidney tumours. Its incidence is increasing and outcome remains poor. Clear cell RCC (ccRCC) is the most common adult renal neoplasm. The outcome of ccRCC patients greatly varies after nephrectomy. Hence, accurate discrimination of ccRCC patients with poor prognosis is very important for appropriate and effective management. However, prognosis is currently assessed based on histological parameters. Neither of histological parameters is sufficiently accurate for risk discrimination 1. ccRCC originates from the dysregulated expression of different genes. Dysregulated expression of these molecules might predict the prognosis of patients. Therefore, molecular analyses hold the promise of accurately predicting disease outcome in ccRCC 2, and more effective precision molecular markers need to be identified.

Transforming growth factor‐β (TGFβ)‐induced epithelial‐to‐mesenchymal transition (EMT) is an important process in ccRCC occurrence and progression 3, 4. Recently, an increasing number of EMT‐related genes have been reported to correlate with ccRCC development and act as ccRCC prognostic markers 5. EMT can be regulated by von Hippel–Lindau (VHL), a notable tumour suppressor in ccRCC. The *VHL* gene mutation is the predominant cause of *VHL* inactivation 6. However, whether *VHL* mutation would affect the precise prognostic judgement and therapy of ccRCC patients in specific condition remains unknown.

Serpin peptidase inhibitor clade H member 1 (SERPINH1, also called HSP47) belongs to the serpin superfamily and has a signal sequence at the N‐terminus, two N‐glycosylation sites and an ER retention signal (Arg‐Asp‐Glu‐Leu, RDEL) at the C‐terminus 7. It was originally thought to be a collagen‐binding stress protein on the cell surface, which was later identified as an endoplasmic reticulum‐resident protein with collagen‐binding properties. The unique properties of SERPINH1 in modulating collagen production and its location on the cell membrane in many forms of cancer have led SERPINH1 to be designated as a potential biomarker or therapeutic target for a number of conditions and diseases 8.

In this study, for the first time, we demonstrate that the high level of SERPINH1 has the strongest association with poor prognosis of ccRCC patients among our EMT‐related differentially expressed genes (DEGs). The association of SERPINH1/HSP47 level with poor outcome was verified in two independent cohorts, and its regulation on the expression of EMT markers was confirmed in ccRCC cells. Importantly, we observed that SERPINH1 was a potential independent prognostic marker, particularly in patients with *VHL* wild‐type (*VHL*‐WT) ccRCC. These results contribute to improving the ccRCC patient prognosis prediction system and deepening the understanding of the pathogenesis of *VHL*‐WT ccRCC.

## Materials and methods

### Tissue collection and study design

We obtained 33 pairs of primary ccRCC and adjacent normal kidney tissues (Table 1, 18 pairs for discovery and 15 pairs for validation) from nephrectomies conducted at the Beijing Friendship Hospital between 2013 and 2014. These tissues were immediately frozen in liquid nitrogen and stored at −80°C. We also obtained 90 pairs of samples (81 had follow‐up data) collected from 2006 to 2008 (Table 1, detailed information was in Zheng *et al*., 2017) as validation cohort and for prognosis analyses. The research was approved by the Research Ethics Board of Beijing Friendship Hospital and was carried out according to the World Medical Association Declaration of Helsinki. All patients included in the protocol signed a declaration of informed consent.

**Table 1:** Summary of clinicopathological features of ccRCC patients

Characteristics	Discovery set (18 pairs of ccRCC and adjacent normal tissue for iTRAQ analysis and transcriptomic analysis)	Validation set (532 ccRCC and 72 adjacent normal tissue in TCGA_KIRC data set for mRNA level, OS and DFS analyses)	Validation set (15 pairs of ccRCC and adjacent normal tissue for WB analysis)	Validation set (90 pairs of ccRCC and adjacent normal tissue for TMA construction and paired IHC and OS analyses)
Age (year)				
≤60	7 (38.9%)	255 (47.9%)	5 (33.3%)	49 (54.4%)
>60	11 (61.1%)	260 (48.9%)	10 (66.7%)	41 (45.6%)
Unknown	0 (0%)	17 (3.2%)	0 (0%)	0 (0%)
Sex				
Male	18 (100%)	334 (62.8%)	10 (66.7%)	51 (56.7%)
Female	0 (0%)	181 (34%)	5 (33.3%)	39 (43.3%)
Unknown	0 (0%)	17 (3.2%)	0 (0%)	0 (0%)
Pathological grade				
G1	2 (11**.**1%)	13 (2.5%)	2 (13.3%)	33 (36**.**7%)
G2	14 (77.8%)	229 (43.0%)	13 (86.7%)	42 (46.7%)
G3	2 (11.1%)	205 (38.5%)	0 (0%)	14 (15.5%)
G4	0 (0%)	77 (14.5%)	0 (0%)	1 (1**.**1%)
Unknown	0 (0%)	8 (1.5%)	0 (0%)	0 (0%)
AJCC TNM stage				
I	8 (44**.**4%)	255 (47.9%)	12 (80%)	60 (66**.**7%)
II	7 (38**.**9%)	56 (10.5%)	3 (20%)	18 (20%)
III	1 (5**.**6%)	127 (23.9%)	0 (0%)	4 (4**.**4%)
IV	2 (11**.**1%)	81 (15.2%)	0 (0%)	2 (2**.**2%)
Unknown	0 (0%)	13 (2.5%)	0 (0%)	6 (6**.**7%)

In addition, mRNA expression data (RNA Seq v2) and clinical information for patients in The Cancer Genome Atlas_kidney renal clear cell carcinoma (TCGA_KIRC, 532 cases, Table 1) data set were downloaded from https://www.synapse.org and cBioPortal database (www.cbioportal.org), respectively, and used for differential mRNA expression and prognosis analyses.

### Isobaric tags for relative and absolute quantitation (iTRAQ)‐based proteomic analysis

Tissue protein levels were obtained from our previously published proteomics study. The raw data and statistics for protein levels in tumours compared with adjacent normal tissues were also from the published paper 9.

### Transcriptomic analysis

To identify DEGs between ccRCC and paired adjacent normal tissues, six groups (tumour groups C1‐C3, normal groups N1‐N3) were divided according to stage and tissue types. Equal mRNAs in stage I, II and III, respectively, were analysed by mRNA microarray as reported 10. Human Whole Genome OneArray^®^ v6.1 (Phalanx Biotech Group, Hsinchu, Taiwan) was used. The gene expression data have been deposited in the National Center for Biotechnology (NCBI) Gene Expression Omnibus database (http://www.ncbi.nlm.nih.gov/geo/) and are accessible through GEO Series accession number GSE100666.

### Gene set enrichment analysis

The association between clinical classification [good (≥5 years, living) and poor (≤2 years, die) prognosis] and expression levels of genes was analysed using Gene Set Enrichment Analysis (GSEA v2.2, http://www.broad.mit.edu/gsea) as reported 9. A gene set is considered significantly enriched when the false discovery rate (FDR) score is <0.25.

### Western blotting and immunohistochemistry

Western blotting (WB) and immunohistochemistry (IHC) were performed as described 9. For WB, anti‐SERPINH1 and anti‐β‐actin antibodies (1:1000) were purchased from Sigma‐Aldrich (St. Louis, MO, USA). HRP‐conjugated secondary antibody was from Amersham Biosciences (Little Chalfont, UK). The blots were quantified using NIH Image 1.62 program. The protein level was normalized with β‐actin. For IHC, sections were incubated with anti‐SERPINH1 antibody (1:100). Image‐Pro plus 6.0 (MediaCybernetics Inc., SilverSpring, MD, USA) was used to analyse optical densitometry.

### Statistics

The results of paired and unpaired samples were analysed by paired sample and independent sample *t*‐test, respectively. Univariate and multivariate Cox proportional hazard regression analyses were used to estimate the prognostic significance of SERPINH1 in ccRCC. The effect of SERPINH1 expression level on the constituent ratio of good/poor prognosis was explored by Pearson chi‐square test. The log‐rank test for the generated Kaplan–Meier (KM) curve was conducted to evaluate the association between the expression level of SERPINH1 and the survival rate [including overall survival (OS) and disease‐free survival (DFS)]. Receiver operator characteristic (ROC) curve and area under the curve (AUC) analyses were applied to detect the optimal cut‐off point that yielded the highest total accuracy with respect to discriminating disease‐free and recurred patients. Statistical analyses were performed with SPSS 19.0 (SPSS Inc., Chicago, IL, USA) and GraphPad Prism 5 (GraphPad Inc., San Diego, CA, USA). Results are expressed as mean ± S.D. A value of *P *<* *0.05 was considered statistically significant.

## Results

### Integrated proteomic and transcriptomic assays in paired ccRCC tissues at four stages

**Figure 1:**
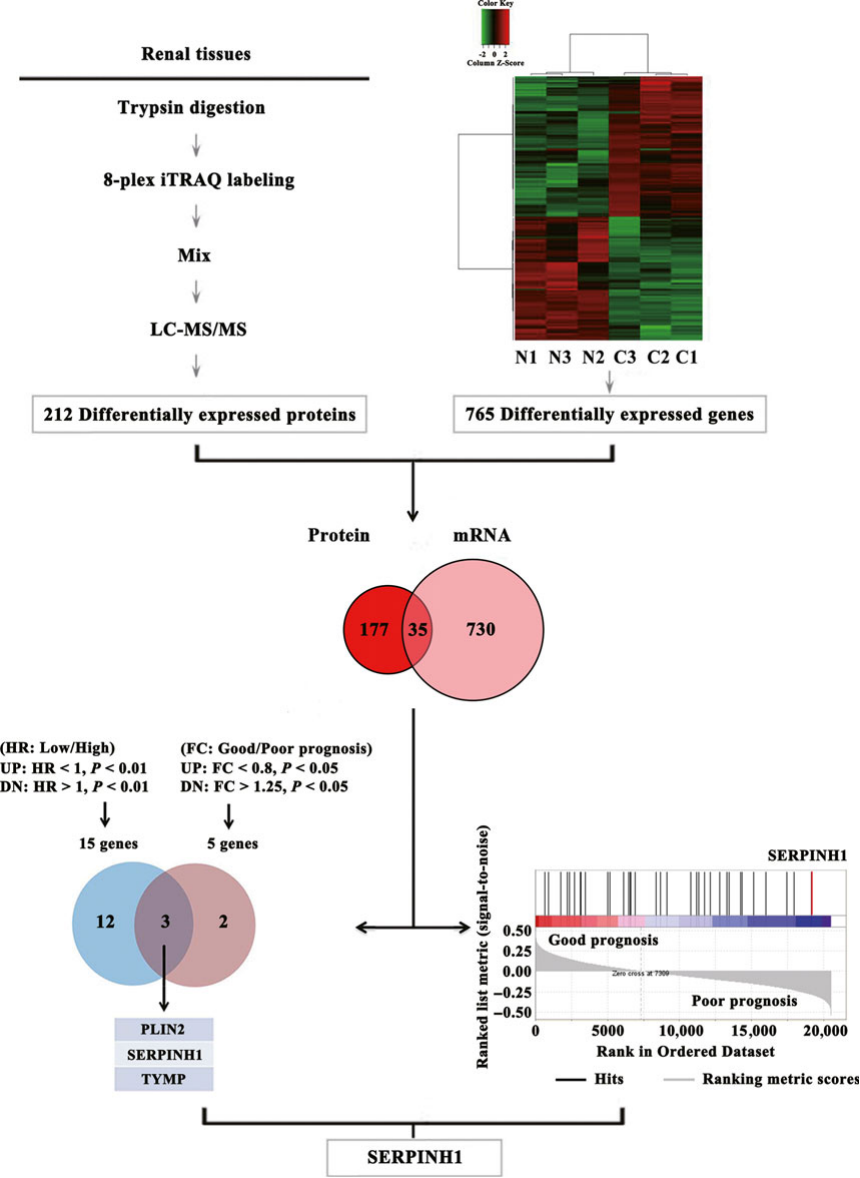
SERPINH1 is identified as potential prognostic marker candidate in ccRCC. Integrated analyses of proteomic and transcriptomic results between paired ccRCC and adjacent normal tissues at four TNM stages identified 35 common DEGs. Integrated univariate Cox analysis, prognosis analysis and GSEA were further used to screen for potential prognostic markers. A Venn diagram showed the overlap between genes that predicted meaningful OS risk ratio (UP, up‐regulated; DN, down‐regulated) and genes associated with OS poor prognosis (FC, fold change) from the TCGA_KIRC data set. The ccRCC GSEA result revealed that the expression of SERPINH1 got the highest ranking metric score among 35 DEGs enriched in poor OS prognosis group.

Using proteomic analysis, we identified 212 proteins differentially expressed between each of four tumour‐node‐metastasis (TNM) stage ccRCC and adjacent normal tissues, which were reported by us previously 9. From the mRNA microarray data set, 2954 gene probes corresponding to 2174 unique genes were found. Of these genes, 765 were differentially expressed at the mRNA level of all four stage paired tissues (Table S1). In total, 35 genes were consistently dysregulated (17 up‐regulated and 18 down‐regulated) at both protein and mRNA levels in ccRCC (Fig. 1 and Table S2), suggesting that these proteins are possibly involved in ccRCC progression and prognosis.

### Association of differentially expressed genes with TGFβ

Because TGFβ plays an important role in EMT process induction and poor prognosis in ccRCC 3, 11, we performed GSEA of 35 DEGs and found that 26 were significantly correlated with TGFβ expression (Fig. S1 and Table S2). This implied the close association of these 26 DEGs with EMT and poor prognosis and suggested that our method could effectively identify prognostic marker candidates. EMT‐related proteins seem to more appropriately act as ccRCC prognostic markers.

### Association of SERPINH1 with poor prognosis of ccRCC patients

We further studied the association of these 35 DEGs with prognosis by analysing the association between expression level and hazard ratio (HR)/poor prognosis of ccRCC. The ccRCC poor prognosis HR analysis of 35 DEGs by univariate Cox regression analysis identified 15 and nine genes with significant HR from overall survival (OS) and disease‐free survival (DFS) data sets, respectively (Fig. 1, Fig. S2A and Table  S3). Meanwhile, we identified five and seven genes differentially expressed between good and poor prognosis groups from OS and DFS data sets, respectively (Fig. 1, Fig. S2A and Table S4). Among them, three genes (*PLIN2*,* SERPINH1* and *TYMP*) and four genes (*P4HB*,* SERPINH1*,* SOD2* and *TYMP*) were correlated with both high HR and poor OS/DFS prognosis, respectively. It can be seen that SERPINH1 and TYMP are consistently associated with the poor OS and DFS prognosis of ccRCC and thus are important for the poor prognosis prediction of ccRCC patients. Both *SERPINH1* and *TYMP* levels are also correlated with *TGF*β expression (Table S2), further indicating the prognostic potential of SERPINH1 and TYMP.

We further analysed the prognosis‐specific enrichment of the 35 genes by GSEA of the TCGA_KIRC data set to objectively test which genes among the 35 DEGs were significantly associated with poor prognosis of ccRCC patients. It was observed that SERPINH1 was significantly enriched and ranked first and second in the poor prognosis group of all four TNM stages of patients for OS and DFS, respectively (Fig. 1, Fig. S2A and Table S5). This reveals that the expression level of SERPINH1 is significantly correlated with the poor prognosis of ccRCC patients (Fig. S2B exhibited increased SERPINH1 level in the poor prognosis patients). Therefore, we focused on the investigation of SERPINH1. Further analyses showed that SERPINH1 was also significantly enriched in the OS and DFS poor prognosis group of early (I or II) and advanced (III or IV) stage patients (Fig. S2C). These results indicate that SERPINH1 is positively associated with the poor prognosis of ccRCC patients.

### Association of SERPINH1 with EMT and regulation of SERPINH1 on the expression of EMT markers

TGFβ can induce the expression of SERPINH1 and play an important role in EMT process induction 3, 12. This reminded that SERPINH1 might mediate TGFβ‐induced EMT process. To verify this speculation, the association was first observed by GSEA in ccRCC. The results showed that the *SERPINH1* mRNA level was positively correlated with levels of the *TGF*β signalling gene set in the EMT process (Fig. S3A). GSEA results further revealed that the *SERPINH1* mRNA level was positively correlated with the EMT phenotype (Fig. S3B) and levels of mesenchymal markers [*VIM, N‐Cadherin, SNAI1* and *FN1*], and negatively correlated with the level of epithelial marker *E‐Cadherin* in ccRCC (Fig. S3C). This suggested a close association between the *SERPINH1* level and EMT phenotype.

To confirm the direct regulatory role of SERPINH1 on the EMT process, we knocked down the expression of SERPINH1 in ccRCC and normal kidney cells. We found that the epithelial marker (E‐Cadherin) was enhanced, and mesenchymal markers (Snail, Vimentin and Slug) were weakened (Fig. S4). These results suggest that SERPINH1 knock‐down reverses the expression of EMT markers, and SERPINH1 possibly affects the prognosis of ccRCC patients by regulating the EMT process.

### Validation of SERPINH1 overexpression in ccRCC

**Figure 2:**
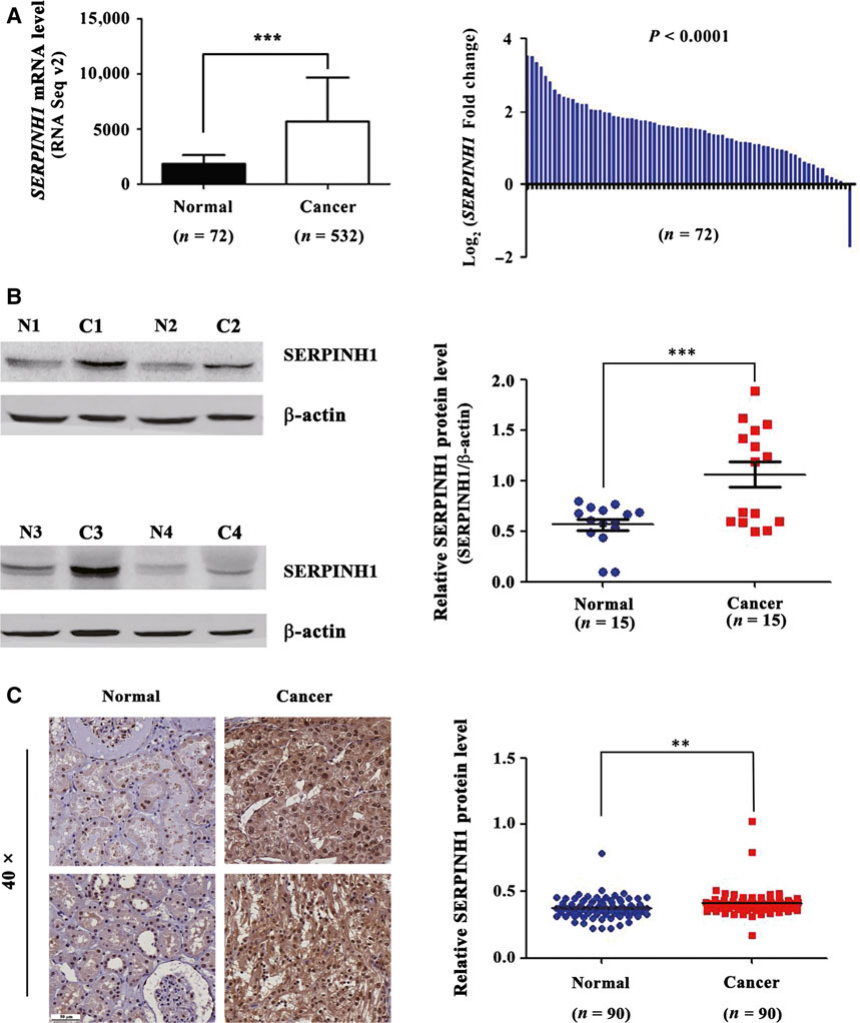
SERPINH1 level is abnormally up‐regulated in ccRCC tissues. (**A**) The mRNA level of *SERPINH1* was up‐regulated in ccRCC tissues of the unpaired (Left) and paired (Right) TCGA_KIRC data sets. ***P <* *0.001. (**B, C**) Abnormal up‐regulation of SERPINH1 level in ccRCC tissues was verified by WB and immunohistochemical analysis. **P <* *0.01, ***P <* *0.001.

To further validate the up‐regulation of the SERPINH1 expression level in ccRCC tissues, we examined both the mRNA level of SERPINH1 in the TCGA_KIRC data set and the protein level in an independent ccRCC set and the THPA data set. Similar to our mRNA microarray results, an increased *SERPINH1* mRNA level was observed in the unpaired and paired TCGA_KIRC data sets (Fig. 2A). In addition, the *SERPINH1* mRNA level could significantly discriminate ccRCC patients from normal individuals (Fig. S5A) and was increased in all four TNM stages of ccRCC tissues (Fig. S5B). Importantly, consistent with iTRAQ results, WB and tissue microarray (TMA) staining results from the independent ccRCC set (Fig. 2B and C) and IHC results from the THPA data set (Fig. S5C) further verified the up‐regulated protein level of SERPINH1 in ccRCC tissues.

### Association of SERPINH1 with poor clinical outcome of ccRCC patients

**Figure 3:**
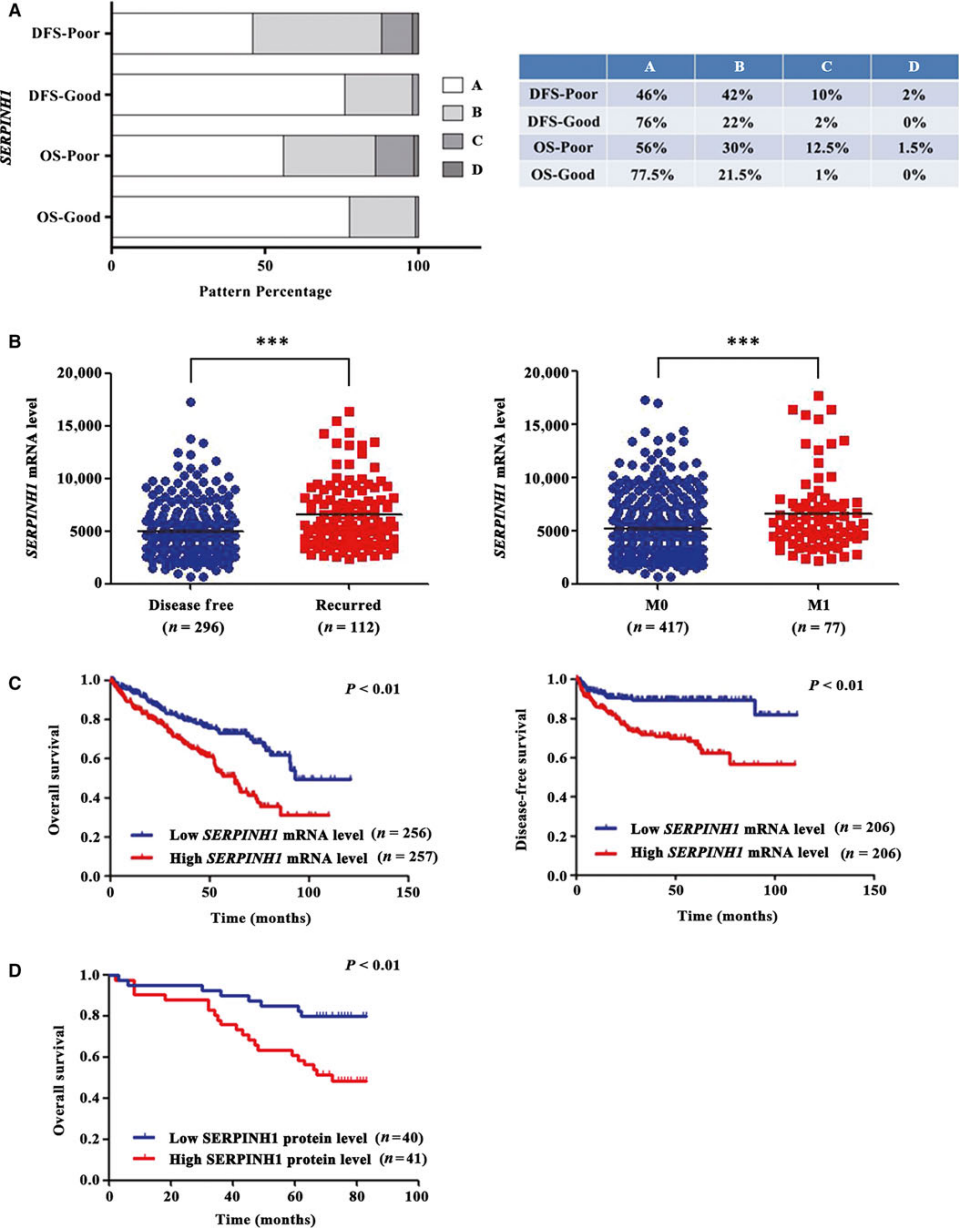
SERPINH1 is correlated with poor clinical outcome of ccRCC patients. (**A**) Stacked bar graphs showing differential mRNA levels of *SERPINH1* in patients with good and poor prognosis. Higher *SERPINH1* levels were associated with poor prognosis of patients. Expression level was quantified in a four‐tier scale by the K‐means cluster method (from A with the lowest level to D with the highest level). The table shows the details (right). (**B**) Scatter plot displaying the level of *SERPINH1* in patients with/without recurrence or metastasis. ***P <* *0.001. (**C**) Kaplan–Meier (KM) curves indicated a shorter OS and DFS time with a high *SERPINH1* mRNA level. (**D**) The KM curve indicated a shorter OS time with a high SERPINH1 protein level; *P* values were calculated with a log‐rank test.

To further determine that SERPINH1 had potential as a prognostic marker, we performed additional analyses on the TCGA_KIRC data set by analysing the expression level of *SERPINH1* in ccRCC patients with OS and DFS poor/good prognosis (*n *=* *80/102 and 59/85, respectively) (Fig. 3A, A: low; B: medium; C: high; D: highest). In ccRCC patients with poor prognosis, significantly, the percentage of ccRCC cases displaying the C and D levels of *SERPINH1* expression was drastically higher than that in the cases with good prognosis, suggesting the decent prognostic value of SERPINH1 for ccRCC patients.

To validate the above findings, a clinical outcome study was conducted on the TCGA_KIRC data set and TMA patients. The *SERPINH1* expression level was observed to significantly correlate with T‐stage progression (Fig. S5D), recurrence and metastasis of ccRCC patients (Fig. 3B). These results indicate that a high level of *SERPINH1* could predict poor clinical outcome of ccRCC patients. To further investigate the association of SERPINH1 with the survival of ccRCC patients, KM curves were plotted. The results showed that both high mRNA and protein levels of SERPINH1 were significantly associated with shorter OS and DFS time of ccRCC patients (*P *<* *0.01, Fig. 3C and D). In addition, a high level of SERPINH1 could predict the poor prognosis in early‐stage ccRCC patients (Fig. S6), which is very important in identifying ccRCC patients in need of early aggressive management.

### SERPINH1 as a potential independent prognostic marker in ccRCC

**Figure 4:**
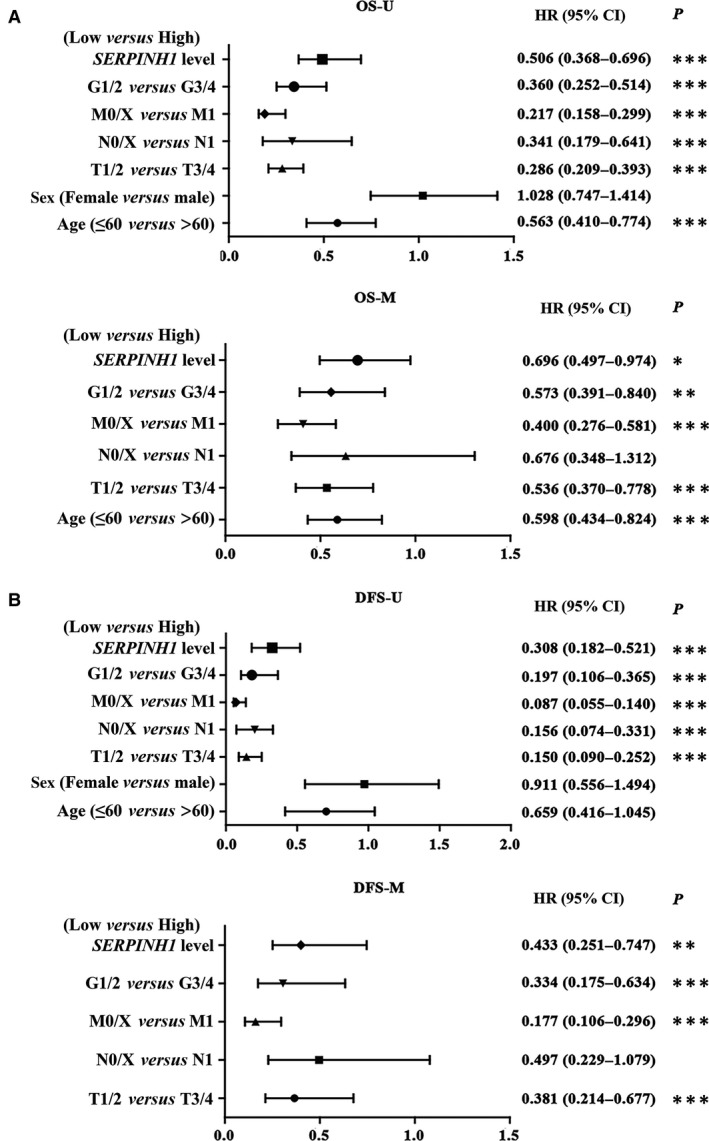
SERPINH1 is a potential independent prognostic marker in ccRCC. *SERPINH1* was an independent OS (**A**) and DFS (**B**) prognostic factor in ccRCC. U (univariate) and M (multivariate) Cox regression analyses. *P <* *0.05, **P <* *0.01, ***P <* *0.001.

To elucidate whether SERPINH1 was a potential independent prognostic marker in ccRCC, univariate and multivariate Cox models were employed for further analysis of the OS and DFS of ccRCC patients from the TCGA data set. Factors including *SERPINH1* expression level, grade, TNM stage, sex and age were recruited as cofactors. The results revealed that the *SERPINH1* expression level indeed predicted the OS and DFS time of ccRCC patients and could independently predict the prognosis of ccRCC patients (Low *versus* High; HR 0.696 and 0.433, 95% CI: 0.497–0.974 and 0.251–0.747, respectively, both *P *<* *0.05, Fig. 4).

### Comparison between SERPINH1 and reported markers

To investigate whether SERPINH1 is superior in predicting the prognosis of ccRCC patients, we compared it with eight reported biomarkers: HADHA 13, DIABLO 14, PDZK1 9, LDHA 15, BIRC5 16, CA9 17, FSCN2 18 and IMP3 19. Figure S7A shows that the *HADHA* level failed to discriminate between patients with/without recurrence or metastasis. The *DIABLO* level was increased in recurrent or metastatic patients, which was contradictory with the decreased level of *DIABLO* in ccRCC tissues. Compared with *PDZK1*,* SERPINH1* presented a stronger capability in predicting recurrence in ccRCC patients (Fig. S7B), which was also a prominent characteristic of *SERPINH1* compared with other four reported markers (Fig. S8). The *BIRC5* level was able to discriminate between patients with and without recurrence/metastasis, but its very low expression abundance made it difficult to be detected using conventional methods.

### SERPINH1 as a potential independent prognostic marker in ccRCC without *VHL* mutations

**Figure 5:**
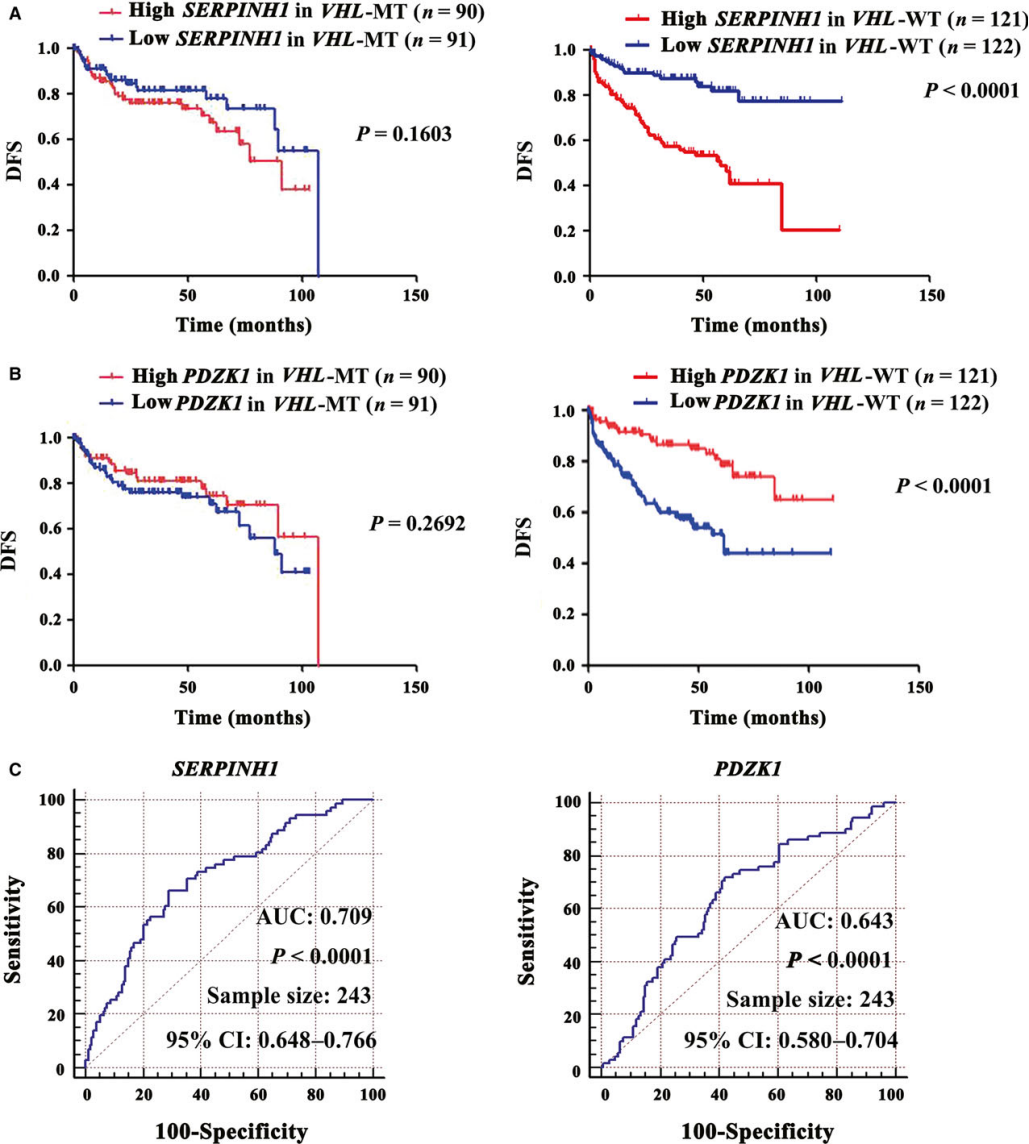
SERPINH1 predicts the DFS prognosis of *VHL*‐WT ccRCC. (**A**) The *SERPINH1* mRNA level predicted the DFS prognosis of patients with *VHL*‐WT ccRCC better than that of patients with *VHL*‐MT. (**B**) The *PDZK1* mRNA level also better predicted the DFS prognosis of patients with *VHL*‐WT. *P* values were calculated with a log‐rank test. (**C**) Receiver operator characteristic (ROC) curve results revealed that the *SERPINH1* mRNA level had a stronger DFS prognosis‐predicting ability for *VHL*‐WT ccRCC than *PDZK1*. The area under curve (AUC) and the corresponding 95% CI are shown in the plots.

EMT‐related proteins often act as prognostic markers 5. We also identified a novel EMT‐related potential prognostic marker, SERPINH1. More significantly, whether SERPINH1 could predict the prognosis of specific subgroup more precisely? Because TGFβ, which induces the expression of SERPINH1 and EMT in ccRCC, is frequently suppressed by VHL in ccRCC cells 11, we speculated that the SERPINH1 level was increased in patients with *VHL*‐mutant (MT) and had the more significant prognosis value. However, the SERPINH1 level in the TCGA_KIRC data set showed no difference between the *VHL*‐WT and *VHL*‐MT subgroups Table S6, (Fig. S9A). Therefore, we anticipated that the SERPINH1 level would have a similar ability in predicting the prognosis of patients with *VHL*‐WT and *VHL*‐MT. However, we observed that the SERPINH1 level could predict the OS (Fig. S9B) and DFS (Fig. 5A) prognosis better in patients with *VHL*‐WT than in patients with *VHL*‐MT. Again, SERPINH1 had a stronger DFS prognosis‐predicting ability than PDZK1 (Fig. 5B and C). Meanwhile, multivariate Cox analysis further revealed that SERPINH1 was a potential independent prognostic marker for DFS in patients with *VHL*‐WT ccRCC (Low *versus* High; Univariate and Multivariate; HR = 0.230 and 0.362, 95% CI: 0.129–0.411 and 0.195–0.672, *P *<* *0.001 and 0.01, respectively Fig. S10).

## Discussion

SERPINH1/HSP47 is highly expressed in a wide variety of cancers, including pancreatic cancer and glioma 20, 21. It can drive the malignant behaviour of cancer cells 12, 21 and predict the metastatic activity of human cancer cells 22. Likewise, SERPINH1 expression in ccRCC tissues is up‐regulated in a grade‐dependent manner 23. However, less is known about the clinical significance of SERPINH1 expression in ccRCC. In this study, we have demonstrated that SERPINH1 is overexpressed at the four TNM stages of ccRCC tissues and closely correlates with poor clinical outcome in all‐stage ccRCC patients. This is especially evident in the early stage of ccRCC and even in *VHL*‐WT ccRCC. SERPINH1 regulates the expression of EMT‐related proteins, closely correlates with the EMT phenotype and affects the prognosis of ccRCC patients. Moreover, SERPINH1 presents a superior capability in predicting recurrence of ccRCC and could serve as a potential independent prognostic marker in *VHL*‐WT ccRCC. As far as we know, this is the first report dealing with the clinical significance of SERPINH1 expression in the prognosis prediction of ccRCC patients. Our findings could provide aid in improving the prognosis prediction system of ccRCC patients. The role of SERPINH1 in the prognostic judgement of *VHL*‐WT ccRCC will deepen our understanding in the pathogenesis of *VHL*‐WT ccRCC.

TGFβ signalling is a common factor contributing to the ccRCC EMT phenotype and poor prognosis in ccRCC 3, 24. TGFβ‐induced SERPINH1/HSP47 expression correlates with poor prognosis of colorectal and breast cancer patients 12, 25. We found that the *SERPINH1* level positively correlated with *TGF*β expression in ccRCC. Functionally, dysregulation of SERPINH1/HSP47 stimulates expression of extracellular matrix (ECM) proteins, including collagen type I that could induce EMT. SERPINH1/HSP47 as a collagen‐specific chaperone plays a critical role in the assembly of triple helices in procollagen. Deficiency of this chaperone leads to a loss of correctly folded triple‐helical type I collagen 26. Conversely, overexpressing SERPINH1/HSP47 increases procollagen expression 27, thereby promoting EMT 28, 29. Indeed, SERPINH1/HSP47 can regulate the expression of ECM proteins 12 and EMT‐related protein fibronectin (FN), which can act as a ccRCC prognostic marker 30. Excessive collagen deposition is frequently seen in a variety of diseases, including cancers 31. SERPINH1/HSP47 is extensively expressed in the intratumoral and peritumoral stroma/fibrotic areas of pancreatic cancer samples; the link between SERPINH1/HSP47 expression and pancreatic cancer development is further evidenced by the observation that SERPINH1/HSP47 is progressively up‐regulated from noninvasive pancreatic to intraepithelial neoplasia 20. We found SERPINH1 up‐regulation and its close association with the expression of the EMT‐related gene set and EMT markers in ccRCC. Our data support the notion that dysregulation of SERPINH1/HSP47 induces EMT, which is closely associated with ccRCC development and progression, and further affects the prognosis of ccRCC.

It is significant that we identified the overexpression of the *SERPINH1* gene in 123 pairs of primary ccRCC tissues at the four TNM stages and the close association of overexpressed SERPINH1 with poor OS and DFS prognosis. Importantly, our results showed the prognosis of a ccRCC subgroup, patients with *VHL*‐WT, can be precisely predicted by SERPINH1 overexpression. For this specific patient group, TGFβ‐induced SERPINH1 plays an important role in their prognosis judgement, even in their tumorigenesis. VHL can attenuate TGFβ signalling and EMT in ccRCC 11. The SERPINH1 level was found to better predict prognosis of patients with *VHL*‐WT than patients with *VHL*‐MT in this study. One possible explanation is that in patients with *VHL*‐WT, SERPINH1 exerts its EMT‐enhancing function, followed by promoting ccRCC progression; in patients with *VHL*‐MT, however, *VHL* mutation‐mediated hypoxia‐inducible factor 1α (HIF1α) accumulation and TGFβ signalling activation exert a prominent function over SERPINH1. Our speculation is reasonable because *VHL* loss could stabilize HIF1α and lead to activation of HIF‐response genes, including *TGF* as well as *VEGF* and *PDGF* 32. In addition, we found that SERPINH1 was a superior DFS prognosis predictor of ccRCC than the previously reported biomarkers. Thus, our findings help to improve the prognosis prediction system of ccRCC. Moreover, for EMT‐related and VHL‐regulated molecules, we only found a novel prognosis‐predicting molecule SERPINH1. Whether other related molecules have the same or similar role remains to be investigated.

Antagonizing TGFβ1 *in vivo* can suppress RCC tumorigenesis and regress established 786‐O tumours in athymic mice 33. Blocking TGFβ has been shown to have anticancer activities in preclinical cancer models 34. We suggest that SERPINH1/HSP47 is also an attractive and ideal target for EMT blocking therapy in *VHL*‐WT ccRCC because it exhibits the role of driver gene in this study and is specifically expressed in collagen‐producing cells 35. Recent studies have shown that silencing SERPINH1 by small molecules can suppress cancer cell phenotypes 36, 37 that lead to poor prognosis. It is thus clear that finding highly specific inhibitors of SERPINH1/HSP47, including small molecules, has broad therapeutic applicability in *VHL*‐WT ccRCC.

Obviously, high SERPINH1/HSP47 levels at all four TNM stages of ccRCC are closely correlated with poor clinical outcome in all‐stage ccRCC patients, as well as with the EMT phenotype. SERPINH1 presents a superior capability in predicting recurrence of ccRCC and could serve as a potential independent prognostic marker in the patients with *VHL*‐WT ccRCC. Our findings could aid in improving the prognosis prediction system of ccRCC patients. Because we do not have enough specimens with lymph node/distant metastasis or recurrence and DFS time in the independent validation set of 81 samples, we cannot discriminate the patients with lymph node involvement, distant metastasis or recurrence. Future basic and clinical research will allow us to address these limitations. Large prospective studies are also expected to confirm our findings.

## Conflict of interests

The authors declare no conflict of interests.

## Supporting information


**Figure S1.** The levels of 35 differentially expressed genes are significantly correlated with the level of TGFβ in ccRCC.

**Figure S2.** SERPINH1 is correlated with poor prognosis of all stage ccRCC patients.

**Figure S3.** *SERPINH1* is positively correlated with TGFβ signaling in EMT and the level of EMT gene set/EMT markers in ccRCC.

**Figure S4.** SERPINH1 regulates the expression of EMT markers in ccRCC and HEK293 cell lines.

**Figure S5.** SERPINH1 expression level can discriminate normal and tumor tissues and its expression level is abnormally upregulated in ccRCC tissues as T stage progresses.

**Figure S6.** SERPINH1 is correlated with poor clinical outcome of early stage ccRCC patients.

**Figure S7.** External comparison with reported prognostic markers‐1.

**Figure S8.** External comparison with reported prognostic markers‐2.

**Figure S9.** SERPINH1 level shows no difference between *VHL*‐WT and *VHL*‐MT patients and predicts the OS prognosis of *VHL*‐WT ccRCC patients.

**Figure S10**. SERPINH1 is an independent DFS prognostic marker in *VHL*‐WT ccRCC patients.

**Table S1.** List of genes differentially expressed between ccRCC and adjacent normal tissues by mRNA microarray analysis.

**Table S2.** Consistently dysregulated 35 genes and their correlation with TGFβ level.

**Table S3.** The genes significantly correlated with poor prognosis by univariate cox regression analysis.

**Table S4.** The genes significantly differentially expressed between patients with good and poor prognosis.

**Table S5.** GSEA rank score of 35 genes for OS and DFS prognosis in ccRCC samples.

**Table S6.** *VHL* mutation in TCGA_KIRC dataset.

** **
