# Supplementary figures and images for: SERPINH1 overexpression in clear cell renal cell carcinoma: association with poor clinical outcome and its potential as a novel prognostic marker
Source: J Cell Mol Med. 2017 Dec 14;22(2):1224–35. doi: 10.1111/jcmm.13495 (PMC5783852; doi:10.1111/jcmm.13495)

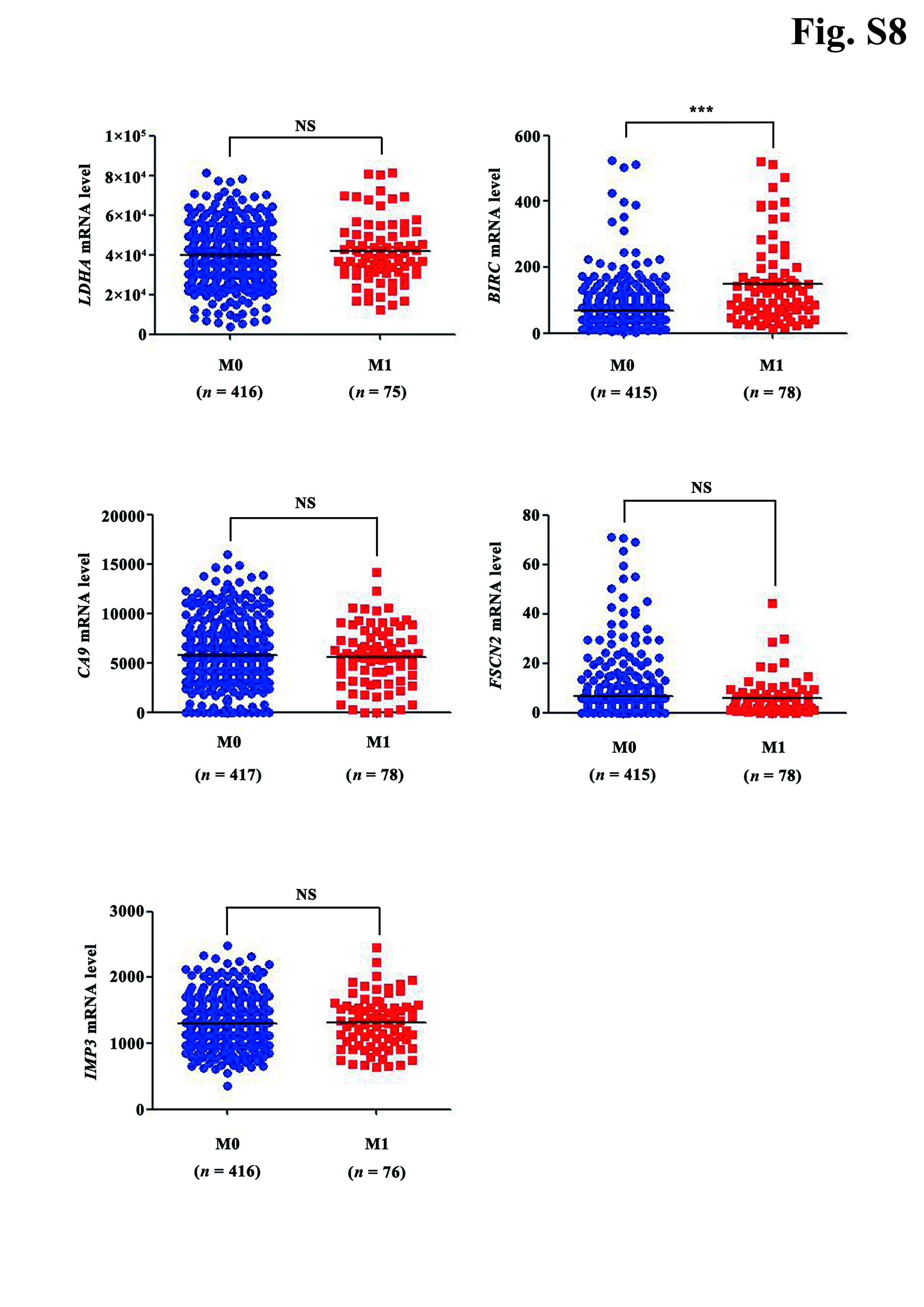

Supplement: Supplementary file 8 — Figure S8. External comparison with reported prognostic markers‐2. [file JCMM-22-1224-s008.tif]

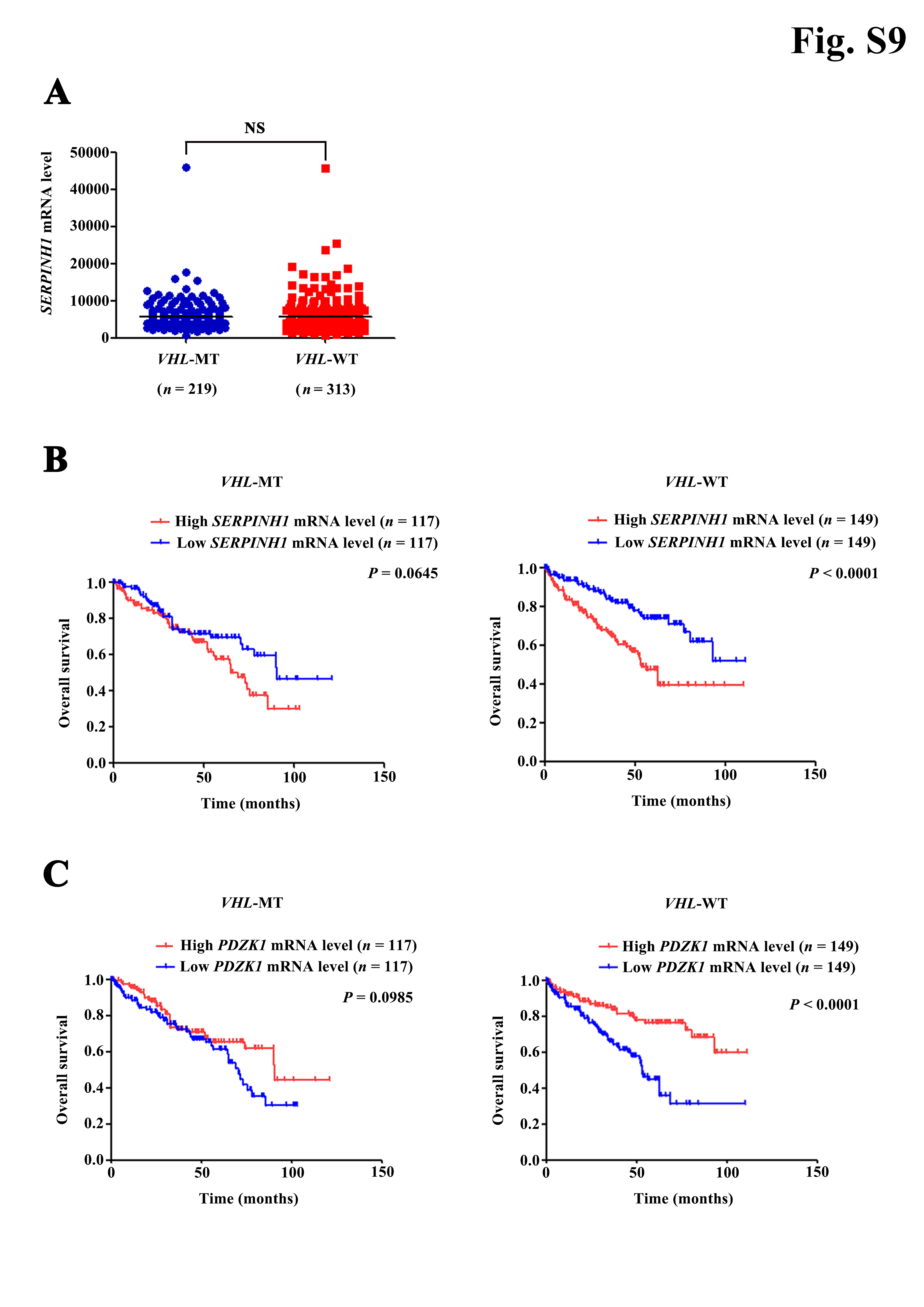

Supplement: Supplementary file 9 — Figure S9. SERPINH1 level shows no difference between VHL‐WT and VHL‐MT patients and predicts the OS prognosis of VHL‐WT ccRCC patients. [file JCMM-22-1224-s009.tif]
