# Supplementary material for: SERPINH1 overexpression in clear cell renal cell carcinoma: association with poor clinical outcome and its potential as a novel prognostic marker
Source: J Cell Mol Med. 2017 Dec 14;22(2):1224–35. doi: 10.1111/jcmm.13495 (PMC5783852; doi:10.1111/jcmm.13495)
Supplement: Supplementary file 11 — Table S1. List of genes differentially expressed between ccRCC and adjacent normal tissues by mRNA microarray analysis. [file JCMM-22-1224-s011.doc]

Supplementary Table 1 List of genes differentially expressed between ccRCC and adjacent normal tissues by mRNA microarray analysis

|  | | **Normalized Intensity** | | | | | |  | | **log2 (Ratio)** | | | ***P* value** | | | |
| --- | --- | --- | --- | --- | --- | --- | --- | --- | --- | --- | --- | --- | --- | --- | --- | --- |
| **Gene_symbol** | C1 | | C2 | C3 | N1 | N2 | N3 | | C1/N1 | | C2/N2 | C3/N3 | | C1 *vs.* N1 | C2 *vs.* N2 | C3 *vs.* N3 |
| **ABCA1** | 1436.19934 | | 2629.41284 | 2417.66357 | 425.014282 | 659.802368 | 457.519653 | | 2.148054962 | | 2.059297047 | 2.565470236 | | 0.00030511 | 0.000312274 | 6.71719E-05 |
| **ACACB** | 1302.53503 | | 1539.03003 | 1879.6897 | 6010.02588 | 3917.57666 | 4318.43896 | | -1.862691014 | | -1.261072772 | -1.304241045 | | 0.000573877 | 0.008159836 | 0.006914557 |
| **ACAD11** | 552.171631 | | 1333.63672 | 305.311646 | 120.898491 | 85.7096252 | 140.670227 | | 2.54337298 | | 3.903996369 | 1.677686334 | | 0.000245567 | 8.50609E-06 | 0.010310761 |
| **ACAT1** | 1214.96191 | | 1211.53662 | 240.938705 | 5546.37402 | 8610.58301 | 2354.51904 | | -1.850201027 | | -2.709017183 | -3.044206641 | | 0.000612558 | 3.20668E-05 | 2.66344E-05 |
| **ACLY** | 14812.7646 | | 17165.0117 | 20059.5254 | 3600.78076 | 3742.1145 | 3814.48462 | | 2.404068626 | | 2.289176975 | 2.174306705 | | 7.27713E-05 | 0.000103849 | 0.000153188 |
| **ACOT11** | 652.650269 | | 820.626709 | 902.140686 | 6721.70654 | 3557.75806 | 3430.71436 | | -2.985560525 | | -2.026814563 | -1.907154617 | | 1.86953E-05 | 0.00031596 | 0.000965345 |
| **ACPP** | 126.289673 | | 140.083908 | 203.234558 | 2390.54956 | 1076.2218 | 3748.92969 | | -3.905226778 | | -2.97185619 | -4.001738256 | | 6.6891E-06 | 3.86741E-05 | 4.84537E-06 |
| **ACSM5** | 1079.4541 | | 1140.5481 | 1269.06641 | 246.782486 | 84.0924606 | 228.076965 | | 2.530056725 | | 3.709831333 | 2.809015693 | | 0.000120622 | 1.24275E-05 | 5.36218E-05 |
| **ADAM28** | 566.920776 | | 1166.10388 | 2430.53809 | 310.347656 | 384.884735 | 293.632019 | | 1.297568563 | | 1.631547334 | 3.274794034 | | 0.013837012 | 0.00229098 | 1.38563E-05 |
| **ADAMTSL2** | 396.383636 | | 242.307312 | 754.082947 | 4695.09912 | 2376.42065 | 3585.04199 | | -3.181420482 | | -3.289695689 | -2.208478346 | | 1.40166E-05 | 1.27845E-05 | 0.00018905 |
| **ADAP2** | 1147.66895 | | 3185.96265 | 4654.16309 | 721.651794 | 483.531647 | 912.307861 | | 1.047856817 | | 2.768274733 | 2.332179321 | | 0.028253891 | 3.51238E-05 | 0.000108357 |
| **ADPRHL2** | 1727.49524 | | 1489.8114 | 2578.59595 | 1174.0863 | 742.27771 | 813.97522 | | 0.911533096 | | 1.078248197 | 1.728765593 | | 0.046737652 | 0.022690954 | 0.00115734 |
| **ADRA2C** | 128.133316 | | 122.100166 | 84.6044312 | 597.01416 | 650.099426 | 342.798309 | | -1.838225645 | | -2.460350794 | -1.477920089 | | 0.002416301 | 0.000212572 | 0.016985519 |
| **AGAP3** | 586.279053 | | 644.575317 | 1074.1084 | 383.88385 | 232.062851 | 502.588745 | | 1.035492464 | | 1.478136207 | 1.350544882 | | 0.03850266 | 0.006145501 | 0.007992671 |
| **AGPAT9** | 147.491592 | | 202.553772 | 79.0867462 | 513.506958 | 1603.41687 | 1528.2522 | | -1.389934379 | | -2.994061559 | -3.94673201 | | 0.013422503 | 2.76599E-05 | 1.01381E-05 |
| **AGXT2L2** | 2650.2395 | | 3020.32275 | 4445.41113 | 1124.23132 | 760.066467 | 1149.94495 | | 1.593111118 | | 2.069913533 | 1.908603004 | | 0.001986863 | 0.000287606 | 0.000490391 |
| **AK3** | 1550.50537 | | 1444.37878 | 1976.24915 | 5187.41748 | 4012.98926 | 4505.54443 | | -1.402925114 | | -1.383798585 | -1.305189674 | | 0.004265497 | 0.004594761 | 0.006835178 |
| **AKAP13** | 818.578308 | | 968.282715 | 1016.17279 | 361.449097 | 505.363342 | 312.752228 | | 1.590377995 | | 0.988264945 | 2.031902826 | | 0.003392307 | 0.03792746 | 0.000581873 |
| **ALAS1** | 492.253174 | | 496.919281 | 303.472412 | 1632.75281 | 2507.41089 | 1106.24158 | | -1.346725515 | | -2.293985858 | -1.543782499 | | 0.007473473 | 0.000139268 | 0.004196399 |
| **ALDH1A2** | 129.976959 | | 136.297867 | 106.675148 | 2682.20142 | 2320.62842 | 1776.81506 | | -4.024890667 | | -4.132736597 | -3.753134825 | | 5.44144E-06 | 4.18072E-06 | 1.08855E-05 |
| **ALDOC** | 3443.00684 | | 3722.6355 | 1290.21753 | 767.7677 | 903.185364 | 576.338196 | | 2.53650111 | | 2.138590251 | 1.372101893 | | 6.2794E-05 | 0.000213055 | 0.006740992 |
| **ANGPTL4** | 11402.9434 | | 11379.9248 | 3716.15771 | 264.23175 | 115.627136 | 338.701111 | | 5.820935074 | | 6.608489415 | 3.613769278 | | 1.03048E-06 | 8.53312E-07 | 6.78494E-06 |
| **ANKHD1** | 505.158691 | | 610.500854 | 780.75177 | 230.57959 | 240.148666 | 435.667969 | | 1.56705599 | | 1.348174497 | 1.16027661 | | 0.005432331 | 0.014638071 | 0.020694044 |
| **ANKRD33B** | 1152.27808 | | 1379.06934 | 1096.17908 | 89.7390823 | 79.2409744 | 228.076965 | | 4.002218607 | | 4.06468122 | 2.616055481 | | 8.6587E-06 | 6.96043E-06 | 9.63272E-05 |
| **ANKS1B** | 1046.26843 | | 1487.91833 | 2328.46094 | 473.622925 | 175.462158 | 692.425232 | | 1.537918758 | | 3.061314242 | 1.85079127 | | 0.003597712 | 2.76316E-05 | 0.000732825 |
| **ANXA3** | 294.06134 | | 367.247009 | 521.420776 | 1267.56458 | 4009.75488 | 1267.39771 | | -1.706615668 | | -3.396442729 | -1.050847346 | | 0.00296924 | 8.85295E-06 | 0.029904023 |
| **ANXA4** | 20306.8262 | | 20858.3047 | 19334.8711 | 4005.85303 | 6716.88525 | 3941.49756 | | 2.721777842 | | 1.738420385 | 2.067318516 | | 2.90546E-05 | 0.000827687 | 0.000225672 |
| **ANXA9** | 84.807663 | | 114.528069 | 194.038422 | 695.477905 | 393.779114 | 554.486511 | | -2.682873154 | | -1.87269429 | -1.058503503 | | 0.000192012 | 0.00219325 | 0.044613719 |
| **AOAH** | 2193.01562 | | 2176.97974 | 5820.23291 | 582.057678 | 615.330383 | 524.44043 | | 2.297806312 | | 1.880074232 | 3.517946867 | | 0.000151108 | 0.000648837 | 6.8947E-06 |
| **APLN** | 2198.54639 | | 3280.61377 | 1229.52307 | 363.941833 | 105.924156 | 644.624695 | | 2.987290473 | | 4.907324557 | 1.128717314 | | 2.51017E-05 | 2.10021E-06 | 0.019904986 |
| **APOBEC3F** | 242.439301 | | 339.798157 | 511.305023 | 139.594131 | 104.306992 | 147.498871 | | 1.166155991 | | 1.605538342 | 2.280933544 | | 0.044121854 | 0.007213107 | 0.000537585 |
| **APOC1** | 13560.9297 | | 12043.4307 | 41353.1719 | 614.46344 | 459.2742 | 644.624695 | | 4.859148195 | | 4.784824472 | 5.960644739 | | 1.55676E-06 | 1.65686E-06 | 2.2516E-05 |
| **APOD** | 247.048416 | | 279.221313 | 276.803619 | 5763.24316 | 3970.13452 | 2466.50879 | | -4.153260732 | | -3.786667729 | -2.928437045 | | 3.38358E-06 | 5.03832E-06 | 3.27897E-05 |
| **APOLD1** | 13343.3799 | | 10757.1191 | 15387.8906 | 886.173462 | 1599.3739 | 3258.63257 | | 4.291226783 | | 2.859934174 | 2.018840613 | | 2.3922E-06 | 2.17987E-05 | 0.000274426 |
| **AQP3** | 1625.17297 | | 2329.36841 | 2317.42578 | 7863.38721 | 12369.6777 | 13407.374 | | -1.911061599 | | -2.308136491 | -2.70984622 | | 0.000453427 | 9.92764E-05 | 3.19615E-05 |
| **ARHGAP25** | 1314.5188 | | 1347.83447 | 1762.8988 | 250.521606 | 390.5448 | 299.09494 | | 2.789562755 | | 1.816183629 | 2.823725287 | | 5.38208E-05 | 0.001027382 | 4.12417E-05 |
| **ARHGAP26** | 729.16156 | | 910.545471 | 866.275757 | 215.623077 | 472.211517 | 378.307281 | | 2.168265457 | | 0.991525151 | 1.519227039 | | 0.000489568 | 0.038151078 | 0.004438613 |
| **ARHGAP8** | 958.695312 | | 705.152161 | 483.716614 | 5273.41748 | 2463.74731 | 2422.80542 | | -2.110657862 | | -1.733688724 | -2.166605406 | | 0.000232358 | 0.001092969 | 0.00025921 |
| **ARL4C** | 1574.47266 | | 1305.24133 | 2111.43237 | 356.463593 | 366.287354 | 622.77301 | | 2.536749998 | | 1.861220845 | 1.891080787 | | 8.95661E-05 | 0.000881934 | 0.000649896 |
| **ARNT2** | 420.351013 | | 330.333008 | 188.520737 | 1457.01367 | 1965.66125 | 693.791016 | | -1.404638628 | | -2.553934226 | -1.459350142 | | 0.006127648 | 7.2985E-05 | 0.008437457 |
| **ARRDC2** | 493.174988 | | 553.710083 | 980.307861 | 90.9854584 | 71.9637375 | 240.36853 | | 2.794930403 | | 2.875292823 | 2.388549006 | | 0.000164727 | 0.000123743 | 0.000195765 |
| **ATP12A** | 86.6513062 | | 97.4908295 | 125.98703 | 665.56488 | 794.026917 | 706.082581 | | -2.586675299 | | -3.074656235 | -2.058278125 | | 0.000255148 | 4.24622E-05 | 0.001085052 |
| **ATP1A1** | 7499.02539 | | 7190.65869 | 16452.8027 | 55943.5938 | 30972.709 | 29731.9492 | | -2.506731654 | | -2.046668259 | -1.188035053 | | 5.08911E-05 | 0.000274684 | 0.010426315 |
| **ATP1A1** | 1800.31921 | | 2163.72852 | 1796.00488 | 14838.1084 | 23034.8652 | 4343.02246 | | -2.706544533 | | -3.37007436 | -1.372563734 | | 3.17026E-05 | 7.30619E-06 | 0.005048303 |
| **ATP2B4** | 1414.99744 | | 1037.37817 | 1577.13696 | 458.666412 | 547.409546 | 580.435364 | | 2.015440713 | | 0.979410674 | 1.62305056 | | 0.000484697 | 0.038573433 | 0.002135599 |
| **ATP5B** | 11841.7305 | | 9202.94531 | 3603.96484 | 40164.4727 | 42895.2422 | 12376.248 | | -1.393111129 | | -2.231478695 | -2.013990744 | | 0.003889557 | 0.00012129 | 0.000288942 |
| **ATP5G3** | 1195.60364 | | 735.440552 | 203.234558 | 4503.15723 | 6886.6875 | 1323.3927 | | -1.571295418 | | -3.100763437 | -2.370647019 | | 0.002057481 | 1.35259E-05 | 0.000214701 |
| **ATP6V0D1** | 805.672791 | | 986.266479 | 454.289001 | 2923.99854 | 2294.75391 | 1488.646 | | -1.499616193 | | -1.149317377 | -1.48374997 | | 0.003128098 | 0.014895246 | 0.004441843 |
| **ATP6V0E1** | 9690.19727 | | 11424.4111 | 25905.5078 | 5115.12793 | 4295.18408 | 7510.15088 | | 1.26564811 | | 1.55045785 | 1.484706124 | | 0.01039839 | 0.002661146 | 0.002559462 |
| **ATP6V1A** | 4776.88379 | | 6045.37793 | 3656.38281 | 13518.1953 | 21347.3555 | 9138.10156 | | -1.159641813 | | -1.718449475 | -1.548295564 | | 0.012284597 | 0.001519423 | 0.002050743 |
| **AVPR2** | 607.480957 | | 786.552246 | 624.41748 | 3407.59253 | 1774.02747 | 2136.0022 | | -2.115573272 | | -1.104360053 | -1.642651108 | | 0.000259946 | 0.019262003 | 0.00192998 |
| **AXL** | 1398.40466 | | 1464.25549 | 2201.55444 | 941.013977 | 747.12915 | 852.215698 | | 0.93795819 | | 1.044294261 | 1.446682544 | | 0.043374881 | 0.030274339 | 0.004060285 |
| **B3GNT5** | 273.78125 | | 793.177856 | 452.449768 | 84.7535782 | 178.696472 | 157.05899 | | 2.060344281 | | 2.138454819 | 2.029937853 | | 0.002537079 | 0.000493023 | 0.001305639 |
| **BAG1** | 743.910706 | | 572.64032 | 118.630127 | 2828.02759 | 4911.32324 | 1111.70447 | | -1.562624797 | | -3.004743982 | -2.860064859 | | 0.002404402 | 1.75882E-05 | 7.79246E-05 |
| **BAIAP3** | 504.236877 | | 371.033081 | 405.5495 | 2142.52051 | 749.554932 | 1364.3645 | | -1.706675462 | | -0.978399476 | -1.493116073 | | 0.001462903 | 0.042587522 | 0.00448863 |
| **BCAM** | 2023.40027 | | 1604.33948 | 1989.12366 | 8394.34375 | 3934.55688 | 8949.63086 | | -1.68459919 | | -1.208066157 | -2.32474176 | | 0.001139584 | 0.010461303 | 0.000102407 |
| **BCKDHB** | 239.673828 | | 286.793427 | 173.806931 | 909.854614 | 902.37677 | 573.606689 | | -1.504999879 | | -1.62652441 | -1.268548449 | | 0.005411543 | 0.00259895 | 0.020183357 |
| **BCL6** | 2815.24561 | | 3334.56519 | 9229.23926 | 497.304077 | 1144.95117 | 1651.16797 | | 2.887947944 | | 1.627156632 | 2.344480614 | | 2.78099E-05 | 0.001611557 | 9.26705E-05 |
| **BET1L** | 4052.3313 | | 4702.27637 | 5081.7832 | 1120.49219 | 2445.1499 | 2064.98413 | | 2.208266683 | | 1.043352561 | 1.162686991 | | 0.000165184 | 0.022383714 | 0.012923807 |
| **BHLHE41** | 3480.80151 | | 4232.80566 | 2381.79858 | 168.260788 | 448.762665 | 430.205048 | | 4.712379764 | | 3.302021138 | 2.643427125 | | 2.29878E-06 | 1.05949E-05 | 5.49024E-05 |
| **BIK** | 205.566406 | | 246.093369 | 170.128479 | 1088.08643 | 912.079773 | 848.11853 | | -1.991846954 | | -1.873160386 | -1.9232926 | | 0.000782925 | 0.001003441 | 0.001360759 |
| **BMP2K** | 670.164917 | | 1003.30371 | 1212.96997 | 243.04335 | 409.142151 | 569.509521 | | 1.887186082 | | 1.330415964 | 1.309905497 | | 0.001328851 | 0.008634849 | 0.00904762 |
| **BMPR1B** | 252.579346 | | 170.37233 | 129.665482 | 3267.99829 | 6344.12939 | 4104.01953 | | -3.300153182 | | -5.191156172 | -4.778651532 | | 1.3129E-05 | 1.42227E-06 | 2.41673E-06 |
| **BMPR1B** | 115.227806 | | 134.404831 | 132.424316 | 776.492371 | 632.310608 | 583.166809 | | -2.399483488 | | -2.276547198 | -1.679326786 | | 0.000327792 | 0.000366001 | 0.004633406 |
| **BNC2** | 281.155853 | | 469.470428 | 410.147552 | 125.883995 | 105.924156 | 132.47583 | | 1.527700451 | | 2.064283337 | 2.15333826 | | 0.011742012 | 0.001122527 | 0.001036499 |
| **BTK** | 533.735168 | | 534.779785 | 1214.8092 | 314.086792 | 147.161804 | 385.135925 | | 1.195553796 | | 1.81133178 | 1.936322345 | | 0.021498792 | 0.002157525 | 0.000721119 |
| **BTN3A1** | 295.904999 | | 380.498199 | 392.674896 | 128.37674 | 71.9637375 | 98.3325806 | | 1.572317905 | | 2.307714945 | 2.529364708 | | 0.009599949 | 0.000780138 | 0.000423414 |
| **BTN3A2** | 2456.65674 | | 2138.17261 | 1948.66077 | 535.941772 | 764.109375 | 609.115723 | | 2.582275488 | | 1.554833999 | 1.82167166 | | 6.26691E-05 | 0.002466045 | 0.000877978 |
| **C10orf10** | 6018.57861 | | 5322.24219 | 5319.96338 | 555.883789 | 851.436157 | 2553.91553 | | 3.84231906 | | 2.760609268 | 0.893646852 | | 4.35829E-06 | 3.09637E-05 | 0.045346428 |
| **C11orf52** | 2966.42456 | | 2052.04004 | 2361.56714 | 7373.56152 | 4469.83789 | 5311.3252 | | -0.95663407 | | -1.027738788 | -1.320708799 | | 0.033315402 | 0.024171419 | 0.006203187 |
| **C16orf74** | 952.242554 | | 846.182556 | 3000.69849 | 321.565063 | 211.848312 | 260.854492 | | 1.970977306 | | 1.999902676 | 3.735971186 | | 0.000737062 | 0.000729453 | 6.23036E-06 |
| **C1orf186** | 1589.22192 | | 2169.40771 | 7299.89062 | 429.999786 | 212.656891 | 640.527527 | | 2.276462057 | | 3.341101865 | 3.511891556 | | 0.000190049 | 1.30681E-05 | 6.64751E-06 |
| **C2** | 1566.17627 | | 567.907776 | 4951.19824 | 209.39119 | 159.290527 | 234.905609 | | 3.285354882 | | 1.795777311 | 4.558760023 | | 1.75369E-05 | 0.002622187 | 2.28915E-06 |
| **C2orf70** | 8125.86475 | | 10641.6445 | 9109.69043 | 25124.4512 | 23132.7031 | 16567.6738 | | -1.225367802 | | -1.027515246 | -1.147834834 | | 0.008759497 | 0.026701508 | 0.012869423 |
| **C3** | 19526.043 | | 22140.8301 | 57467.5586 | 1025.76758 | 389.736206 | 835.826904 | | 4.652158523 | | 5.836953079 | 6.036560031 | | 1.7294E-06 | 9.65615E-07 | 8.73302E-07 |
| **C3AR1** | 725.474243 | | 1098.90149 | 1769.33606 | 100.956467 | 462.508545 | 128.378647 | | 3.186193528 | | 1.289712326 | 4.098981556 | | 4.49489E-05 | 0.009876051 | 5.48216E-06 |
| **C4A** | 11806.7021 | | 10815.8037 | 10796.2607 | 1533.04272 | 281.386322 | 3001.87524 | | 3.286588437 | | 5.328579288 | 1.631209345 | | 8.97488E-06 | 1.25269E-06 | 0.001382514 |
| **C4B** | 26650.8086 | | 17481.1465 | 19032.3184 | 2112.60767 | 228.019943 | 2982.75488 | | 4.031996969 | | 6.265150175 | 2.479114091 | | 2.94076E-06 | 8.75293E-07 | 5.73498E-05 |
| **C4B** | 19363.8027 | | 11912.8115 | 14643.9229 | 1557.97021 | 186.782288 | 2357.25049 | | 4.020281957 | | 6.017619925 | 2.448163155 | | 3.03724E-06 | 9.6977E-07 | 6.41302E-05 |
| **C5orf45** | 1690.62231 | | 2191.17749 | 2308.22949 | 949.738647 | 802.112671 | 1055.70947 | | 1.196784876 | | 1.520012416 | 1.173857194 | | 0.013060651 | 0.002843149 | 0.013797296 |
| **C7orf41** | 3295.51514 | | 3619.46558 | 1520.12085 | 28336.3613 | 25413.7109 | 17358.4316 | | -2.726466338 | | -2.755768707 | -3.616587668 | | 2.86832E-05 | 2.62284E-05 | 5.68973E-06 |
| **C9orf24** | 129.055145 | | 149.549042 | 173.806931 | 1213.97034 | 575.7099 | 749.78595 | | -2.881272493 | | -1.987180828 | -1.698868626 | | 6.15468E-05 | 0.000993009 | 0.003288029 |
| **CA2** | 953.164368 | | 816.840637 | 622.578247 | 2914.02734 | 6036.05957 | 1511.8634 | | -1.260325806 | | -2.765088084 | -1.102736039 | | 0.009146374 | 2.93274E-05 | 0.022434428 |
| **CACNA1H** | 592.731812 | | 593.463623 | 968.352844 | 3823.88208 | 1608.26831 | 2598.98462 | | -2.316752364 | | -1.379885267 | -1.380862605 | | 0.000128197 | 0.00569405 | 0.005542743 |
| **CAMK1D** | 748.519836 | | 1040.21777 | 1889.80542 | 342.753448 | 385.693298 | 506.685944 | | 1.542750908 | | 1.465687009 | 2.077444354 | | 0.004354877 | 0.004816808 | 0.000343735 |
| **CAPN12** | 2447.43848 | | 3414.07227 | 2303.63159 | 769.014099 | 192.442368 | 983.325806 | | 2.043148965 | | 4.150066565 | 1.282565726 | | 0.000341486 | 3.69406E-06 | 0.008396003 |
| **CAPS** | 176.989899 | | 259.344543 | 258.411346 | 1809.73816 | 923.399902 | 1083.02417 | | -2.95654823 | | -1.811046061 | -1.728395726 | | 3.66057E-05 | 0.001253229 | 0.0021089 |
| **CARS** | 671.086731 | | 723.135864 | 780.75177 | 153.30426 | 327.475433 | 191.20224 | | 2.484883679 | | 1.174129745 | 2.440515907 | | 0.00022098 | 0.019751834 | 0.000210753 |
| **CASZ1** | 123.524208 | | 125.886223 | 151.736206 | 1366.02832 | 682.442688 | 1305.63818 | | -3.128072912 | | -2.479378619 | -2.762185961 | | 3.34604E-05 | 0.000191757 | 8.05357E-05 |
| **CAV1** | 6397.44775 | | 11562.6016 | 9333.15625 | 2315.76685 | 2978.00513 | 3793.99878 | | 1.832519021 | | 2.065544813 | 1.060113858 | | 0.000613836 | 0.000233105 | 0.020080144 |
| **CAV2** | 2929.55176 | | 3770.90747 | 5824.83105 | 1250.11523 | 1123.9281 | 2003.52637 | | 1.579076317 | | 1.841593469 | 1.397547471 | | 0.002059143 | 0.000644609 | 0.004229687 |
| **CBLN3** | 534.656982 | | 303.830658 | 339.337341 | 120.898491 | 135.841675 | 198.030884 | | 2.498149639 | | 1.074583173 | 1.314283526 | | 0.00028706 | 0.049210481 | 0.019585082 |
| **CCDC34** | 1487.82141 | | 1818.25134 | 6139.33887 | 725.39093 | 1021.23822 | 930.062317 | | 1.413073033 | | 0.900091133 | 2.679128135 | | 0.005202343 | 0.048255321 | 3.74922E-05 |
| **CCDC8** | 673.852173 | | 495.026276 | 345.774628 | 3062.34619 | 2303.64819 | 2870.76514 | | -1.815878111 | | -2.178036081 | -2.865817023 | | 0.000822707 | 0.001111175 | 3.43445E-05 |
| **CCL11** | 80.198555 | | 103.169907 | 102.996696 | 635.651855 | 916.122681 | 385.135925 | | -2.630152455 | | -3.200089529 | -1.379183646 | | 0.000248378 | 2.89967E-05 | 0.020222012 |
| **CCL26** | 179.755371 | | 158.067657 | 233.581787 | 741.593811 | 483.531647 | 830.364014 | | -1.614299748 | | -1.667110834 | -1.448834037 | | 0.004226755 | 0.003573671 | 0.007534481 |
| **CCL4** | 1980.99646 | | 2382.37305 | 1415.28491 | 443.7099 | 367.90451 | 353.724152 | | 2.547736156 | | 2.71761821 | 2.271863847 | | 7.61645E-05 | 4.77822E-05 | 0.000210982 |
| **CCL5** | 24748.166 | | 15977.1387 | 45991.7031 | 11963.9648 | 4549.07861 | 8522.15723 | | 1.433794473 | | 1.925723477 | 2.111029117 | | 0.00322894 | 0.000388439 | 0.001689795 |
| **CCL5** | 7328.48828 | | 3821.07275 | 11013.29 | 2136.28882 | 1815.26514 | 1490.01172 | | 2.150484252 | | 1.158384895 | 2.755526153 | | 0.000180611 | 0.01329919 | 2.82414E-05 |
| **CCND1** | 44453.9648 | | 31774.4395 | 33905.2266 | 9245.61816 | 5863.02344 | 7141.40381 | | 2.661252485 | | 2.478714124 | 1.913797596 | | 3.29785E-05 | 5.57386E-05 | 0.000397473 |
| **CCR5** | 2520.26245 | | 2153.31689 | 3730.87134 | 550.898254 | 690.528503 | 682.865112 | | 2.578943505 | | 1.70854203 | 2.498771959 | | 6.25502E-05 | 0.001287727 | 6.93262E-05 |
| **CCRN4L** | 624.995605 | | 467.577393 | 800.063599 | 357.709961 | 142.310318 | 487.565704 | | 1.228200719 | | 1.650838701 | 1.012598938 | | 0.017161036 | 0.004328391 | 0.037882201 |
| **CD109** | 294.06134 | | 634.163696 | 350.372681 | 92.2318344 | 209.422577 | 154.327515 | | 2.040455934 | | 1.592799561 | 1.724334699 | | 0.002377209 | 0.004012441 | 0.004699043 |
| **CD247** | 1202.0564 | | 1065.77356 | 1675.53552 | 301.623016 | 157.67337 | 308.655029 | | 2.391592696 | | 2.733443942 | 2.708779779 | | 0.000161651 | 7.34776E-05 | 5.59932E-05 |
| **CD300A** | 492.253174 | | 460.00528 | 1648.8667 | 145.826004 | 114.81855 | 111.989883 | | 2.113543946 | | 1.917988119 | 4.205513166 | | 0.000914871 | 0.001808472 | 5.2001E-06 |
| **CD36** | 860.982178 | | 2556.53149 | 442.334015 | 326.550568 | 350.924316 | 202.128082 | | 1.808193641 | | 2.886519159 | 1.626562625 | | 0.00142454 | 2.9103E-05 | 0.005044467 |
| **CD40** | 1844.56665 | | 1732.11865 | 893.864197 | 587.043152 | 551.452454 | 462.982574 | | 2.035697146 | | 1.697040273 | 1.23956457 | | 0.000393622 | 0.001461971 | 0.013933059 |
| **CD52** | 2901.89697 | | 5002.3208 | 4861.07617 | 805.158997 | 648.482239 | 1842.37012 | | 2.220460063 | | 3.053650561 | 1.282672848 | | 0.000174368 | 1.62105E-05 | 0.007382586 |
| **CD72** | 605.637329 | | 525.314697 | 2946.44116 | 413.796875 | 259.554626 | 289.534821 | | 0.97152048 | | 1.01177901 | 3.552124424 | | 0.048941005 | 0.044069875 | 8.11043E-06 |
| **CD82** | 495.018646 | | 366.300507 | 478.198944 | 1316.17322 | 2890.67822 | 1108.97302 | | -1.022653989 | | -2.95455019 | -0.953187045 | | 0.044512682 | 2.28969E-05 | 0.048645243 |
| **CD86** | 624.073792 | | 684.328857 | 1490.69324 | 154.550644 | 192.442368 | 195.299438 | | 2.372710797 | | 1.824695156 | 3.256918381 | | 0.000324483 | 0.001610045 | 1.92632E-05 |
| **CD93** | 7701.82617 | | 7772.76416 | 18960.5879 | 4883.30176 | 4671.98291 | 8171.16406 | | 1.022132749 | | 0.887809643 | 0.934963306 | | 0.024039783 | 0.044978485 | 0.035430256 |
| **CDCA5** | 341.996124 | | 330.333008 | 1217.56812 | 219.362198 | 150.396133 | 167.984833 | | 1.078503919 | | 1.059247399 | 3.214262934 | | 0.044771452 | 0.048880108 | 2.44724E-05 |
| **CDCA7L** | 2245.55933 | | 2141.95874 | 1513.68359 | 678.028625 | 734.191895 | 420.644928 | | 2.1056983 | | 1.615231645 | 2.084253656 | | 0.000282753 | 0.001901907 | 0.00037557 |
| **CDH13** | 1213.11829 | | 1652.61157 | 998.700134 | 316.579529 | 718.828796 | 232.174149 | | 2.334490407 | | 1.27170134 | 2.466107513 | | 0.000189805 | 0.009302081 | 0.000155066 |
| **CDH16** | 3179.36548 | | 5155.65576 | 7654.86182 | 41708.7305 | 18005.4902 | 28715.8457 | | -3.344604905 | | -1.692106253 | -2.232678788 | | 7.56748E-06 | 0.001014046 | 0.000124107 |
| **CDH2** | 5666.44238 | | 4595.32031 | 4972.34961 | 1034.49219 | 768.960876 | 848.11853 | | 2.829394294 | | 2.690243393 | 2.533948464 | | 2.59019E-05 | 0.000527655 | 0.000416969 |
| **CDH5** | 7093.42383 | | 5770.88916 | 8105.47217 | 2343.18726 | 2432.21265 | 1973.48022 | | 1.972558355 | | 1.354819061 | 1.880829612 | | 0.000348513 | 0.005054134 | 0.000497828 |
| **CDS1** | 459.98938 | | 449.593658 | 210.591461 | 1650.20203 | 1753.81299 | 905.479187 | | -1.457236366 | | -1.933230932 | -1.725045725 | | 0.004627823 | 0.000619541 | 0.002493893 |
| **CEBPB** | 6190.95947 | | 5178.37207 | 26892.2539 | 1317.41956 | 2805.77734 | 2761.50659 | | 2.603656178 | | 0.98873629 | 3.066408249 | | 4.52477E-05 | 0.028718146 | 1.29467E-05 |
| **CECR1** | 1551.42712 | | 990.052551 | 1861.29749 | 852.521301 | 329.901184 | 614.578613 | | 1.233817847 | | 1.615268276 | 1.747455432 | | 0.011336702 | 0.002687377 | 0.001201003 |
| **CENPE** | 292.217712 | | 267.863159 | 670.398132 | 123.391243 | 107.541321 | 192.567963 | | 1.611587709 | | 1.209723932 | 2.235167876 | | 0.008567276 | 0.034606203 | 0.000443094 |
| **CEP250** | 742.067078 | | 681.489319 | 878.230774 | 458.666412 | 249.04306 | 423.376404 | | 1.105835667 | | 1.467541399 | 1.358425671 | | 0.025934035 | 0.006148336 | 0.008525917 |
| **CFB** | 2397.66016 | | 1484.13232 | 2786.42847 | 472.376556 | 614.521851 | 372.84436 | | 2.731549456 | | 1.331827621 | 3.079369084 | | 4.30866E-05 | 0.007337904 | 2.08314E-05 |
| **CGNL1** | 2817.08936 | | 2179.81934 | 1700.36511 | 10216.5449 | 6110.44922 | 7997.7168 | | -1.494693657 | | -1.378215314 | -2.36329638 | | 0.002659336 | 0.004495122 | 9.24713E-05 |
| **CHST11** | 375.181732 | | 509.223969 | 774.314453 | 165.768036 | 185.165131 | 152.961792 | | 1.565955791 | | 1.423487955 | 2.758499159 | | 0.007402991 | 0.009139379 | 9.68049E-05 |
| **CHST11** | 321.716034 | | 318.974854 | 990.423584 | 109.681099 | 143.118896 | 142.03595 | | 1.918595789 | | 1.075211141 | 3.189282363 | | 0.002929898 | 0.047408 | 3.11457E-05 |
| **CHST13** | 1213.11829 | | 1073.3457 | 1977.1687 | 301.623016 | 91.3696899 | 342.798309 | | 2.404751338 | | 3.503660184 | 2.75812236 | | 0.000154596 | 1.74355E-05 | 4.51086E-05 |
| **CHST15** | 1108.95239 | | 1806.89319 | 1792.32642 | 549.651855 | 651.716553 | 659.647705 | | 1.401683939 | | 1.532641304 | 1.584470793 | | 0.006173191 | 0.00285983 | 0.002387766 |
| **CKB** | 5795.49756 | | 5833.35938 | 4577.83545 | 37336.4453 | 12341.377 | 22392.5137 | | -2.297833043 | | -0.930131655 | -2.542870448 | | 9.86302E-05 | 0.036388766 | 4.78575E-05 |
| **CKB** | 446.162048 | | 485.561127 | 285.080139 | 3249.30273 | 1845.18262 | 1729.01453 | | -2.479962926 | | -1.888741983 | -2.332980042 | | 8.36607E-05 | 0.000649152 | 0.000190982 |
| **CLDN10** | 1547.73987 | | 1498.32996 | 323.703918 | 7076.92383 | 9583.30664 | 3354.23364 | | -1.837594811 | | -2.563922849 | -3.195418655 | | 0.000617106 | 4.68102E-05 | 1.61809E-05 |
| **CLDN14** | 449.849335 | | 251.772446 | 768.796753 | 3195.7085 | 2597.16333 | 6679.78662 | | -2.444363687 | | -3.358917299 | -3.134667181 | | 0.000172187 | 1.09304E-05 | 1.40086E-05 |
| **CLIC6** | 2016.02563 | | 771.40802 | 641.890137 | 169.507156 | 285.42923 | 292.266296 | | 3.916819011 | | 1.461049099 | 1.541468248 | | 6.09585E-06 | 0.00580484 | 0.004993694 |
| **CMC4** | 2911.11523 | | 3064.80884 | 5959.09473 | 1354.81091 | 1157.07996 | 940.988159 | | 1.450069672 | | 1.483175266 | 2.619352813 | | 0.00364649 | 0.003083619 | 4.42121E-05 |
| **CMTM4** | 209.253693 | | 191.195618 | 78.1671371 | 651.854736 | 1254.10962 | 329.140991 | | -1.205261553 | | -2.721303428 | -1.527485023 | | 0.02284685 | 6.01847E-05 | 0.015408164 |
| **CMTM7** | 840.702026 | | 932.315247 | 2153.73438 | 377.651978 | 223.977036 | 676.036499 | | 1.563558674 | | 2.06349133 | 1.785599594 | | 0.003694987 | 0.000544009 | 0.000975012 |
| **CNDP2** | 1781.88281 | | 2408.87549 | 1710.48083 | 730.376404 | 913.696899 | 741.591553 | | 1.662003758 | | 1.46858398 | 1.335340085 | | 0.001685772 | 0.003474912 | 0.007173762 |
| **COBLL1** | 2646.55225 | | 2453.36157 | 1737.14966 | 10054.5166 | 13670.6846 | 12602.959 | | -1.56022689 | | -2.386240296 | -3.003817726 | | 0.001927928 | 7.68E-05 | 1.61628E-05 |
| **COL14A1** | 1988.37097 | | 1120.67126 | 2040.62207 | 12304.2256 | 14020.8008 | 9095.76367 | | -2.281891479 | | -3.546185149 | -2.315312095 | | 0.000112203 | 5.69585E-06 | 0.000105272 |
| **COL1A1** | 4240.3833 | | 4259.30811 | 7524.27637 | 1236.40515 | 534.47229 | 2422.80542 | | 2.126606555 | | 3.075333403 | 1.458056394 | | 0.000216556 | 1.61864E-05 | 0.003113035 |
| **COL23A1** | 5871.08691 | | 4352.06641 | 525.099243 | 144.579636 | 72.7723236 | 140.670227 | | 5.679235108 | | 5.879438446 | 2.385180787 | | 1.21522E-06 | 1.24731E-06 | 0.000392014 |
| **COL4A1** | 6199.25586 | | 7468.93359 | 14831.5244 | 1869.56421 | 3176.10767 | 2160.58521 | | 2.094656104 | | 1.357998286 | 2.603033459 | | 0.000225894 | 0.004867137 | 4.09795E-05 |
| **COL4A2** | 5115.19287 | | 3910.99146 | 6290.15527 | 2492.7522 | 1369.73682 | 1492.74316 | | 1.385583576 | | 1.606872876 | 1.968645632 | | 0.004545129 | 0.001705361 | 0.00184636 |
| **COL4A4** | 225.846497 | | 211.072388 | 92.8809509 | 668.057617 | 1238.74658 | 879.530273 | | -1.13226205 | | -2.55238644 | -2.845801758 | | 0.028838979 | 9.34138E-05 | 0.000109011 |
| **COPS6** | 859.138489 | | 874.577942 | 371.523804 | 2108.86841 | 1955.14966 | 1158.13928 | | -0.937823549 | | -1.091892342 | -1.348402793 | | 0.042124186 | 0.01998844 | 0.008950862 |
| **COPZ1** | 2068.56958 | | 1810.6792 | 3902.83911 | 1413.3905 | 812.624268 | 1251.00891 | | 0.894514397 | | 1.224927543 | 1.604212517 | | 0.048965935 | 0.011163683 | 0.001773778 |
| **CORO1A** | 815.812866 | | 856.594238 | 1349.99243 | 260.492615 | 240.148666 | 240.36853 | | 2.061184403 | | 1.846892605 | 2.809829087 | | 0.000617367 | 0.001230738 | 5.10186E-05 |
| **CPAMD8** | 119.836914 | | 106.955963 | 124.147804 | 1114.26025 | 802.921265 | 763.443237 | | -2.873259534 | | -2.957107985 | -2.204981429 | | 6.86691E-05 | 5.27235E-05 | 0.000637911 |
| **CRABP2** | 91.2604218 | | 110.742012 | 102.996696 | 828.840149 | 232.871429 | 409.719086 | | -2.837133018 | | -1.192399619 | -1.477509233 | | 0.00010713 | 0.037649248 | 0.013711752 |
| **CRHBP** | 485.800415 | | 466.63089 | 532.456116 | 1805.99902 | 983.234924 | 1683.94543 | | -1.511357438 | | -1.029290634 | -1.473028241 | | 0.003535522 | 0.031356182 | 0.004361278 |
| **CRLF1** | 608.402832 | | 510.170471 | 289.678223 | 1976.75256 | 1216.91492 | 1759.06055 | | -1.326527594 | | -1.202617727 | -2.338515594 | | 0.007626139 | 0.013694328 | 0.000185487 |
| **CSDA** | 2182.87549 | | 1452.89734 | 2760.6792 | 497.304077 | 713.977356 | 704.716797 | | 2.521164243 | | 1.09898033 | 2.048826286 | | 7.81203E-05 | 0.020814406 | 0.000328068 |
| **CSF1R** | 1482.29041 | | 2161.83545 | 5886.44531 | 837.564758 | 682.442688 | 717.008423 | | 1.194572 | | 1.730166659 | 3.031211551 | | 0.013686639 | 0.00117675 | 1.6533E-05 |
| **CSNK1D** | 2381.06738 | | 2309.49146 | 4511.62305 | 1455.76733 | 1054.39014 | 1802.76392 | | 1.053657989 | | 1.200179624 | 1.218430094 | | 0.02332362 | 0.011837983 | 0.010071667 |
| **CTDSPL** | 3019.89038 | | 2944.60181 | 1666.33936 | 8697.21289 | 7245.69727 | 4961.69824 | | -1.155955011 | | -1.177179278 | -1.677117079 | | 0.012839179 | 0.039687723 | 0.001281591 |
| **CTHRC1** | 783.549072 | | 993.838562 | 2855.39941 | 98.4637146 | 117.244293 | 152.961792 | | 3.329618491 | | 3.036303379 | 4.473224594 | | 3.21035E-05 | 4.13032E-05 | 2.98296E-06 |
| **CTNNAL1** | 271.937622 | | 263.130585 | 142.54007 | 755.303955 | 1564.60486 | 807.146606 | | -1.047973144 | | -2.559099907 | -2.095651859 | | 0.037451863 | 7.91912E-05 | 0.000821731 |
| **CTSB** | 10714.3418 | | 8329.31348 | 13438.3096 | 7392.25684 | 3268.28589 | 5910.88086 | | 0.88973858 | | 1.474711658 | 0.896024662 | | 0.044200983 | 0.002808456 | 0.046469063 |
| **CXCL12** | 1980.07458 | | 1044.95032 | 1498.96973 | 13665.2686 | 12695.5361 | 6245.48438 | | -2.448686276 | | -3.494120437 | -2.159041319 | | 6.56338E-05 | 6.22438E-06 | 0.000190156 |
| **CXCL12** | 931.040649 | | 224.323563 | 242.777924 | 3095.99829 | 2961.8335 | 1211.40271 | | -1.380514221 | | -3.720581657 | -1.988611396 | | 0.005228123 | 6.04883E-06 | 0.000762052 |
| **CXCL16** | 738.379761 | | 748.691711 | 1519.20129 | 380.144714 | 305.643738 | 397.427521 | | 1.37303102 | | 1.321467843 | 2.179226271 | | 0.008636676 | 0.010420291 | 0.000272289 |
| **CXCR4** | 4421.98242 | | 5181.21191 | 3483.49536 | 848.782166 | 543.366699 | 621.407288 | | 2.747173424 | | 3.344612811 | 2.559635505 | | 3.35121E-05 | 9.31985E-06 | 5.95878E-05 |
| **CYB5B** | 251.657516 | | 297.205048 | 235.421021 | 1147.91248 | 649.290833 | 807.146606 | | -1.781648061 | | -1.106500956 | -1.393741767 | | 0.001577537 | 0.026528042 | 0.009525788 |
| **CYBA** | 18857.7207 | | 14284.7734 | 21147.4297 | 5844.25781 | 4538.56689 | 4058.95044 | | 2.070858203 | | 1.777438331 | 2.153878608 | | 0.000221553 | 0.000832189 | 0.000164152 |
| **CYFIP2** | 4692.99805 | | 5891.09668 | 5722.75391 | 26254.9141 | 24094.916 | 15843.8369 | | -2.102622134 | | -1.943522302 | -1.73504722 | | 0.000559351 | 0.001638114 | 0.00086551 |
| **CYP3A5** | 2722.1416 | | 2066.23779 | 7440.5918 | 577.072144 | 84.9010391 | 508.051666 | | 2.62217415 | | 4.543607314 | 3.910722509 | | 5.39974E-05 | 3.39835E-06 | 3.87139E-06 |
| **CYS1** | 457.223938 | | 276.381775 | 73.5690689 | 1870.81067 | 1949.48962 | 1058.44092 | | -1.647499492 | | -2.804791712 | -3.472388483 | | 0.001977703 | 3.77796E-05 | 2.85964E-05 |
| **DBN1** | 330.934265 | | 379.551697 | 359.568817 | 1366.02832 | 858.713379 | 931.42804 | | -1.649792955 | | -1.141823386 | -1.049251349 | | 0.002320644 | 0.020261748 | 0.034360249 |
| **DCDC2** | 109.696869 | | 136.297867 | 88.2828827 | 418.782379 | 342.838501 | 696.522461 | | -1.566770471 | | -1.411859081 | -2.549446105 | | 0.009828647 | 0.012432024 | 0.000295747 |
| **DCN** | 117.993271 | | 111.68853 | 122.308578 | 2034.08594 | 3510.86035 | 1723.55164 | | -3.781336699 | | -5.015270676 | -3.507742202 | | 8.71422E-06 | 1.81884E-06 | 1.55501E-05 |
| **DCTPP1** | 1074.84497 | | 1042.11072 | 853.401184 | 3891.18628 | 2179.12671 | 2675.46558 | | -1.510707097 | | -0.995849325 | -1.592497113 | | 0.00277618 | 0.030426033 | 0.002173126 |
| **DDB2** | 2288.88501 | | 2544.22681 | 1512.76404 | 469.88382 | 670.313965 | 528.537598 | | 2.672337251 | | 1.990256202 | 1.719138323 | | 5.1074E-05 | 0.000405657 | 0.00146961 |
| **DENND1C** | 402.836395 | | 507.330933 | 350.372681 | 236.811462 | 68.7294159 | 237.63707 | | 1.208451075 | | 2.807362572 | 1.076823076 | | 0.024814952 | 0.00015558 | 0.044636056 |
| **DESI1** | 3407.05566 | | 3082.79272 | 4979.70654 | 2234.75244 | 1555.71057 | 1506.40051 | | 0.94816234 | | 1.05744627 | 1.632656938 | | 0.036131293 | 0.021895049 | 0.001501975 |
| **DGKD** | 528.204224 | | 869.845398 | 541.652283 | 120.898491 | 111.584229 | 223.979767 | | 2.481113911 | | 2.913154236 | 1.731478622 | | 0.000304522 | 6.08368E-05 | 0.002853848 |
| **DIAPH2** | 2082.39697 | | 2064.34473 | 2946.44116 | 488.579468 | 317.772461 | 469.811218 | | 2.479066371 | | 2.716951536 | 2.78623216 | | 8.99397E-05 | 4.80623E-05 | 3.51087E-05 |
| **DIAPH2** | 480.26947 | | 707.045166 | 777.99292 | 97.2173386 | 224.785614 | 258.123016 | | 2.66215003 | | 1.661051168 | 1.977444459 | | 0.000228459 | 0.002840465 | 0.000868607 |
| **DNAJC11** | 2756.24902 | | 3159.46021 | 3599.3667 | 10829.7627 | 6433.88184 | 6756.26758 | | -1.615492217 | | -0.902437956 | -1.120719303 | | 0.001497714 | 0.042416606 | 0.015440357 |
| **DNAJC11** | 926.431519 | | 976.801331 | 1538.51318 | 3852.54858 | 2171.84961 | 2914.46851 | | -1.703393573 | | -1.084067783 | -0.951092081 | | 0.001212762 | 0.020328708 | 0.037480515 |
| **DOC2A** | 1199.29102 | | 2035.94934 | 3306.01001 | 130.869492 | 104.306992 | 136.573029 | | 3.515341943 | | 4.225239339 | 4.834840252 | | 1.52404E-05 | 4.4623E-06 | 2.17183E-06 |
| **DOCK8** | 474.738556 | | 713.670776 | 469.002808 | 77.275322 | 305.643738 | 113.355614 | | 2.977104833 | | 1.252610745 | 2.554507441 | | 0.000122616 | 0.014201237 | 0.000301417 |
| **DOK4** | 554.015259 | | 1230.4668 | 555.446472 | 291.652008 | 321.006805 | 312.752228 | | 1.355462764 | | 1.963939313 | 1.251605785 | | 0.011213619 | 0.000627621 | 0.018793458 |
| **DOK7** | 146.569763 | | 235.681717 | 201.395325 | 1991.70911 | 679.208313 | 1556.9325 | | -3.400241188 | | -1.516945064 | -2.640289385 | | 1.51044E-05 | 0.004828545 | 9.05465E-05 |
| **DPYS** | 2348.80347 | | 2406.98242 | 3368.5437 | 1311.18774 | 118.861458 | 894.553345 | | 1.189618082 | | 4.283353814 | 1.935801005 | | 0.012509587 | 1.98881E-05 | 0.000471154 |
| **DTX2** | 3329.62256 | | 3402.71411 | 7663.13818 | 1929.39026 | 1637.3772 | 2258.91797 | | 1.128032422 | | 1.132744185 | 1.592505926 | | 0.015713697 | 0.015214892 | 0.001694331 |
| **DUSP15** | 438.787476 | | 128.725754 | 485.555847 | 4796.05566 | 1410.97449 | 3948.32617 | | -3.067383408 | | -3.497373601 | -2.923676872 | | 1.7351E-05 | 1.23252E-05 | 2.58691E-05 |
| **DYRK3** | 1174.40173 | | 1538.0835 | 1464.02441 | 479.854828 | 656.568054 | 688.328064 | | 1.681796071 | | 1.293054704 | 1.252002978 | | 0.00194564 | 0.008610172 | 0.012261042 |
| **EDN1** | 6945.01025 | | 6167.47852 | 5700.68311 | 262.985382 | 1233.89514 | 1520.05786 | | 5.150431825 | | 2.440302264 | 1.803069737 | | 1.46198E-06 | 7.20739E-05 | 0.000715636 |
| **EEF1B2** | 949.477112 | | 889.722168 | 1504.48743 | 3406.34595 | 2198.53271 | 3183.51733 | | -1.4910917 | | -1.235828364 | -1.11800093 | | 0.003117558 | 0.010080462 | 0.0172513 |
| **EFHD1** | 207.410049 | | 339.798157 | 377.96109 | 1896.9845 | 1172.44299 | 1139.01904 | | -2.796401957 | | -1.756511662 | -1.300034879 | | 5.01941E-05 | 0.001337708 | 0.011033208 |
| **EFNA1** | 13484.4189 | | 12985.2109 | 30273.6719 | 4999.21484 | 5246.07568 | 4076.70483 | | 1.782618074 | | 1.445297519 | 2.61127214 | | 0.000703098 | 0.003128724 | 3.83865E-05 |
| **EFNB2** | 890.480469 | | 1145.28064 | 840.526611 | 467.391052 | 398.630615 | 415.182007 | | 1.332496671 | | 1.556462568 | 1.331667737 | | 0.009199688 | 0.003138464 | 0.009753303 |
| **EHD2** | 2729.51611 | | 2977.72974 | 3397.05176 | 1086.83997 | 623.416199 | 1119.8988 | | 1.686048568 | | 2.322156048 | 1.594697615 | | 0.001328163 | 0.000123955 | 0.001902971 |
| **EIF4EBP1** | 3957.38379 | | 5235.16309 | 4334.1377 | 2128.81055 | 1220.14929 | 1283.7865 | | 1.233570047 | | 2.213575124 | 1.701854501 | | 0.009369071 | 0.000152864 | 0.001143415 |
| **ELF4** | 882.184082 | | 1162.31787 | 1501.72864 | 233.072342 | 358.201538 | 517.611755 | | 2.331698177 | | 1.728388993 | 1.743141157 | | 0.000249244 | 0.001563961 | 0.001339777 |
| **ELOVL5** | 341.07431 | | 340.744659 | 529.697266 | 204.405685 | 155.24762 | 290.900543 | | 1.164257842 | | 1.061750246 | 1.302966009 | | 0.033139769 | 0.047423124 | 0.014447446 |
| **EMB** | 425.881958 | | 450.540161 | 493.832367 | 250.521606 | 145.544647 | 225.34549 | | 1.206042674 | | 1.563273553 | 1.602966884 | | 0.024018984 | 0.006127048 | 0.004934859 |
| **EMX1** | 286.686768 | | 336.012085 | 502.108887 | 1336.11523 | 1008.30096 | 1752.23193 | | -1.821364362 | | -1.553880915 | -1.611606717 | | 0.001219665 | 0.003275037 | 0.00241092 |
| **ENDOG** | 417.585571 | | 488.400665 | 273.125183 | 2546.34644 | 1736.83276 | 1051.6123 | | -2.222377121 | | -1.791865773 | -1.607190768 | | 0.000205168 | 0.000969819 | 0.003405784 |
| **ENPEP** | 378.869019 | | 768.568481 | 325.543121 | 178.231796 | 76.8152313 | 211.688202 | | 1.487912376 | | 3.27389926 | 1.157614766 | | 0.009594492 | 3.63846E-05 | 0.035232782 |
| **ENTPD1** | 508.845978 | | 612.39386 | 1447.47144 | 282.927399 | 254.703125 | 294.997742 | | 1.280234942 | | 1.272875197 | 2.587503937 | | 0.015950412 | 0.014365234 | 8.3879E-05 |
| **EPB41L5** | 1658.35852 | | 2919.99243 | 1271.82532 | 503.53598 | 377.607483 | 468.445496 | | 2.107146886 | | 2.980173673 | 1.685572967 | | 0.000323182 | 2.24286E-05 | 0.001829188 |
| **EPHA7** | 2234.49756 | | 1574.05103 | 2424.10083 | 877.448792 | 522.343567 | 549.02356 | | 1.716373024 | | 1.634662702 | 2.277119423 | | 0.004352115 | 0.001962752 | 0.0001787 |
| **ERAP2** | 2680.65967 | | 2558.42456 | 4047.21851 | 595.767822 | 288.663544 | 767.540405 | | 2.552770565 | | 3.163369401 | 2.418517056 | | 6.56703E-05 | 1.74138E-05 | 8.58609E-05 |
| **ERCC5** | 3633.82397 | | 4146.67334 | 6246.01416 | 2167.44824 | 1872.67444 | 2451.48584 | | 1.085010078 | | 1.238213897 | 1.180655802 | | 0.019004179 | 0.009050679 | 0.011635249 |
| **ERGIC1** | 7186.52783 | | 11119.6338 | 13912.8301 | 4216.49072 | 3813.26978 | 4448.18359 | | 1.141215121 | | 1.675949657 | 1.379437485 | | 0.013693845 | 0.001118758 | 0.004290867 |
| **ERMP1** | 783.549072 | | 928.529175 | 445.092865 | 3382.66479 | 7873.96533 | 2170.14551 | | -1.748792342 | | -2.958992372 | -2.10308071 | | 0.001043129 | 1.78688E-05 | 0.000342491 |
| **ESM1** | 2113.73877 | | 3368.63965 | 583.954468 | 162.0289 | 96.2211838 | 222.614044 | | 4.042306573 | | 5.086266791 | 1.836999219 | | 5.16693E-06 | 1.88204E-06 | 0.001834654 |
| **ESRP2** | 181.599014 | | 211.072388 | 163.691177 | 675.535889 | 760.875061 | 760.711792 | | -1.461369865 | | -1.844780118 | -1.806835145 | | 0.008101876 | 0.00127184 | 0.002227616 |
| **ETS1** | 2367.23999 | | 1918.58167 | 1478.73828 | 257.999878 | 789.175415 | 397.427521 | | 3.593366726 | | 1.351156117 | 2.143989381 | | 8.39901E-06 | 0.00625196 | 0.000314938 |
| **EVL** | 3815.4231 | | 2079.48901 | 5450.54834 | 1496.89771 | 954.125977 | 1518.69214 | | 1.691372655 | | 1.192943232 | 1.742053927 | | 0.00120131 | 0.012503851 | 0.000928153 |
| **F2RL3** | 2776.52905 | | 738.28009 | 4953.95703 | 178.231796 | 75.1980667 | 617.310059 | | 4.314594621 | | 3.246805412 | 3.035602053 | | 3.43998E-06 | 4.00601E-05 | 1.70432E-05 |
| **FABP6** | 3484.48877 | | 2723.11768 | 2482.03638 | 104.695595 | 80.0495529 | 131.110107 | | 5.37173904 | | 5.028279048 | 4.513956448 | | 1.60928E-06 | 2.1373E-06 | 3.09601E-06 |
| **FADS3** | 2812.48022 | | 2717.43872 | 5638.14941 | 1372.26013 | 1095.62769 | 1683.94543 | | 1.381467206 | | 1.3801991 | 1.625932087 | | 0.005007084 | 0.005025899 | 0.001517936 |
| **FAM101B** | 1342.17346 | | 1708.45581 | 1728.87317 | 342.753448 | 240.148666 | 558.583679 | | 2.364386976 | | 2.830200758 | 1.804603859 | | 0.000161712 | 4.09666E-05 | 0.00099221 |
| **FAM101B** | 851.763916 | | 760.996399 | 1199.17578 | 483.593964 | 128.564438 | 525.806152 | | 1.220772562 | | 2.527137182 | 1.423112049 | | 0.015045567 | 0.000171205 | 0.005582636 |
| **FAM108C1** | 769.721741 | | 538.565857 | 1041.922 | 3618.22998 | 3801.14087 | 3890.96558 | | -1.870802774 | | -2.746434877 | -1.914241914 | | 0.001217972 | 3.39613E-05 | 0.00052441 |
| **FAM129A** | 2221.59204 | | 2431.5918 | 5375.14014 | 1392.20215 | 1123.11951 | 2086.83594 | | 1.019931161 | | 1.183350713 | 1.222342721 | | 0.027530281 | 0.012666722 | 0.034719203 |
| **FAM156A** | 1162.41809 | | 1257.91565 | 2383.63794 | 347.738953 | 494.851776 | 1091.21851 | | 2.136876146 | | 1.389953981 | 1.164717503 | | 0.000363342 | 0.006061677 | 0.014295573 |
| **FAM171A1** | 791.845459 | | 615.233398 | 651.086243 | 3208.17212 | 3781.73486 | 3955.15479 | | -1.657648893 | | -2.535175879 | -2.549939923 | | 0.001545431 | 5.95879E-05 | 6.4861E-05 |
| **FAM183A** | 817.656494 | | 944.619934 | 461.645905 | 284.173767 | 142.310318 | 218.516846 | | 1.938010313 | | 2.699547327 | 1.562678653 | | 0.000933869 | 8.9279E-05 | 0.006077799 |
| **FBLN1** | 674.774048 | | 655.933472 | 1709.56128 | 13531.9062 | 9225.91309 | 9047.96289 | | -3.959444166 | | -3.694389117 | -2.538583696 | | 3.47027E-06 | 4.8123E-06 | 5.37313E-05 |
| **FBXO17** | 871.122192 | | 889.722168 | 747.645691 | 382.637482 | 213.465485 | 285.437622 | | 1.593964113 | | 2.061674657 | 1.772669049 | | 0.003552172 | 0.000568979 | 0.001943514 |
| **FBXO2** | 827.79657 | | 459.058777 | 381.639557 | 4995.47559 | 4883.02295 | 1989.86902 | | -2.23684535 | | -3.335987059 | -2.167489683 | | 0.000152927 | 9.32556E-06 | 0.000289796 |
| **FCER1G** | 368.728973 | | 591.570557 | 584.874084 | 225.594086 | 236.914337 | 136.573029 | | 1.150241059 | | 1.31827347 | 2.566569545 | | 0.032770652 | 0.012245548 | 0.000214466 |
| **FCGR1B** | 1341.25159 | | 1183.14111 | 5504.80566 | 98.4637146 | 567.624084 | 176.179199 | | 4.086507816 | | 1.117821738 | 5.137200918 | | 6.88632E-06 | 0.02032421 | 1.55322E-06 |
| **FCGR2C** | 458.145752 | | 460.951813 | 1903.59961 | 95.9709625 | 162.524857 | 173.447754 | | 2.614600759 | | 1.448125387 | 3.751399948 | | 0.000273781 | 0.00910271 | 7.74891E-06 |
| **FDXR** | 595.497314 | | 562.228699 | 381.639557 | 376.405609 | 163.333435 | 233.539886 | | 1.086102158 | | 1.74623158 | 1.214238286 | | 0.031218356 | 0.002562714 | 0.025535408 |
| **FECH** | 278.390381 | | 348.316772 | 184.842285 | 1372.26013 | 2590.69458 | 625.504456 | | -1.903136555 | | -2.872623551 | -1.320727511 | | 0.000887895 | 2.84546E-05 | 0.015455183 |
| **FECH** | 114.305984 | | 220.537521 | 101.157471 | 444.956299 | 1390.75989 | 494.394348 | | -1.592789267 | | -2.655440722 | -1.802357323 | | 0.008478867 | 6.60524E-05 | 0.003833035 |
| **FECH** | 164.084396 | | 160.907196 | 114.951668 | 1212.724 | 1086.7334 | 397.427521 | | -2.491536361 | | -2.77471159 | -1.270677469 | | 0.00015634 | 5.91384E-05 | 0.027930137 |
| **FEM1C** | 424.03833 | | 843.343018 | 1133.8833 | 133.362244 | 283.003479 | 269.048859 | | 2.030197631 | | 1.601091306 | 2.407847701 | | 0.001803984 | 0.003139129 | 0.000164198 |
| **FGF1** | 220.315567 | | 134.404831 | 256.572113 | 12579.6748 | 693.762817 | 3829.50781 | | -5.435915568 | | -2.401800709 | -3.717692011 | | 1.21854E-06 | 0.000232539 | 6.72085E-06 |
| **FKBP10** | 894.167786 | | 911.491943 | 1608.40381 | 517.246094 | 214.274063 | 262.220215 | | 1.190286141 | | 2.0912768 | 2.905315718 | | 0.016766012 | 0.000506445 | 3.63981E-05 |
| **FKBP11** | 2723.98535 | | 2010.39343 | 1511.84436 | 616.956177 | 647.673645 | 714.276917 | | 2.52408042 | | 1.695602579 | 1.234503429 | | 0.000173911 | 0.001389819 | 0.011676951 |
| **FKBP5** | 1108.03052 | | 1243.71802 | 2918.85278 | 257.999878 | 631.502014 | 198.030884 | | 2.501948513 | | 1.044569583 | 4.120782008 | | 0.000126703 | 0.027616849 | 4.04886E-06 |
| **FLT1** | 3879.95068 | | 3993.33813 | 2154.65405 | 1018.28931 | 1279.98425 | 1115.80164 | | 2.28847288 | | 1.738118037 | 0.995553906 | | 0.000128166 | 0.000971465 | 0.031530097 |
| **FLT1** | 7501.79102 | | 8307.54395 | 6991.82031 | 802.66626 | 824.752991 | 969.668518 | | 3.624187774 | | 3.456989851 | 2.79467478 | | 5.56959E-06 | 7.05236E-06 | 2.73387E-05 |
| **FOXC1** | 1375.35901 | | 453.3797 | 1590.93115 | 7591.67725 | 6828.46973 | 3961.98364 | | -2.10288944 | | -3.82511423 | -1.387210065 | | 0.000222034 | 4.2448E-06 | 0.004807721 |
| **FOXI1** | 119.836914 | | 125.886223 | 114.032059 | 820.11554 | 697.805725 | 543.560669 | | -2.417105895 | | -2.509442899 | -1.78183953 | | 0.00029205 | 0.000181294 | 0.003606098 |
| **FPR3** | 1637.15662 | | 1596.76733 | 2310.06885 | 215.623077 | 361.435852 | 289.534821 | | 3.311812751 | | 2.165739542 | 3.228727599 | | 1.64733E-05 | 0.00026944 | 1.5432E-05 |
| **FRAS1** | 909.838745 | | 539.51239 | 1013.41394 | 2313.27417 | 2217.13013 | 2781.99268 | | -0.991875714 | | -1.990932381 | -1.426835382 | | 0.032534026 | 0.000412972 | 0.004394448 |
| **FREM2** | 219.393738 | | 242.307312 | 75.4082947 | 620.695312 | 639.58783 | 424.742126 | | -1.064811858 | | -1.393500007 | -1.984536111 | | 0.039069951 | 0.008234975 | 0.002805823 |
| **FSTL3** | 2302.7124 | | 3174.60449 | 3477.05811 | 776.492371 | 234.488586 | 1068.0011 | | 1.940923919 | | 3.770435493 | 1.699850717 | | 0.000508395 | 8.54656E-06 | 0.001206811 |
| **FXYD5** | 2805.10571 | | 2299.07983 | 4648.64551 | 894.898071 | 1083.49902 | 1439.47974 | | 2.015066292 | | 1.154201302 | 1.613552387 | | 0.000361137 | 0.014638661 | 0.001650517 |
| **GABRD** | 3489.0979 | | 2525.29663 | 7277.82031 | 621.941711 | 146.353226 | 524.44043 | | 2.867852684 | | 4.069611094 | 3.828936075 | | 8.08155E-05 | 4.5899E-06 | 4.28476E-06 |
| **GAL3ST1** | 1427.90295 | | 2348.29858 | 2446.17163 | 185.710052 | 275.726227 | 177.544937 | | 3.30486346 | | 3.102499918 | 4.048180107 | | 1.82094E-05 | 1.92765E-05 | 4.75099E-06 |
| **GAL3ST4** | 2651.16138 | | 2464.71973 | 5630.79248 | 1384.72388 | 557.112549 | 1279.68933 | | 1.282822043 | | 2.194081794 | 2.057890635 | | 0.007954707 | 0.000200761 | 0.000266348 |
| **GALNT14** | 5376.99023 | | 6119.20605 | 5975.64746 | 1839.65125 | 2863.18652 | 1305.63818 | | 1.901338499 | | 1.207972502 | 2.108884753 | | 0.000478603 | 0.01004432 | 0.000219137 |
| **GATA3** | 95.8695297 | | 85.1861649 | 167.369629 | 1256.34717 | 2026.30481 | 1690.77405 | | -3.385499534 | | -4.633853204 | -3.03208256 | | 2.36693E-05 | 2.94477E-06 | 3.54001E-05 |
| **GGT5** | 618.542847 | | 704.205627 | 750.40448 | 4894.51904 | 2058.64819 | 3343.30762 | | -2.614701738 | | -1.477457258 | -2.105551384 | | 4.97127E-05 | 0.003438503 | 0.000275148 |
| **GIMAP5** | 2559.90088 | | 2897.27612 | 4358.04785 | 1272.55005 | 254.703125 | 1573.32129 | | 1.358302514 | | 3.518677491 | 1.387769422 | | 0.005675741 | 8.68062E-06 | 0.004597649 |
| **GINS2** | 438.787476 | | 436.342468 | 690.629639 | 240.550598 | 167.376343 | 304.557861 | | 1.307559069 | | 1.323868521 | 1.572029711 | | 0.01604804 | 0.015105218 | 0.004191391 |
| **GIT2** | 3054.91943 | | 2894.43652 | 2402.94971 | 1637.73828 | 1389.95129 | 1143.11621 | | 1.24183536 | | 1.126817875 | 1.102596644 | | 0.009366872 | 0.016645564 | 0.018985506 |
| **GJA1** | 11147.5986 | | 17402.5859 | 13813.5127 | 2881.62158 | 1689.93506 | 6170.36963 | | 2.285593924 | | 3.43125431 | 0.873574364 | | 0.000108787 | 4.16386E-05 | 0.047607619 |
| **GLB1L** | 327.246948 | | 378.605164 | 249.215225 | 114.666603 | 124.52153 | 159.790436 | | 1.878763061 | | 1.517208702 | 1.214855065 | | 0.003219036 | 0.008618109 | 0.03618918 |
| **GLDC** | 513.455078 | | 860.380249 | 155.414658 | 1996.69458 | 2166.99805 | 2335.39868 | | -1.579123442 | | -1.26326644 | -3.642647928 | | 0.00258121 | 0.00891538 | 9.99008E-06 |
| **GLRX** | 1020.45746 | | 1130.13647 | 2059.0144 | 351.478088 | 268.449005 | 478.005615 | | 1.938297218 | | 2.092992489 | 2.282724871 | | 0.000785677 | 0.00042582 | 0.00016643 |
| **GLT25D1** | 2178.26636 | | 1715.08142 | 3558.90381 | 960.955994 | 409.142151 | 1072.09827 | | 1.544384426 | | 2.091979311 | 1.724591507 | | 0.002588997 | 0.000332806 | 0.001082286 |
| **GNLY** | 2466.79688 | | 1395.16003 | 3573.61743 | 1429.59351 | 296.749359 | 718.374146 | | 1.131349752 | | 2.25290552 | 2.360422969 | | 0.016216327 | 0.000220564 | 0.000106232 |
| **GOT1** | 4809.14746 | | 3532.38623 | 3126.68555 | 12883.79 | 14229.415 | 5798.89062 | | -1.075736141 | | -1.900775578 | -1.078901507 | | 0.01840079 | 0.000430221 | 0.019074386 |
| **GPC3** | 105.087753 | | 114.528069 | 75.4082947 | 5394.31592 | 10523.6865 | 1341.14709 | | -5.367929885 | | -6.527597941 | -3.809083568 | | 1.53119E-06 | 8.58182E-07 | 1.37027E-05 |
| **GPC5** | 207.410049 | | 227.163101 | 247.375992 | 1394.69495 | 732.574707 | 972.399963 | | -2.349271479 | | -1.677863151 | -1.618626296 | | 0.000204965 | 0.002441841 | 0.003469797 |
| **GPD1L** | 1274.88037 | | 963.550171 | 1442.87341 | 3290.43311 | 3227.85669 | 2799.74707 | | -1.024895178 | | -1.66350105 | -0.972366941 | | 0.027225833 | 0.001373356 | 0.034277245 |
| **GPIHBP1** | 1033.36292 | | 1244.66455 | 373.363037 | 3216.89697 | 2819.52319 | 2067.71558 | | -1.290454339 | | -1.11344351 | -2.255635178 | | 0.007797264 | 0.017015217 | 0.000213498 |
| **GPR65** | 593.653625 | | 725.975403 | 1044.68079 | 189.449173 | 335.561249 | 217.151123 | | 2.044268468 | | 1.145321592 | 2.627760387 | | 0.000898801 | 0.021914914 | 9.75185E-05 |
| **GPR98** | 224.924667 | | 364.407471 | 119.549736 | 1525.56445 | 1052.77295 | 725.202759 | | -2.364459247 | | -1.496850993 | -2.176869649 | | 0.000182339 | 0.004066501 | 0.000740563 |
| **GRAMD4** | 1047.19031 | | 1001.41071 | 343.935394 | 139.594131 | 139.075989 | 158.424713 | | 3.230373195 | | 2.814758357 | 1.661714674 | | 2.73259E-05 | 0.000116722 | 0.005895705 |
| **GRB10** | 11302.4648 | | 9909.04395 | 14612.6562 | 5847.99707 | 2767.77393 | 5079.15088 | | 1.282682053 | | 1.950979465 | 1.249537848 | | 0.006803735 | 0.000364182 | 0.015162106 |
| **GRB14** | 639.744751 | | 502.598358 | 248.295609 | 2302.05664 | 4172.27979 | 3046.94434 | | -1.476759892 | | -2.979411759 | -3.404655864 | | 0.003727393 | 1.9298E-05 | 1.19118E-05 |
| **GRB7** | 1178.08911 | | 1727.38611 | 870.87384 | 3300.40405 | 4536.9502 | 4493.25244 | | -1.142684496 | | -1.297698055 | -2.375602227 | | 0.015307162 | 0.008048879 | 0.000102698 |
| **GRB7** | 215.706451 | | 217.697983 | 118.630127 | 873.709656 | 746.320618 | 745.688721 | | -1.595954928 | | -1.769681592 | -2.232765315 | | 0.003930173 | 0.001701885 | 0.000606936 |
| **GRHPR** | 3525.9707 | | 3217.19751 | 1272.74487 | 11791.9648 | 7920.86279 | 3664.25439 | | -1.392102405 | | -1.172155659 | -1.556694428 | | 0.004108073 | 0.011696243 | 0.002304819 |
| **GSTM3** | 312.497803 | | 177.944427 | 144.379303 | 3481.12866 | 7430.86279 | 1294.71228 | | -3.087026231 | | -5.348780265 | -2.819129456 | | 1.89022E-05 | 1.28482E-06 | 7.15574E-05 |
| **GSTO2** | 276.546722 | | 480.828583 | 189.440353 | 2026.60767 | 1521.75012 | 1260.56909 | | -2.4797473 | | -1.621747902 | -2.393107216 | | 0.000107305 | 0.002043582 | 0.000211184 |
| **GZMA** | 2728.59448 | | 1766.19312 | 2338.57666 | 585.796814 | 853.053345 | 584.532593 | | 2.603343336 | | 1.118594837 | 2.128692905 | | 5.6719E-05 | 0.019037934 | 0.000262647 |
| **HADH** | 1465.69763 | | 1057.255 | 763.279114 | 6350.28662 | 5091.63672 | 2331.30151 | | -1.768203108 | | -2.155778638 | -1.522027388 | | 0.000834066 | 0.000184933 | 0.003081873 |
| **HCK** | 1957.95081 | | 1794.5885 | 2985.98462 | 1056.927 | 763.300781 | 643.258972 | | 1.248691587 | | 1.30267138 | 2.299698422 | | 0.009948188 | 0.007881028 | 0.000136051 |
| **HCP5** | 2858.57129 | | 2261.21948 | 2998.85913 | 1176.5791 | 761.683655 | 704.716797 | | 1.634261748 | | 1.640470804 | 2.158815794 | | 0.001633334 | 0.00168152 | 0.000216717 |
| **HDAC11** | 677.53949 | | 604.821777 | 400.951416 | 2618.63623 | 1389.14282 | 1208.67126 | | -1.582248018 | | -1.135733824 | -1.316546297 | | 0.002266999 | 0.017741768 | 0.021501351 |
| **HEY1** | 723.630615 | | 988.159485 | 1957.85681 | 315.33316 | 463.317108 | 307.289307 | | 1.61665914 | | 1.134780045 | 2.919081635 | | 0.003394535 | 0.021111965 | 3.12994E-05 |
| **HIF3A** | 341.996124 | | 177.944427 | 183.003052 | 954.724121 | 604.818848 | 1059.80664 | | -1.06867924 | | -1.788810002 | -2.169563759 | | 0.03080616 | 0.00188145 | 0.000483148 |
| **HIGD1A** | 833.327454 | | 707.991699 | 350.372681 | 3008.75195 | 6112.875 | 1892.90222 | | -1.493817498 | | -2.988371169 | -2.200057504 | | 0.003182752 | 1.74465E-05 | 0.00026949 |
| **HILPDA** | 20316.9668 | | 17984.6914 | 19430.5098 | 1241.39062 | 987.277832 | 2629.03076 | | 4.423940634 | | 4.261882067 | 2.706947665 | | 2.05592E-06 | 2.39517E-06 | 3.0419E-05 |
| **HIST1H2BD** | 1394.71729 | | 2068.13086 | 1675.53552 | 619.448975 | 739.851929 | 596.824158 | | 1.554288897 | | 1.55331344 | 1.657313873 | | 0.002920173 | 0.002503978 | 0.001807776 |
| **HK2** | 4638.61035 | | 6582.05078 | 7075.50537 | 148.318756 | 289.472137 | 215.785385 | | 5.288039906 | | 4.577247118 | 5.181552644 | | 1.51321E-06 | 2.11026E-06 | 1.43562E-06 |
| **HLA-DOB** | 699.663208 | | 804.536011 | 763.279114 | 314.086792 | 97.8383408 | 357.82135 | | 1.575342387 | | 2.990630572 | 1.444140691 | | 0.004011219 | 5.70141E-05 | 0.006494928 |
| **HLA-DQA1** | 2845.66577 | | 1253.18311 | 3908.35669 | 859.999573 | 526.386475 | 645.990417 | | 2.094680065 | | 1.301184778 | 2.649372543 | | 0.000269947 | 0.008918268 | 4.51433E-05 |
| **HLA-F** | 41168.5898 | | 35523.5781 | 39795.3477 | 7356.1123 | 10273.0264 | 8229.89062 | | 2.866133234 | | 1.810050307 | 1.944440044 | | 1.97711E-05 | 0.000630018 | 0.000350462 |
| **HLA-F** | 1993.90186 | | 1371.49719 | 791.787109 | 300.376648 | 506.171936 | 200.76236 | | 3.125138712 | | 1.481919043 | 2.384421251 | | 2.00529E-05 | 0.003944159 | 0.000242321 |
| **HLA-F** | 662.790344 | | 765.728943 | 605.105591 | 175.739044 | 157.67337 | 165.253357 | | 2.294140497 | | 2.258215347 | 2.329374617 | | 0.013899073 | 0.000354941 | 0.000380414 |
| **HLA-G** | 2283.35425 | | 2016.07251 | 832.250122 | 949.738647 | 992.129333 | 508.051666 | | 1.629823626 | | 1.091533066 | 0.99704496 | | 0.004263136 | 0.020004565 | 0.0398505 |
| **HLX** | 1202.0564 | | 845.236023 | 1890.7251 | 391.362122 | 205.379669 | 379.673035 | | 2.01284431 | | 2.040482666 | 2.537414693 | | 0.000540401 | 0.000638106 | 8.17033E-05 |
| **HMHA1** | 2610.60107 | | 2131.54712 | 4980.62598 | 1326.14429 | 712.360168 | 1319.29541 | | 1.325056834 | | 1.65179129 | 1.842463489 | | 0.006869505 | 0.002504539 | 0.001465826 |
| **HMOX1** | 5528.16895 | | 4020.78687 | 2278.802 | 722.898193 | 185.165131 | 294.997742 | | 3.3241984 | | 4.451697403 | 3.182017064 | | 9.49313E-06 | 2.65985E-06 | 1.69372E-05 |
| **HOGA1** | 166.849854 | | 131.565292 | 83.6848145 | 474.869324 | 330.709778 | 310.020782 | | -1.076369749 | | -1.414861785 | -1.333979018 | | 0.044307262 | 0.0127152 | 0.029692924 |
| **HOXB5** | 568.764465 | | 584.945007 | 1601.04688 | 6779.04004 | 3159.12744 | 5304.49658 | | -3.190150802 | | -2.367661739 | -1.829092589 | | 1.23578E-05 | 0.000102662 | 0.000675512 |
| **HOXB6** | 1353.23535 | | 1151.90625 | 1081.46533 | 4897.01172 | 7853.75049 | 6569.1626 | | -1.514909499 | | -2.64645003 | -2.66304619 | | 0.013868228 | 3.82372E-05 | 4.44709E-05 |
| **HOXD8** | 1023.2229 | | 1463.30896 | 416.584839 | 3229.3606 | 5582.4458 | 1813.68982 | | -1.30978422 | | -1.82347942 | -1.908131363 | | 0.007132943 | 0.00064117 | 0.000752185 |
| **HP** | 3436.55396 | | 8161.78125 | 60235.5938 | 169.507156 | 84.9010391 | 398.793243 | | 4.684642794 | | 6.580640363 | 6.64385619 | | 7.36682E-05 | 9.13877E-07 | 6.9377E-07 |
| **HPCAL1** | 7244.60254 | | 8771.33496 | 13800.6377 | 3874.9834 | 2491.23926 | 4471.40088 | | 1.274512675 | | 1.928118116 | 1.359025123 | | 0.007258644 | 0.000402765 | 0.004725929 |
| **HPGD** | 129.055145 | | 270.702698 | 263.00943 | 2354.40454 | 1066.5188 | 1084.38989 | | -3.848214752 | | -1.95433442 | -1.706465975 | | 7.22431E-06 | 0.000673489 | 0.002284945 |
| **HPS4** | 485.800415 | | 823.466248 | 697.986511 | 253.014359 | 332.326935 | 428.839294 | | 1.377436966 | | 1.340112932 | 1.042084654 | | 0.011378134 | 0.009151629 | 0.035392292 |
| **HRG** | 93.1040649 | | 95.5978088 | 104.835922 | 1552.98474 | 1049.53857 | 426.107849 | | -3.74073988 | | -3.506931586 | -1.514299354 | | 1.15866E-05 | 1.5511E-05 | 0.011616644 |
| **HSD17B7** | 1055.48669 | | 1248.45056 | 1996.48059 | 319.072296 | 621.799072 | 605.018494 | | 2.125778707 | | 1.07086271 | 1.864159596 | | 0.000404257 | 0.024576683 | 0.000736818 |
| **HSF4** | 913.526001 | | 3005.17847 | 640.97052 | 276.695496 | 80.0495529 | 323.67807 | | 2.131287926 | | 5.177797736 | 1.380175242 | | 0.000445665 | 1.8889E-06 | 0.009379863 |
| **HSPA2** | 352.136169 | | 287.739929 | 161.851959 | 5341.96826 | 13069.1006 | 1353.43872 | | -3.537203752 | | -5.452101464 | -2.72781335 | | 7.36902E-06 | 1.14324E-06 | 8.39109E-05 |
| **HSPA9** | 131.820602 | | 183.623505 | 156.334274 | 722.898193 | 637.970703 | 671.939331 | | -2.075986463 | | -1.812967873 | -1.672450162 | | 0.001096998 | 0.001647302 | 0.004045467 |
| **HSPB7** | 265.484863 | | 205.393311 | 260.25058 | 3792.72266 | 3305.48047 | 3464.85767 | | -3.443936335 | | -4.004531666 | -3.541899197 | | 9.66841E-06 | 4.12907E-06 | 9.00767E-06 |
| **HSPB8** | 2763.62354 | | 3015.59033 | 2141.77954 | 653.101135 | 242.574402 | 710.179749 | | 2.460509789 | | 3.64602902 | 1.699042273 | | 8.40788E-05 | 7.00792E-06 | 0.001388637 |
| **HYAL1** | 4975.99756 | | 3034.52051 | 5395.37158 | 20507.873 | 9770.89746 | 9871.49805 | | -1.665757683 | | -1.567155485 | -1.146114095 | | 0.001149405 | 0.001820205 | 0.013492486 |
| **ICAM1** | 1135.68518 | | 1073.3457 | 615.221313 | 648.115601 | 473.828674 | 303.192139 | | 1.192097566 | | 1.22334095 | 1.431051547 | | 0.015142947 | 0.013282416 | 0.00788812 |
| **IDO1** | 3149.86719 | | 1152.85278 | 3329.00049 | 152.057892 | 91.3696899 | 180.276398 | | 4.697381093 | | 3.605299158 | 4.435195094 | | 2.41858E-06 | 1.40864E-05 | 2.87943E-06 |
| **IFI30** | 22223.2949 | | 17973.334 | 40608.2852 | 12205.7617 | 8004.14697 | 7663.11279 | | 1.250528254 | | 1.286249263 | 2.080430034 | | 0.007728501 | 0.00932006 | 0.000548051 |
| **IFI44** | 648.963013 | | 808.322021 | 496.591217 | 79.768074 | 415.610809 | 234.905609 | | 3.369892186 | | 0.99766451 | 1.54662017 | | 3.89152E-05 | 0.038735062 | 0.006029726 |
| **IGF2** | 234.142899 | | 191.195618 | 106.675148 | 1024.52124 | 416.419403 | 923.233643 | | -1.715474489 | | -1.170483234 | -2.722062568 | | 0.002203966 | 0.026211938 | 0.000132963 |
| **IGFBP2** | 401.914581 | | 212.01889 | 361.408051 | 5586.25781 | 4219.98584 | 3382.91382 | | -3.413537039 | | -4.28878304 | -3.064737901 | | 8.81075E-06 | 2.88185E-06 | 2.06784E-05 |
| **IGFBP3** | 53246.3086 | | 52135.8242 | 60237.4336 | 11927.8193 | 14081.4443 | 12796.8926 | | 2.531181577 | | 1.845590144 | 1.913167356 | | 4.76507E-05 | 0.000516265 | 0.000414842 |
| **IGFBP6** | 361.354401 | | 271.6492 | 138.861618 | 1256.34717 | 1059.24158 | 793.489319 | | -1.399621051 | | -1.939097543 | -2.105837894 | | 0.008029464 | 0.000715792 | 0.000828646 |
| **IGFLR1** | 4985.21582 | | 6656.82568 | 10682.2285 | 3011.24487 | 1220.14929 | 2683.65991 | | 1.073586432 | | 2.570038943 | 1.790653406 | | 0.019416923 | 4.89166E-05 | 0.000699031 |
| **IKZF1** | 613.011902 | | 570.747314 | 1035.48462 | 108.434723 | 133.415924 | 236.271332 | | 2.846990368 | | 2.044732588 | 2.487000919 | | 0.000107197 | 0.000936044 | 0.000142125 |
| **IKZF3** | 1696.15332 | | 1252.23657 | 2277.88232 | 345.246185 | 223.168457 | 473.908417 | | 2.690103407 | | 2.489710975 | 2.430388285 | | 5.73446E-05 | 0.000118944 | 0.000101198 |
| **IL10RA** | 679.383118 | | 579.26593 | 765.118286 | 165.768036 | 221.551285 | 147.498871 | | 2.402944369 | | 1.377222729 | 2.796771033 | | 0.000270712 | 0.009862326 | 9.0145E-05 |
| **IL32** | 2100.83325 | | 1888.29333 | 1636.91174 | 704.202515 | 205.379669 | 663.744934 | | 1.953501619 | | 3.187486286 | 1.456129928 | | 0.000502205 | 5.37699E-05 | 0.004278887 |
| **IL32** | 1259.20947 | | 2238.50317 | 1753.70264 | 266.724487 | 114.81855 | 419.279205 | | 2.63682949 | | 4.225368828 | 2.281536657 | | 8.04432E-05 | 4.21143E-06 | 0.00018085 |
| **IL32** | 701.506897 | | 1033.59216 | 953.639038 | 314.086792 | 88.1353683 | 279.974701 | | 1.579034637 | | 3.501408913 | 2.118849022 | | 0.00394675 | 1.81901E-05 | 0.000456692 |
| **IL4I1** | 2273.21411 | | 1798.37463 | 2202.47412 | 277.941895 | 114.009972 | 367.381439 | | 3.426844782 | | 3.917936143 | 2.790248637 | | 1.10615E-05 | 6.62404E-06 | 3.94918E-05 |
| **IL4R** | 6992.02295 | | 5271.13037 | 12560.999 | 4614.08447 | 2021.45337 | 5217.08984 | | 0.971272778 | | 1.486270244 | 0.985129081 | | 0.030718165 | 0.002787528 | 0.0283021 |
| **ILDR1** | 730.083374 | | 862.273315 | 800.983215 | 3579.59229 | 2564.82007 | 2403.6853 | | -1.929008927 | | -1.50233358 | -1.507955262 | | 0.000503959 | 0.002913014 | 0.003236403 |
| **IMPDH2** | 330.012421 | | 367.247009 | 140.700851 | 1099.30371 | 749.554932 | 844.021301 | | -1.329811859 | | -0.993511213 | -2.184040107 | | 0.009698859 | 0.040250104 | 0.000596783 |
| **INMT** | 3313.95166 | | 2725.01074 | 2654.92383 | 8929.03906 | 8465.03809 | 6353.37744 | | -1.058354903 | | -1.521368533 | -1.432706648 | | 0.020388147 | 0.002261595 | 0.003612666 |
| **INSR** | 3979.50732 | | 4291.48975 | 3538.67212 | 1078.11536 | 1010.72668 | 1052.97803 | | 2.239718761 | | 2.192321355 | 1.745271467 | | 0.000149358 | 0.00017116 | 0.000994454 |
| **INTS4** | 1312.67517 | | 1312.81348 | 1930.26843 | 570.840271 | 663.036682 | 797.586487 | | 1.588198186 | | 1.055385362 | 1.37758438 | | 0.002606516 | 0.025947209 | 0.005731817 |
| **IRF7** | 478.425842 | | 605.768311 | 759.600647 | 286.666504 | 195.676682 | 350.992676 | | 1.174687812 | | 1.614848211 | 1.468582495 | | 0.024998896 | 0.003841952 | 0.005907392 |
| **ISG20** | 1034.28479 | | 760.049866 | 686.951172 | 144.579636 | 261.171783 | 192.567963 | | 3.162745891 | | 1.560842085 | 2.266359183 | | 3.11493E-05 | 0.003947629 | 0.000393848 |
| **ITGB2** | 2370.92725 | | 1850.43274 | 3614.08057 | 467.391052 | 569.241272 | 396.061768 | | 2.730875258 | | 1.749848289 | 3.328346924 | | 4.338E-05 | 0.002885159 | 1.07184E-05 |
| **ITM2C** | 1126.46704 | | 943.673401 | 705.343445 | 2658.52026 | 2503.36792 | 3793.99878 | | -0.89481942 | | -1.337820064 | -2.382522607 | | 0.048947454 | 0.006148907 | 0.000106753 |
| **ITPKC** | 2818.93311 | | 2681.47119 | 8525.73535 | 1930.63672 | 772.19519 | 2377.73633 | | 0.88797854 | | 1.867491978 | 1.661599436 | | 0.048467938 | 0.00063541 | 0.001236315 |
| **ITPR3** | 10899.6279 | | 8547.95801 | 13119.2041 | 5335.73633 | 3632.95605 | 4924.82324 | | 1.363588586 | | 1.367637335 | 1.13245906 | | 0.004640845 | 0.004649459 | 0.014830851 |
| **KALRN** | 334.621552 | | 1001.41071 | 881.909241 | 205.652069 | 124.52153 | 215.785385 | | 1.129318845 | | 2.965237763 | 2.414245552 | | 0.038058888 | 4.71667E-05 | 0.000200592 |
| **KCNE3** | 902.464172 | | 1766.19312 | 574.758362 | 398.840363 | 208.613983 | 258.123016 | | 1.582690552 | | 3.069418278 | 1.590270777 | | 0.003273865 | 2.41712E-05 | 0.004525885 |
| **KCNJ1** | 468.285797 | | 394.695892 | 383.47876 | 50995.4805 | 28666.6348 | 28082.1465 | | -6.359077326 | | -6.195633149 | -6.146790309 | | 8.17629E-07 | 2.06488E-05 | 8.92854E-07 |
| **KCNQ1** | 885.871338 | | 710.831238 | 1348.1532 | 4749.93945 | 3964.47437 | 2594.88745 | | -2.06927656 | | -2.380691085 | -0.942740146 | | 0.000276452 | 9.2954E-05 | 0.03965503 |
| **KCTD1** | 193.582703 | | 202.553772 | 173.806931 | 741.593811 | 794.026917 | 614.578613 | | -1.508057431 | | -1.969356066 | -1.37939323 | | 0.006276891 | 0.000789915 | 0.012633453 |
| **KCTD3** | 318.950562 | | 697.580017 | 514.983459 | 100.956467 | 269.257599 | 165.253357 | | 2.025871477 | | 1.396083839 | 2.122867084 | | 0.002179943 | 0.008037481 | 0.000839164 |
| **KIAA0922** | 966.991699 | | 1303.34827 | 1278.26257 | 385.130219 | 406.716431 | 644.624695 | | 1.730036573 | | 1.711329311 | 1.179938737 | | 0.001759716 | 0.001572399 | 0.015681332 |
| **KIT** | 641.58844 | | 850.915161 | 482.797028 | 1680.11511 | 4472.26367 | 1745.40332 | | -1.01642959 | | -2.286292476 | -1.656278617 | | 0.032139212 | 0.00014682 | 0.002031599 |
| **KLHL3** | 174.224442 | | 170.37233 | 308.070465 | 1152.89795 | 361.435852 | 1171.79663 | | -2.319155915 | | -1.146355559 | -1.613658811 | | 0.000265363 | 0.031550173 | 0.003065212 |
| **KNG1** | 92.1822433 | | 98.4373474 | 99.3182449 | 47554.2344 | 39023.7539 | 15297.5449 | | -6.64385619 | | -6.64385619 | -6.64385619 | | 6.37403E-07 | 6.28448E-07 | 7.97622E-07 |
| **KNTC1** | 665.555786 | | 660.666016 | 1390.45544 | 234.31871 | 293.515045 | 249.92865 | | 1.930576911 | | 1.19465645 | 2.788705209 | | 0.001152925 | 0.01878145 | 5.24249E-05 |
| **KRT18** | 6271.1582 | | 4092.72192 | 12534.3301 | 1804.75269 | 2274.53931 | 2959.5376 | | 2.163336782 | | 0.93886603 | 1.863438472 | | 0.000303668 | 0.039312244 | 0.000511816 |
| **L3HYPDH** | 2059.35132 | | 1438.69971 | 1693.00818 | 415.043274 | 777.855286 | 826.266846 | | 2.701170945 | | 0.960982597 | 1.151007228 | | 5.00113E-05 | 0.03874106 | 0.016495254 |
| **LAIR1** | 577.982666 | | 747.745239 | 2023.14941 | 127.130371 | 152.821869 | 132.47583 | | 2.534955898 | | 2.266598966 | 4.227696536 | | 0.000235672 | 0.000354954 | 4.4319E-06 |
| **LAMA4** | 1602.12744 | | 1462.36255 | 1993.7218 | 272.95639 | 267.640442 | 270.414612 | | 2.949269467 | | 2.462321907 | 3.138559312 | | 3.27591E-05 | 0.000115012 | 1.97648E-05 |
| **LAPTM5** | 615.777405 | | 441.075043 | 1277.34302 | 206.898438 | 200.528168 | 143.401672 | | 1.983810433 | | 1.094519931 | 3.510389115 | | 0.001047022 | 0.035465464 | 1.46517E-05 |
| **LARP7** | 194.504532 | | 295.312042 | 172.887314 | 595.767822 | 622.607666 | 736.128601 | | -1.176674667 | | -1.05924512 | -1.67683037 | | 0.026749861 | 0.032830991 | 0.003620608 |
| **LAT2** | 752.207092 | | 966.389709 | 1487.93445 | 119.652115 | 405.907837 | 239.002808 | | 2.991527326 | | 1.28774121 | 2.945537321 | | 6.13008E-05 | 0.011617314 | 3.51934E-05 |
| **LCK** | 719.021484 | | 513.956543 | 869.034607 | 342.753448 | 254.703125 | 259.48877 | | 1.486700005 | | 1.003897327 | 2.11269907 | | 0.006294242 | 0.046010215 | 0.000504565 |
| **LCN12** | 107.853226 | | 132.51181 | 148.057755 | 973.4198 | 427.739532 | 640.527527 | | -2.835069835 | | -1.767209134 | -1.672387002 | | 0.000115743 | 0.002839152 | 0.004264004 |
| **LDHB** | 20361.2129 | | 20194.8008 | 28433.5254 | 68125.6719 | 44901.332 | 44106.2578 | | -1.339010453 | | -1.231989099 | -0.977564104 | | 0.004985265 | 0.00833471 | 0.028536476 |
| **LEPRE1** | 601.028198 | | 1026.02002 | 1736.22998 | 175.739044 | 336.369843 | 576.338196 | | 2.156904675 | | 1.639141318 | 1.759875523 | | 0.001220621 | 0.002388064 | 0.001175097 |
| **LEPREL2** | 400.992767 | | 304.777161 | 429.459442 | 1287.50659 | 621.799072 | 1018.83478 | | -1.28779289 | | -1.011007393 | -0.95962747 | | 0.010674644 | 0.039994814 | 0.04753273 |
| **LGI4** | 856.373047 | | 1027.91309 | 5160.87012 | 339.014313 | 138.26741 | 319.580872 | | 1.74633078 | | 2.860240682 | 4.140081713 | | 0.001804501 | 0.000214118 | 3.26271E-06 |
| **LHFPL2** | 2313.77441 | | 1723.6001 | 2564.80176 | 472.376556 | 674.356873 | 422.010651 | | 2.680205071 | | 1.418337123 | 2.772374567 | | 4.98035E-05 | 0.00478901 | 3.84177E-05 |
| **LILRB2** | 380.712677 | | 457.165741 | 810.179382 | 178.231796 | 132.607346 | 187.105042 | | 1.494831862 | | 1.712404662 | 2.519724369 | | 0.009316713 | 0.003584747 | 0.000164336 |
| **LINC00472** | 117.071449 | | 133.458328 | 140.700851 | 406.318634 | 669.505371 | 464.348297 | | -1.421523178 | | -2.364507957 | -1.228396842 | | 0.015025083 | 0.000268564 | 0.02789131 |
| **LIPH** | 142.882477 | | 135.351349 | 211.511078 | 493.564972 | 599.158752 | 641.89325 | | -1.383430299 | | -2.193124059 | -1.172724124 | | 0.014201887 | 0.000496488 | 0.02638614 |
| **LMO3** | 172.380798 | | 144.816483 | 114.951668 | 3390.14307 | 2145.97485 | 2955.44043 | | -3.906612387 | | -3.927890101 | -4.439209777 | | 5.44455E-06 | 5.7304E-06 | 3.6883E-06 |
| **LOC100505806** | 213.862808 | | 349.263275 | 179.3246 | 76.0289459 | 71.9637375 | 114.721344 | | 1.862974156 | | 2.181511814 | 1.246237247 | | 0.006440633 | 0.001235759 | 0.045155488 |
| **LOC101060198** | 1311.7533 | | 748.691711 | 2185.00146 | 599.506958 | 332.326935 | 483.468506 | | 1.514703181 | | 1.203283806 | 2.343320912 | | 0.004398006 | 0.016977424 | 0.000134099 |
| **LOC151174** | 113.384155 | | 150.49556 | 127.826256 | 616.956177 | 306.452332 | 397.427521 | | -2.084622337 | | -1.102411018 | -1.117520675 | | 0.001071226 | 0.041643191 | 0.046512343 |
| **LOC285074** | 1414.99744 | | 1114.04578 | 1259.87036 | 560.869263 | 520.726379 | 523.074707 | | 1.721747004 | | 1.14899988 | 1.496534387 | | 0.001462489 | 0.018094836 | 0.003999454 |
| **LOC400043** | 561.389832 | | 325.600464 | 684.192322 | 3471.15771 | 4131.04199 | 3437.54321 | | -2.252958585 | | -3.614370414 | -2.267149214 | | 0.000162898 | 6.28401E-06 | 0.000158816 |
| **LOC441081** | 881.262268 | | 1234.25293 | 2256.7312 | 291.652008 | 329.092621 | 574.972473 | | 2.00473453 | | 1.933166583 | 2.107837319 | | 0.000697842 | 0.000699519 | 0.00028638 |
| **LOC653562** | 4091.04785 | | 1737.79773 | 14331.2549 | 1850.86865 | 704.274353 | 1149.94495 | | 1.483992125 | | 1.371435418 | 3.544175416 | | 0.002938079 | 0.005853022 | 5.78151E-06 |
| **LOXL2** | 7645.59521 | | 5579.69385 | 21859.209 | 811.390869 | 1027.70691 | 1446.30835 | | 3.633852658 | | 2.558236618 | 3.811730447 | | 5.47569E-06 | 5.22358E-05 | 3.86011E-06 |
| **LPAR1** | 326.325134 | | 158.067657 | 228.064117 | 1280.02832 | 2315.7771 | 1031.12634 | | -1.572812908 | | -3.903887077 | -1.820735308 | | 0.003281579 | 5.35413E-06 | 0.001730363 |
| **LPAR6** | 3422.72656 | | 3267.36255 | 9003.93457 | 1322.40515 | 1838.71399 | 1093.94995 | | 1.718684106 | | 0.901453094 | 2.958870006 | | 0.001095932 | 0.044634361 | 1.7941E-05 |
| **LPCAT1** | 2920.3335 | | 3077.11353 | 4015.95166 | 372.666473 | 673.548279 | 427.473572 | | 3.361816047 | | 2.267719659 | 3.346390109 | | 1.08872E-05 | 0.000145832 | 1.00403E-05 |
| **LPHN1** | 367.807159 | | 333.172546 | 424.861359 | 2275.88281 | 1070.56177 | 2597.6189 | | -2.241168338 | | -1.653146518 | -2.443207749 | | 0.000204532 | 0.002117537 | 0.000106401 |
| **LRMP** | 1621.4856 | | 1758.62109 | 3080.70483 | 522.231628 | 609.670349 | 550.389282 | | 2.021622346 | | 1.583488207 | 2.591709508 | | 0.000440536 | 0.002326072 | 5.68504E-05 |
| **LRRC41** | 1141.21619 | | 1309.02734 | 2241.09766 | 194.434677 | 253.085968 | 129.744385 | | 2.924682921 | | 2.381415179 | 4.393656177 | | 4.50111E-05 | 0.000156189 | 3.68427E-06 |
| **LTB4R** | 398.227295 | | 310.456238 | 494.751984 | 209.39119 | 120.478622 | 159.790436 | | 1.354512653 | | 1.269746462 | 2.120413043 | | 0.014662067 | 0.024699409 | 0.000888376 |
| **LY86** | 565.077148 | | 644.575317 | 923.291809 | 282.927399 | 257.937439 | 214.419647 | | 1.427292075 | | 1.334321263 | 2.484378576 | | 0.008357688 | 0.010842599 | 0.000158533 |
| **MAF** | 539.266113 | | 792.231323 | 773.394836 | 314.086792 | 163.333435 | 352.358398 | | 1.210017866 | | 2.259111506 | 1.485425069 | | 0.02018041 | 0.000342202 | 0.005455967 |
| **MAFB** | 2752.56177 | | 4190.21289 | 12423.0566 | 1425.85437 | 875.693604 | 2992.31494 | | 1.29328498 | | 2.363599918 | 1.833744258 | | 0.007526203 | 9.86635E-05 | 0.000577934 |
| **MAGI2** | 361.354401 | | 290.579468 | 335.658875 | 1059.41968 | 687.294128 | 942.353882 | | -1.145372577 | | -1.216534619 | -1.156733455 | | 0.021343829 | 0.016356589 | 0.022085391 |
| **MAGI2-AS3** | 316.185089 | | 210.12587 | 194.958038 | 1407.15869 | 483.531647 | 747.054443 | | -1.759020552 | | -1.23306831 | -1.530745825 | | 0.001489895 | 0.018669244 | 0.006056796 |
| **MAMDC2** | 213.862808 | | 227.163101 | 149.896973 | 838.811157 | 876.502197 | 721.105591 | | -1.547834418 | | -1.93760342 | -1.845316111 | | 0.004877752 | 0.000818227 | 0.002078121 |
| **MAOB** | 17186.457 | | 21776.4238 | 18106.2676 | 8871.70508 | 6577.80908 | 6656.56934 | | 1.36577552 | | 1.825606561 | 1.170374827 | | 0.004495388 | 0.000573294 | 0.011500185 |
| **MAP3K6** | 2974.72095 | | 2189.28442 | 4024.22803 | 953.477783 | 976.766296 | 1551.4696 | | 2.005007772 | | 1.233499755 | 1.30456369 | | 0.000368131 | 0.010273014 | 0.006836414 |
| **MAP4K4** | 500.549591 | | 691.90094 | 317.266602 | 123.391243 | 333.944092 | 189.836517 | | 2.376222172 | | 1.083044705 | 1.288383731 | | 0.000429592 | 0.028974759 | 0.022784222 |
| **MAP7D3** | 425.881958 | | 400.374969 | 428.539825 | 220.608582 | 169.802078 | 223.979767 | | 1.383999953 | | 1.177371339 | 1.428436628 | | 0.012398393 | 0.027806807 | 0.010593195 |
| **MBNL1** | 4771.35303 | | 6740.11865 | 8826.44922 | 2166.20166 | 3329.73804 | 3657.42578 | | 1.482618089 | | 1.145766801 | 1.038874243 | | 0.002888468 | 0.013405538 | 0.022282211 |
| **MCAM** | 1423.29382 | | 1513.47424 | 1670.01782 | 410.05777 | 564.389771 | 529.90332 | | 2.187391494 | | 1.474494846 | 1.844078793 | | 0.000269584 | 0.003914229 | 0.001267171 |
| **MCCD1** | 133.664246 | | 97.4908295 | 115.871284 | 1200.26025 | 350.115723 | 432.936493 | | -2.807606512 | | -1.942899387 | -1.39517372 | | 7.30872E-05 | 0.002038313 | 0.016893363 |
| **MDH2** | 1488.74316 | | 1909.11658 | 1621.27832 | 6067.35938 | 5089.21094 | 3712.05493 | | -1.683688577 | | -1.313925239 | -1.260639147 | | 0.001198001 | 0.006183915 | 0.008688363 |
| **MECOM** | 409.289154 | | 670.131165 | 859.838501 | 1977.9989 | 3292.54321 | 4019.34424 | | -1.885584241 | | -2.215316359 | -2.218087402 | | 0.000757805 | 0.000165838 | 0.000175755 |
| **MECOM** | 335.543365 | | 533.833313 | 658.443176 | 1698.81067 | 2897.14697 | 2668.63696 | | -1.948309207 | | -2.390101343 | -1.921280897 | | 0.000655687 | 9.83539E-05 | 0.000586558 |
| **MEF2C** | 3676.22778 | | 3804.0354 | 6202.79199 | 2157.47705 | 1789.3905 | 2293.06104 | | 1.108352476 | | 1.172239515 | 1.274964022 | | 0.017014621 | 0.012468479 | 0.007451181 |
| **MEF2C** | 1829.8175 | | 2068.13086 | 2428.69897 | 533.448975 | 1037.40991 | 827.632568 | | 2.164371938 | | 1.063829866 | 1.623079667 | | 0.000253431 | 0.022591986 | 0.001833634 |
| **MET** | 3697.42969 | | 4598.15967 | 3109.21289 | 503.53598 | 1229.85229 | 1014.73761 | | 3.261247109 | | 2.009441045 | 1.631839289 | | 1.18574E-05 | 0.000323681 | 0.001653477 |
| **METTL7B** | 1424.2157 | | 1679.11389 | 3087.14209 | 509.767853 | 109.967064 | 740.22583 | | 1.870548936 | | 3.870908204 | 2.11839837 | | 0.000819306 | 7.33841E-06 | 0.000247108 |
| **MFSD12** | 1020.45746 | | 835.770935 | 758.68103 | 279.188263 | 332.326935 | 467.079773 | | 2.272584576 | | 1.361431644 | 1.012974645 | | 0.000261436 | 0.008310533 | 0.038578339 |
| **MID1** | 1450.02673 | | 872.684937 | 1094.33984 | 529.709839 | 362.244446 | 589.995483 | | 1.840323878 | | 1.301838782 | 1.11752325 | | 0.000910067 | 0.010417544 | 0.023190057 |
| **MINA** | 365.041687 | | 418.358704 | 336.578491 | 1677.62231 | 1030.13269 | 1123.99597 | | -1.81001744 | | -1.26194616 | -1.429537323 | | 0.001102852 | 0.011207422 | 0.006547812 |
| **MIOS** | 385.321777 | | 910.545471 | 640.050903 | 149.56514 | 460.891357 | 393.330322 | | 1.734477565 | | 1.024293251 | 1.068624491 | | 0.004092097 | 0.033212353 | 0.032999933 |
| **MLKL** | 3090.87061 | | 1750.10242 | 4343.33398 | 1230.17322 | 833.6474 | 998.348816 | | 1.680038801 | | 1.13866198 | 2.099543398 | | 0.001319627 | 0.016640078 | 0.000241296 |
| **MMP11** | 585.357239 | | 1092.27588 | 1408.84766 | 239.30423 | 94.6040192 | 307.289307 | | 1.719867962 | | 3.478603777 | 2.489542477 | | 0.002717737 | 1.77662E-05 | 0.000111305 |
| **MMP14** | 5612.05518 | | 3989.552 | 10365.8818 | 2305.7959 | 1486.17249 | 2898.07959 | | 1.639295762 | | 1.517384728 | 1.628709664 | | 0.001408369 | 0.002521437 | 0.001402417 |
| **MOXD1** | 830.562012 | | 551.817017 | 549.928772 | 16285.1504 | 19930.7227 | 5767.479 | | -3.929057526 | | -5.125702529 | -3.343728946 | | 4.24407E-06 | 1.28505E-06 | 1.00615E-05 |
| **MPC1** | 2519.34058 | | 3283.45337 | 2659.52173 | 20976.5098 | 17090.9844 | 11203.0859 | | -2.683245991 | | -2.29283309 | -2.271669212 | | 3.26669E-05 | 0.000102135 | 0.00011781 |
| **MPC1** | 871.122192 | | 861.326782 | 331.060822 | 4277.56299 | 7747.82666 | 1739.94043 | | -1.940873825 | | -3.043439777 | -2.140410884 | | 0.00045723 | 1.49408E-05 | 0.001029706 |
| **MPI** | 7591.20752 | | 6941.72607 | 6097.03662 | 3947.27319 | 2481.53613 | 2678.19702 | | 1.310957482 | | 1.599693608 | 1.009410406 | | 0.006088351 | 0.001626037 | 0.026221005 |
| **MRGPRF** | 188.051773 | | 230.949158 | 191.279572 | 1769.85413 | 619.373291 | 1192.28259 | | -2.836358434 | | -1.42333886 | -2.291727442 | | 4.83402E-05 | 0.00745668 | 0.000297847 |
| **MRPS28** | 138.273361 | | 151.442078 | 65.2925491 | 426.260651 | 432.591034 | 270.414612 | | -1.222400735 | | -1.579618845 | -1.482180601 | | 0.028568059 | 0.005450903 | 0.023026453 |
| **MRPS6** | 4418.29492 | | 4497.82959 | 3658.22192 | 19999.3516 | 29477.6426 | 11041.9297 | | -1.812318028 | | -2.658899228 | -1.827287204 | | 0.000617107 | 0.000592853 | 0.000607963 |
| **MRPS9** | 253.501175 | | 252.718948 | 85.5240402 | 705.448914 | 840.116028 | 595.458374 | | -1.047392106 | | -1.713637622 | -2.343384075 | | 0.038888585 | 0.001933574 | 0.000625744 |
| **MS4A7** | 3477.11426 | | 6305.66943 | 10340.1328 | 1292.49207 | 1933.31799 | 2368.17627 | | 1.775445835 | | 1.816231943 | 1.939468204 | | 0.000863714 | 0.000654415 | 0.00043467 |
| **MTFP1** | 3256.79858 | | 4747.70898 | 3231.52124 | 1648.95569 | 1300.19885 | 946.451111 | | 1.323844662 | | 1.975262673 | 1.792343752 | | 0.006339898 | 0.000365805 | 0.000838252 |
| **MTMR11** | 9343.5918 | | 12918.9551 | 17172.8594 | 3306.63599 | 1395.61145 | 3191.71167 | | 1.848122396 | | 3.309059327 | 2.217616157 | | 0.000552315 | 8.54434E-06 | 0.000133106 |
| **MUC1** | 2751.63989 | | 1674.38135 | 523.26001 | 7650.25684 | 7535.16943 | 4483.69238 | | -1.114909125 | | -2.056036936 | -3.025601153 | | 0.015754627 | 0.000250056 | 1.97937E-05 |
| **MUC1** | 1353.23535 | | 1000.46417 | 236.340637 | 3765.30249 | 5079.50781 | 2358.61621 | | -1.134046743 | | -2.231794508 | -3.073029643 | | 0.015587416 | 0.000142612 | 2.51372E-05 |
| **MUC20** | 425.881958 | | 334.11908 | 145.298904 | 1313.68042 | 1597.75671 | 1107.6073 | | -1.231968307 | | -2.235775347 | -2.564704561 | | 0.013425377 | 0.000203669 | 0.00015399 |
| **MXI1** | 4926.21924 | | 8211 | 5671.25537 | 3036.17236 | 3880.38184 | 2086.83594 | | 1.043556305 | | 1.220474768 | 1.296963567 | | 0.022392549 | 0.009269319 | 0.008236453 |
| **MYH10** | 613.933716 | | 563.175171 | 396.353363 | 2097.65112 | 1850.84277 | 1917.48535 | | -1.399843308 | | -1.66553414 | -2.060601485 | | 0.005383961 | 0.001583957 | 0.000423846 |
| **MYOG** | 188.973602 | | 333.172546 | 260.25058 | 1459.50647 | 728.531799 | 2034.93811 | | -2.549996947 | | -1.095785861 | -2.712682207 | | 0.000113276 | 0.026369315 | 6.11219E-05 |
| **NAT8L** | 172.380798 | | 194.035156 | 248.295609 | 3258.02734 | 1626.05713 | 1387.58203 | | -3.849226146 | | -3.080167234 | -2.172625866 | | 5.94363E-06 | 2.30336E-05 | 0.000371715 |
| **NAV2** | 339.230652 | | 356.835388 | 369.68457 | 1600.34705 | 828.795898 | 2003.52637 | | -1.846218407 | | -1.181405438 | -2.219391049 | | 0.000989135 | 0.017363273 | 0.000244155 |
| **NBPF15** | 9105.76172 | | 9085.57812 | 12694.3428 | 3125.91138 | 5218.58398 | 4030.27002 | | 1.894378518 | | 0.955565353 | 1.398602229 | | 0.000459809 | 0.038243342 | 0.004151043 |
| **NBPF15** | 695.975952 | | 1079.02478 | 1640.59021 | 256.753479 | 442.294006 | 468.445496 | | 1.860538332 | | 1.324983772 | 2.018306401 | | 0.001407198 | 0.008551368 | 0.000453653 |
| **NCKAP1L** | 405.601868 | | 416.465698 | 945.362549 | 181.970917 | 141.50174 | 193.933701 | | 1.558756625 | | 1.483618398 | 2.669141097 | | 0.006969228 | 0.008799775 | 9.66082E-05 |
| **NCRNA00185** | 209.253693 | | 194.035156 | 142.54007 | 742.84021 | 399.439178 | 655.550537 | | -1.398950613 | | -1.089218419 | -1.763295621 | | 0.009508766 | 0.036684036 | 0.003049315 |
| **NDRG2** | 2416.09668 | | 2512.04541 | 2410.30664 | 16859.7305 | 10766.2607 | 8978.31055 | | -2.452549502 | | -1.98971328 | -2.075305734 | | 6.35746E-05 | 0.000309869 | 0.000239694 |
| **NDRG2** | 1499.80505 | | 1761.46057 | 644.648987 | 9908.69043 | 10424.2305 | 4358.04541 | | -2.355727148 | | -2.456714874 | -2.714357309 | | 9.09606E-05 | 6.33476E-05 | 4.06285E-05 |
| **NDUFA11** | 5838.82324 | | 4328.40381 | 2070.96924 | 14212.4277 | 12188.5557 | 5762.01611 | | -0.930704671 | | -1.354000913 | -1.616760223 | | 0.036480337 | 0.004808899 | 0.001613546 |
| **NDUFB2** | 4738.16748 | | 4975.81836 | 3717.07715 | 14768.3115 | 12731.9219 | 7420.0127 | | -1.302983233 | | -1.213783574 | -1.218188334 | | 0.00616665 | 0.009365682 | 0.009652748 |
| **NDUFS8** | 1846.41028 | | 2202.53564 | 1424.48108 | 7252.66309 | 6488.86523 | 3096.1106 | | -1.617364724 | | -1.447421786 | -1.145786799 | | 0.00155347 | 0.003243851 | 0.015264891 |
| **NDUFV1** | 1459.24487 | | 1543.76257 | 1313.20789 | 5032.8667 | 4916.1748 | 2519.77246 | | -1.446228822 | | -1.569486369 | -0.931316696 | | 0.003518752 | 0.001926396 | 0.041935694 |
| **NEDD4L** | 216.628265 | | 235.681717 | 115.871284 | 736.608337 | 947.657349 | 603.652771 | | -1.336808075 | | -1.994490482 | -1.92722156 | | 0.012176483 | 0.00063282 | 0.004740058 |
| **NEFM** | 190.817245 | | 219.591003 | 205.073776 | 695.477905 | 445.52832 | 662.379211 | | -1.433511823 | | -1.054736015 | -1.265997583 | | 0.008783475 | 0.039334998 | 0.018123498 |
| **NETO2** | 1635.31299 | | 1772.81873 | 967.433228 | 95.9709625 | 144.736069 | 124.281456 | | 4.408253294 | | 3.572369383 | 3.354957661 | | 4.37846E-06 | 6.27528E-05 | 2.75094E-05 |
| **NFASC** | 213.862808 | | 219.591003 | 227.144501 | 1069.39075 | 460.891357 | 1174.52808 | | -1.909278588 | | -1.101120635 | -2.030364149 | | 0.001055321 | 0.032235988 | 0.000685983 |
| **NFKBIE** | 484.878601 | | 486.50766 | 677.755066 | 274.202759 | 168.184921 | 254.025833 | | 1.258018189 | | 1.484348233 | 1.825540032 | | 0.017974651 | 0.007577527 | 0.001662365 |
| **NGFRAP1** | 8851.33887 | | 9286.23828 | 8030.9834 | 27569.8398 | 21475.9199 | 19484.873 | | -1.245941927 | | -1.107443543 | -1.548758495 | | 0.007914696 | 0.015392761 | 0.001937458 |
| **NKG7** | 2570.04102 | | 2598.17798 | 5062.47168 | 1463.24561 | 422.079468 | 1122.63025 | | 1.156342501 | | 2.649231168 | 2.118054241 | | 0.014362326 | 5.12785E-05 | 0.000218681 |
| **NLGN1** | 1142.13794 | | 2562.21045 | 659.362793 | 118.405731 | 92.1782761 | 203.493805 | | 3.589591222 | | 4.736537623 | 2.129704897 | | 1.43804E-05 | 2.65523E-06 | 0.000655498 |
| **NLRC3** | 375.181732 | | 378.605164 | 619.819397 | 108.434723 | 87.3267822 | 152.961792 | | 2.154230261 | | 2.021212483 | 2.473906626 | | 0.001189385 | 0.001709925 | 0.000251089 |
| **NLRP7** | 90.3386002 | | 123.993195 | 98.3986282 | 769.014099 | 273.300507 | 725.202759 | | -2.740721124 | | -1.237933622 | -2.457770427 | | 0.00014607 | 0.028148146 | 0.000347977 |
| **NMB** | 4285.55225 | | 3026.94849 | 2676.99438 | 651.854736 | 855.479065 | 514.88031 | | 3.093492593 | | 1.902012694 | 2.512166621 | | 1.55722E-05 | 0.000536654 | 7.46859E-05 |
| **NNMT** | 60389.5078 | | 62008.9023 | 60235.5938 | 4233.93994 | 1595.33105 | 13576.7246 | | 4.196387231 | | 5.208465909 | 1.832086782 | | 2.411E-06 | 2.62346E-06 | 0.000587839 |
| **NOD1** | 4546.42822 | | 3852.30762 | 8568.03809 | 2942.69409 | 1431.18896 | 3597.3335 | | 0.966532901 | | 1.51940544 | 1.022956836 | | 0.032329794 | 0.002513501 | 0.027054336 |
| **NOL3** | 613.933716 | | 704.205627 | 617.060547 | 147.072388 | 80.858139 | 106.526962 | | 2.412657175 | | 3.074189145 | 2.996908238 | | 0.000297817 | 5.74932E-05 | 7.88364E-05 |
| **NPM1** | 4899.48633 | | 5913.81299 | 6433.61523 | 2135.04224 | 1898.54907 | 1671.65381 | | 1.543964956 | | 1.747118233 | 1.821159824 | | 0.00229072 | 0.000878679 | 0.000652677 |
| **NPNT** | 347.527069 | | 135.351349 | 313.588165 | 1049.44873 | 895.099548 | 1212.76855 | | -1.18688565 | | -2.757395891 | -1.643479351 | | 0.018025346 | 7.30039E-05 | 0.002665097 |
| **NR0B2** | 200.957291 | | 318.974854 | 219.787598 | 1402.17322 | 1345.47937 | 2029.47522 | | -2.402556484 | | -2.051804042 | -2.937542975 | | 0.001360902 | 0.000414702 | 3.6885E-05 |
| **NR1H4** | 848.998474 | | 1132.97595 | 1710.48083 | 240.550598 | 380.841827 | 278.608978 | | 2.232394853 | | 1.605614474 | 2.892878994 | | 0.000346724 | 0.00258307 | 3.59324E-05 |
| **NR3C1** | 1000.17731 | | 1406.51819 | 1966.13342 | 225.594086 | 584.604309 | 770.271851 | | 2.551489689 | | 1.323107301 | 1.456247445 | | 0.00012144 | 0.007767811 | 0.004024639 |
| **NREP** | 4303.06689 | | 5070.46973 | 5602.28467 | 2179.91187 | 1834.67114 | 1585.61292 | | 1.319468119 | | 1.568018283 | 1.712125477 | | 0.006213215 | 0.001941947 | 0.001048489 |
| **NRP1** | 977.131775 | | 1930.88635 | 937.085999 | 321.565063 | 578.135681 | 442.496613 | | 2.006947008 | | 1.790546682 | 1.37369888 | | 0.000638313 | 0.000962498 | 0.007735393 |
| **NT5DC3** | 765.11261 | | 704.205627 | 606.944824 | 499.796844 | 228.828522 | 397.427521 | | 1.023091673 | | 1.6310156 | 0.984241339 | | 0.036082722 | 0.003192687 | 0.047369968 |
| **NUS1** | 5756.78125 | | 6810.16064 | 2501.34839 | 1748.66577 | 1725.51257 | 1320.66113 | | 2.078759489 | | 2.095014687 | 0.928601691 | | 0.000241997 | 0.000221589 | 0.04167464 |
| **NUSAP1** | 1879.59595 | | 2770.44336 | 3158.87183 | 408.811371 | 257.128876 | 454.788177 | | 2.591579817 | | 3.437588795 | 2.930404002 | | 6.95995E-05 | 9.89047E-06 | 2.45103E-05 |
| **OCIAD2** | 8317.60352 | | 6578.26514 | 10903.8555 | 5288.37402 | 2559.96851 | 2710.97461 | | 1.010712468 | | 1.475535646 | 1.80379977 | | 0.025281137 | 0.002856475 | 0.000660752 |
| **ODF3B** | 2147.84619 | | 2961.63892 | 3038.40259 | 425.014282 | 253.085968 | 544.926392 | | 2.727171228 | | 3.560814754 | 2.589359021 | | 0.000399265 | 7.96792E-06 | 5.79307E-05 |
| **OGDHL** | 342.917938 | | 762.889404 | 1137.56177 | 3325.33154 | 2080.47974 | 4653.04297 | | -2.88851205 | | -1.377568442 | -2.078270621 | | 2.93305E-05 | 0.005353271 | 0.000271529 |
| **OLFML2B** | 860.060303 | | 801.696472 | 1496.21094 | 437.478027 | 305.643738 | 338.701111 | | 1.380602332 | | 1.4197865 | 2.413409585 | | 0.007665722 | 0.006696881 | 0.000132909 |
| **OMP** | 298.670471 | | 353.049316 | 366.006104 | 7007.12695 | 722.871704 | 5441.06934 | | -4.147989928 | | -0.999113887 | -3.78360026 | | 3.21826E-06 | 0.039609995 | 5.34864E-06 |
| **OR11H6** | 4079.06421 | | 6495.91846 | 6340.73389 | 2578.7522 | 1847.6084 | 2495.18921 | | 0.999871741 | | 1.925505276 | 1.174099253 | | 0.02794561 | 0.000421403 | 0.011986962 |
| **OR9G4** | 10381.5645 | | 5444.34229 | 14577.7109 | 6619.50391 | 2034.39062 | 5281.27881 | | 0.997500861 | | 1.525074742 | 1.187611823 | | 0.026663473 | 0.002330463 | 0.010689319 |
| **ORAI3** | 3981.35107 | | 5147.13721 | 4055.49487 | 1842.14392 | 1878.33447 | 1126.72754 | | 1.451804768 | | 1.556482865 | 1.819275833 | | 0.003413162 | 0.002039899 | 0.000712865 |
| **OSGEP** | 1110.79602 | | 1190.71326 | 3656.38281 | 644.376465 | 404.29068 | 667.842102 | | 1.169298389 | | 1.591686689 | 2.507862246 | | 0.016821263 | 0.002663668 | 6.79366E-05 |
| **OSMR** | 1848.25403 | | 1632.73486 | 1012.49432 | 233.072342 | 813.432861 | 494.394348 | | 3.384079443 | | 1.075517549 | 1.2991209 | | 1.32967E-05 | 0.022481764 | 0.010222454 |
| **OXCT1** | 431.412903 | | 530.047241 | 275.884003 | 3358.98364 | 5406.1748 | 1358.90161 | | -2.575807741 | | -3.257936904 | -1.996815622 | | 6.31924E-05 | 1.04695E-05 | 0.000673327 |
| **P2RX4** | 2164.43896 | | 2177.92627 | 4090.44019 | 974.666138 | 769.76947 | 1546.00671 | | 1.514077197 | | 1.570825957 | 1.331702288 | | 0.003594471 | 0.002286475 | 0.006522531 |
| **P4HB** | 22295.1973 | | 19425.2852 | 33185.168 | 12908.7178 | 9085.21973 | 6207.24414 | | 1.169305119 | | 1.205946345 | 2.090867924 | | 0.011427337 | 0.009597569 | 0.001052722 |
| **PAG1** | 797.376404 | | 890.668701 | 1015.25317 | 244.289734 | 339.604156 | 243.099991 | | 2.122550677 | | 1.422286776 | 2.417411213 | | 0.000516984 | 0.006239331 | 0.000176662 |
| **PALM** | 853.607544 | | 807.375549 | 1041.00232 | 5120.11328 | 1786.15625 | 2771.06665 | | -2.229756867 | | -1.076806247 | -1.385322014 | | 0.000155564 | 0.021802517 | 0.005311788 |
| **PALM3** | 194.504532 | | 386.177277 | 305.311646 | 1031.99951 | 971.914795 | 1342.51282 | | -1.99222197 | | -1.295640317 | -1.840267249 | | 0.000843688 | 0.00992607 | 0.001181176 |
| **PANK1** | 393.618164 | | 818.733704 | 306.231262 | 1201.50659 | 1859.73706 | 1324.75842 | | -1.211155786 | | -1.114829329 | -1.815154612 | | 0.015267068 | 0.018183926 | 0.001307859 |
| **PAQR5** | 1207.5874 | | 1495.49048 | 236.340637 | 4944.37402 | 6445.20215 | 3691.56885 | | -1.692434739 | | -1.99407844 | -3.771646867 | | 0.001198687 | 0.000321444 | 6.38488E-06 |
| **PAQR5** | 2029.85303 | | 1916.68872 | 670.398132 | 5380.60596 | 7193.13965 | 3570.01904 | | -1.068267294 | | -1.793654329 | -2.352334771 | | 0.020255243 | 0.000702592 | 0.000119828 |
| **PBX1** | 596.419128 | | 608.607849 | 860.758118 | 2907.79565 | 2399.86938 | 2476.06909 | | -1.91237495 | | -1.919783209 | -1.460650621 | | 0.000732569 | 0.000525657 | 0.003929394 |
| **PCDH9** | 125.367851 | | 104.116425 | 113.112442 | 835.072021 | 760.875061 | 884.993225 | | -2.370957092 | | -2.918040935 | -2.571206565 | | 0.000323549 | 6.05904E-05 | 0.000199532 |
| **PDE6B** | 2656.69214 | | 2112.61694 | 1503.56787 | 597.01416 | 384.884735 | 800.317932 | | 2.536724456 | | 2.479873826 | 1.046141622 | | 6.88016E-05 | 8.8972E-05 | 0.028285205 |
| **PDE7A** | 1763.44629 | | 2146.69141 | 1610.24304 | 578.318542 | 502.937592 | 811.243774 | | 1.993101098 | | 2.132160719 | 1.114352243 | | 0.000468293 | 0.000261486 | 0.0196325 |
| **PDE7B** | 334.621552 | | 312.349274 | 192.199188 | 1567.94116 | 1199.1261 | 659.647705 | | -1.836077517 | | -1.913654045 | -1.350969302 | | 0.001040197 | 0.000726998 | 0.013200591 |
| **PDGFD** | 1853.78491 | | 2408.87549 | 990.423584 | 486.0867 | 394.587708 | 352.358398 | | 2.318875743 | | 2.634773831 | 1.808606474 | | 0.000153239 | 5.49568E-05 | 0.001324013 |
| **PDIA4** | 943.946167 | | 998.571167 | 1953.25879 | 507.275085 | 450.379822 | 456.153931 | | 1.294230341 | | 1.188091088 | 2.288256858 | | 0.010506693 | 0.015916161 | 0.000167661 |
| **PDLIM1** | 16315.335 | | 12885.8271 | 24740.3574 | 4570.46143 | 6443.58496 | 6306.94238 | | 2.208586455 | | 1.146716417 | 1.685997682 | | 0.000528075 | 0.025560971 | 0.001132739 |
| **PECAM1** | 3713.10083 | | 4627.50195 | 2291.67651 | 974.666138 | 1440.89197 | 968.302795 | | 2.290643975 | | 1.786359129 | 1.299787674 | | 0.000128747 | 0.000774756 | 0.00777587 |
| **PFKFB4** | 1384.57727 | | 1721.70703 | 1194.57776 | 361.449097 | 276.534821 | 539.46344 | | 2.331964094 | | 2.649215724 | 1.376852958 | | 0.000174457 | 6.30228E-05 | 0.006817788 |
| **PFKP** | 16024.0391 | | 12121.9912 | 7821.31152 | 2435.41895 | 3793.05518 | 2795.6499 | | 3.09068096 | | 1.799995156 | 1.289166929 | | 1.2675E-05 | 0.000783746 | 0.006824144 |
| **PFN2** | 703.350525 | | 526.261169 | 846.044312 | 1853.36133 | 2069.15967 | 1785.00952 | | -1.029678618 | | -1.92983737 | -0.972185766 | | 0.028907964 | 0.00053182 | 0.037650652 |
| **PGF** | 1362.45349 | | 1246.5575 | 10610.499 | 134.608627 | 82.475296 | 163.887634 | | 3.657930159 | | 3.863799145 | 6.160806487 | | 1.44913E-05 | 9.46721E-06 | 9.4543E-07 |
| **PGM5** | 591.809998 | | 254.611984 | 217.948364 | 1584.14404 | 1937.36096 | 1205.93982 | | -1.044513944 | | -2.920595162 | -2.127760712 | | 0.028271664 | 2.9117E-05 | 0.000485233 |
| **PHF21B** | 94.9477081 | | 117.367607 | 259.330963 | 1364.78186 | 461.699951 | 1164.9679 | | -3.522898101 | | -2.058461475 | -1.837931611 | | 1.7651E-05 | 0.001005449 | 0.001331773 |
| **PHKA2** | 2767.31104 | | 3888.27515 | 7581.29248 | 1166.60803 | 649.290833 | 1129.45898 | | 1.60025128 | | 2.673689209 | 2.668153066 | | 0.00190512 | 4.17434E-05 | 3.71844E-05 |
| **PHYHD1** | 407.445526 | | 220.537521 | 281.401703 | 3351.50537 | 2690.15015 | 2191.99707 | | -2.654064803 | | -3.611458611 | -2.722478374 | | 5.14243E-05 | 7.70776E-06 | 5.6645E-05 |
| **PHYHD1** | 124.44603 | | 97.4908295 | 83.6848145 | 985.883545 | 739.043335 | 531.269104 | | -2.629702001 | | -2.970744009 | -2.191493903 | | 0.000136912 | 5.62271E-05 | 0.001115297 |
| **PID1** | 427.725616 | | 383.337738 | 169.208862 | 2063.99902 | 3778.50073 | 1806.86121 | | -1.884446126 | | -3.252355616 | -3.121567344 | | 0.000744704 | 1.15475E-05 | 2.81408E-05 |
| **PIK3CD** | 794.610962 | | 900.133789 | 944.442932 | 312.840424 | 372.756012 | 538.097717 | | 1.758627714 | | 1.305961488 | 1.071357418 | | 0.001818334 | 0.010082212 | 0.027865168 |
| **PIK3R5** | 1728.41711 | | 1185.98071 | 3726.27344 | 373.912842 | 211.848312 | 367.381439 | | 2.601117787 | | 2.483493704 | 3.488195811 | | 7.11686E-05 | 0.000126142 | 8.23348E-06 |
| **PKHD1** | 398.227295 | | 505.437897 | 248.295609 | 1130.46313 | 1233.08655 | 1255.10608 | | -1.103668036 | | -1.23630535 | -2.014073507 | | 0.024720166 | 0.011742942 | 0.000678348 |
| **PLA1A** | 869.278564 | | 1433.96704 | 2311.90796 | 265.478119 | 283.003479 | 285.437622 | | 2.122155859 | | 2.35859838 | 3.251590533 | | 0.000478915 | 0.000157035 | 1.48047E-05 |
| **PLA2G16** | 1431.59021 | | 1594.87427 | 1112.73218 | 339.014313 | 477.062988 | 517.611755 | | 2.472957206 | | 1.775742639 | 1.349771584 | | 0.000113346 | 0.001106045 | 0.007900625 |
| **PLAC8** | 581.669983 | | 637.003235 | 503.94812 | 274.202759 | 146.353226 | 270.414612 | | 1.513368942 | | 2.087322692 | 1.350306883 | | 0.005877338 | 0.00072425 | 0.012470526 |
| **PLAT** | 753.128906 | | 486.50766 | 792.706726 | 4390.9834 | 3147.80713 | 2936.32007 | | -2.181703679 | | -2.64560832 | -1.832735833 | | 0.002256961 | 5.22296E-05 | 0.001062333 |
| **PLAU** | 388.08725 | | 285.846893 | 262.089813 | 1891.99902 | 5927.70996 | 824.901123 | | -1.896997493 | | -4.308984814 | -1.282401583 | | 0.000745484 | 6.38607E-06 | 0.014480392 |
| **PLEKHJ1** | 1726.57336 | | 1668.70227 | 1356.42969 | 4748.69336 | 3619.21021 | 2859.83911 | | -1.120355476 | | -1.038539519 | -1.086057508 | | 0.027102578 | 0.023424259 | 0.020434899 |
| **PLEKHO1** | 8521.32617 | | 7347.77979 | 16323.1367 | 4518.11377 | 3633.76465 | 5539.40186 | | 1.271590853 | | 1.151306112 | 1.287711213 | | 0.00775898 | 0.012982704 | 0.007422904 |
| **PLEKHO1** | 601.950073 | | 530.047241 | 744.886841 | 368.927338 | 266.831848 | 288.169098 | | 1.130521114 | | 0.98792636 | 1.753370354 | | 0.025978362 | 0.048138082 | 0.001993438 |
| **PLIN2** | 43417.8359 | | 55273.5156 | 46517.7227 | 8161.271 | 6649.77295 | 7562.04883 | | 2.800255872 | | 3.035935489 | 2.30507874 | | 2.31189E-05 | 1.34268E-05 | 9.51374E-05 |
| **PLIN5** | 360.432556 | | 430.663391 | 655.684326 | 2925.24487 | 951.700256 | 3553.63013 | | -2.632440967 | | -1.104562611 | -2.373517458 | | 5.78779E-05 | 0.022882564 | 0.00011256 |
| **PLOD3** | 1438.04297 | | 1527.67188 | 1814.39722 | 754.057556 | 596.733032 | 685.596619 | | 1.306735776 | | 1.412473481 | 1.538457422 | | 0.008395267 | 0.005101248 | 0.00344915 |
| **PLVAP** | 8029.99512 | | 10296.168 | 10278.5186 | 5103.91016 | 4143.1709 | 2443.2915 | | 1.014553318 | | 1.454348501 | 1.882458576 | | 0.024867093 | 0.003038844 | 0.000482276 |
| **PLXNB1** | 166.849854 | | 265.023621 | 113.112442 | 898.637207 | 770.578003 | 827.632568 | | -2.017625272 | | -1.516032666 | -2.466146323 | | 0.000860543 | 0.004465114 | 0.00028302 |
| **PMEPA1** | 1178.08911 | | 1559.85327 | 1606.56458 | 613.217041 | 185.165131 | 370.112915 | | 1.326805786 | | 3.05497975 | 2.365359367 | | 0.008291195 | 2.70813E-05 | 0.000146737 |
| **PMM2** | 2418.86206 | | 2322.74268 | 4867.51367 | 1607.8252 | 600.77594 | 1001.08032 | | 0.932412839 | | 2.00626093 | 2.245823568 | | 0.042249974 | 0.000395137 | 0.000142049 |
| **PNCK** | 2557.1355 | | 757.210327 | 13217.6025 | 408.811371 | 109.967064 | 645.990417 | | 3.035386427 | | 2.734891934 | 4.332492472 | | 2.11048E-05 | 0.000104685 | 2.31232E-06 |
| **PNKD** | 553.093445 | | 588.731018 | 290.597809 | 2212.31763 | 3031.37158 | 1006.54321 | | -1.623374886 | | -2.301535093 | -1.454466313 | | 0.002029683 | 0.000127916 | 0.00638248 |
| **POT1** | 636.979309 | | 703.259094 | 1488.854 | 343.999817 | 295.940765 | 308.655029 | | 1.311584356 | | 1.277039583 | 2.554592187 | | 0.012130656 | 0.012953351 | 8.98298E-05 |
| **POU5F1** | 464.598511 | | 573.586853 | 1064.91223 | 142.086884 | 192.442368 | 259.48877 | | 2.068119832 | | 1.553733899 | 2.380515455 | | 0.001131059 | 0.005060168 | 0.000186794 |
| **PP7080** | 795.532776 | | 824.412781 | 1628.63525 | 14451.7314 | 10410.4854 | 7038.97412 | | -3.83002786 | | -3.536953478 | -2.229423827 | | 4.01323E-06 | 9.13698E-06 | 0.000146017 |
| **PPAPDC1A** | 371.494446 | | 308.563232 | 500.269653 | 1662.66577 | 1032.55835 | 1877.87915 | | -1.772065142 | | -1.713866908 | -1.725790411 | | 0.001282776 | 0.001697706 | 0.00148289 |
| **PPARGC1A** | 589.96637 | | 612.39386 | 902.140686 | 1597.85425 | 4245.86084 | 2462.41162 | | -1.06137741 | | -2.69994234 | -1.390173269 | | 0.026195724 | 3.71525E-05 | 0.005361373 |
| **PPM1F** | 2803.26196 | | 1960.22827 | 4747.04395 | 1888.25989 | 945.231628 | 1626.58472 | | 0.912098787 | | 1.120970767 | 1.448140239 | | 0.043498341 | 0.017589094 | 0.00344926 |
| **PPP1R18** | 4116.85889 | | 4618.98291 | 11119.9648 | 1562.95569 | 2076.43701 | 2028.1095 | | 1.738003593 | | 1.251742406 | 2.283068999 | | 0.000973069 | 0.008388792 | 0.00011075 |
| **PPP1R3B** | 867.434875 | | 1288.2041 | 532.456116 | 145.826004 | 452.805542 | 219.882568 | | 2.906600753 | | 1.543586675 | 1.737827368 | | 6.15878E-05 | 0.003127245 | 0.00283752 |
| **PPP1R3C** | 2012.33838 | | 2954.06689 | 1083.30457 | 312.840424 | 780.281006 | 491.662903 | | 3.079384926 | | 1.998114519 | 1.397088898 | | 2.18061E-05 | 0.000375858 | 0.006530602 |
| **PRDX2** | 1197.44739 | | 1521.04626 | 1343.55518 | 3709.21533 | 3573.12109 | 2779.26123 | | -1.287984954 | | -1.152381364 | -1.054026471 | | 0.007662085 | 0.013749494 | 0.023710746 |
| **PRDX2** | 554.015259 | | 487.454163 | 144.379303 | 1609.07166 | 2390.9751 | 749.78595 | | -1.15972438 | | -2.255169082 | -1.960968091 | | 0.016982159 | 0.000160517 | 0.001362506 |
| **PRELID2** | 548.484375 | | 612.39386 | 226.224884 | 112.173851 | 130.181595 | 135.207306 | | 2.642032201 | | 2.186244665 | 1.329648111 | | 0.000196245 | 0.000561021 | 0.027567398 |
| **PRELP** | 212.019165 | | 130.61879 | 298.874329 | 1246.3761 | 730.148987 | 1629.31628 | | -2.150086468 | | -2.516694339 | -2.175087827 | | 0.00041393 | 0.000161893 | 0.000324532 |
| **PRKAR2B** | 201.879105 | | 232.842178 | 170.128479 | 785.21698 | 2459.70459 | 502.588745 | | -1.532628531 | | -3.399960817 | -1.086424508 | | 0.005452336 | 1.0454E-05 | 0.043166123 |
| **PRKCDBP** | 11914.5547 | | 12215.6963 | 25320.6348 | 5421.73633 | 2830.84326 | 4129.96826 | | 1.472770443 | | 2.209138882 | 2.359794314 | | 0.002845713 | 0.000139226 | 8.18578E-05 |
| **PRKCQ-AS1** | 1179.01086 | | 1154.74573 | 1015.25317 | 3737.88208 | 2656.99829 | 2717.80322 | | -1.321430817 | | -1.134385822 | -1.388117015 | | 0.00656121 | 0.015573571 | 0.005250796 |
| **PROM2** | 101.400467 | | 121.153656 | 208.752228 | 2196.11475 | 1296.15588 | 1195.01404 | | -4.119660525 | | -3.465152693 | -2.171923946 | | 5.66597E-06 | 1.37963E-05 | 0.000426452 |
| **PROS1** | 5355.78809 | | 8020.75049 | 8794.2627 | 1732.46289 | 2469.40747 | 2527.9668 | | 1.982304031 | | 1.813035391 | 1.609903202 | | 0.000350345 | 0.000644772 | 0.001546003 |
| **PRR13** | 2818.93311 | | 2433.48486 | 1318.72559 | 31201.7812 | 18527.834 | 35196.2344 | | -3.092137662 | | -2.864135191 | -4.873556427 | | 1.23297E-05 | 2.04547E-05 | 1.47389E-06 |
| **PSMB8** | 1673.10767 | | 1712.24194 | 2596.98804 | 651.854736 | 746.320618 | 439.765137 | | 1.74012281 | | 1.267587544 | 2.723462644 | | 0.001259163 | 0.009730501 | 9.11002E-05 |
| **PSMB9** | 10572.3818 | | 8391.78418 | 16711.2148 | 2901.56372 | 2826.80029 | 2609.91064 | | 2.203955777 | | 1.683348607 | 2.489827007 | | 0.000143864 | 0.001108106 | 5.60604E-05 |
| **PTAFR** | 1319.12793 | | 1474.66711 | 2410.30664 | 730.376404 | 313.729553 | 920.502197 | | 1.230052537 | | 2.252880174 | 1.44623902 | | 0.012131556 | 0.000213057 | 0.003969629 |
| **PTAFR** | 362.276215 | | 349.263275 | 454.289001 | 236.811462 | 121.287201 | 240.36853 | | 1.057179957 | | 1.434179587 | 1.39603295 | | 0.046455935 | 0.012490278 | 0.011368308 |
| **PTGIS** | 247.048416 | | 196.874695 | 139.781235 | 730.376404 | 768.152283 | 747.054443 | | -1.135834013 | | -1.964748748 | -2.000834278 | | 0.026961945 | 0.000824701 | 0.001207824 |
| **PTGR1** | 1503.49243 | | 1289.15063 | 288.758606 | 3727.91113 | 9307.58008 | 2052.69263 | | -0.968334127 | | -2.734468405 | -2.585310468 | | 0.041779425 | 2.97689E-05 | 8.35833E-05 |
| **PTPN1** | 1268.42761 | | 2051.09351 | 1951.41956 | 449.941803 | 574.901367 | 1062.53821 | | 1.886402585 | | 1.88558761 | 0.942119806 | | 0.000832336 | 0.000649841 | 0.040755831 |
| **PTPRC** | 534.656982 | | 560.335632 | 688.790405 | 72.2898178 | 181.930801 | 127.012917 | | 3.240081451 | | 1.594275869 | 2.882559507 | | 6.41923E-05 | 0.00444324 | 8.52881E-05 |
| **PVALB** | 161.318924 | | 173.211868 | 182.08345 | 4662.69336 | 1427.14612 | 1126.72754 | | -4.475362254 | | -3.061704901 | -2.272680211 | | 2.73775E-06 | 2.599E-05 | 0.000333258 |
| **PXDN** | 903.385986 | | 851.861633 | 583.954468 | 317.825928 | 271.68335 | 330.506714 | | 1.914531393 | | 1.671074841 | 1.227867084 | | 0.000935583 | 0.002376951 | 0.018250547 |
| **PXMP2** | 143.804306 | | 194.981659 | 72.6494522 | 949.738647 | 780.281006 | 297.729187 | | -2.340274648 | | -2.002167627 | -1.475372489 | | 0.00030407 | 0.000715847 | 0.02047535 |
| **RAB24** | 1991.13647 | | 1922.3678 | 1920.15271 | 1126.724 | 708.317261 | 908.210632 | | 1.177555679 | | 1.509888654 | 1.167088682 | | 0.013690738 | 0.003089856 | 0.014825015 |
| **RAB40B** | 441.552948 | | 494.079742 | 211.511078 | 1212.724 | 1327.69055 | 819.438171 | | -1.061209303 | | -1.379723895 | -1.56260074 | | 0.028771322 | 0.006078589 | 0.004954158 |
| **RAB42** | 2138.62793 | | 2774.22949 | 8242.49414 | 230.57959 | 96.2211838 | 285.437622 | | 3.610071008 | | 4.790519386 | 4.965994225 | | 8.67178E-06 | 2.43237E-06 | 1.56492E-06 |
| **RAC2** | 1227.86743 | | 838.610474 | 1533.91504 | 378.898346 | 231.254272 | 267.683136 | | 2.090614285 | | 1.867740073 | 2.811874056 | | 0.000408126 | 0.001161604 | 4.63426E-05 |
| **RALGPS1** | 247.048416 | | 181.730484 | 308.990082 | 1718.75269 | 1835.47961 | 1525.52075 | | -2.402721023 | | -3.357182821 | -2.025960973 | | 0.000149699 | 1.29821E-05 | 0.000557841 |
| **RAP1GAP** | 105.087753 | | 187.409561 | 72.6494522 | 1212.724 | 974.340515 | 684.230896 | | -3.20034733 | | -2.384187012 | -2.802023045 | | 3.33395E-05 | 0.000178716 | 0.000175045 |
| **RAPGEF3** | 2195.78101 | | 1866.52356 | 3261.86865 | 26318.4785 | 7619.26221 | 23467.3438 | | -3.202576241 | | -1.915946913 | -3.065439027 | | 1.00278E-05 | 0.00042611 | 1.33081E-05 |
| **RAPGEF6** | 2799.57471 | | 2989.08789 | 3710.63989 | 1540.52087 | 1662.44324 | 1536.44653 | | 1.205108498 | | 0.912997123 | 1.212733569 | | 0.011272389 | 0.04272788 | 0.010614589 |
| **RASAL3** | 2201.31201 | | 1896.81189 | 4193.43701 | 1299.97034 | 532.046509 | 1443.5769 | | 1.10893193 | | 1.876953915 | 1.472882216 | | 0.018352648 | 0.000693108 | 0.003136925 |
| **RASSF2** | 6131.96289 | | 3627.98413 | 6135.66016 | 929.796631 | 1296.96448 | 726.568481 | | 3.108743326 | | 1.572597681 | 3.067630912 | | 1.38582E-05 | 0.002062375 | 1.51968E-05 |
| **RASSF5** | 843.467529 | | 835.770935 | 1452.98914 | 269.217255 | 236.914337 | 305.923584 | | 2.059830462 | | 1.829967561 | 2.536829 | | 0.000602566 | 0.001333759 | 9.57605E-05 |
| **RBM3** | 543.875244 | | 1080.91772 | 774.314453 | 186.956421 | 517.492065 | 366.015717 | | 1.938205742 | | 1.114405308 | 1.426515806 | | 0.001393138 | 0.021255622 | 0.006914517 |
| **RGL1** | 2061.19507 | | 3473.70239 | 2127.06567 | 962.202393 | 1187.80603 | 1035.22351 | | 1.462805678 | | 1.635480255 | 1.095942975 | | 0.003757402 | 0.001541751 | 0.020034341 |
| **RGS1** | 3449.45947 | | 1850.43274 | 6181.64111 | 145.826004 | 279.769135 | 473.908417 | | 4.880959742 | | 2.737623177 | 3.76413622 | | 4.46563E-06 | 4.85141E-05 | 4.80728E-06 |
| **RGS10** | 2249.24683 | | 2194.01709 | 4818.77393 | 842.550293 | 1155.46277 | 868.604492 | | 1.786021735 | | 0.992896121 | 2.455071852 | | 0.001071688 | 0.030902946 | 7.37129E-05 |
| **RGS19** | 1729.33887 | | 1790.80249 | 2455.36768 | 878.69519 | 679.208313 | 523.074707 | | 1.344938248 | | 1.463888354 | 2.371940568 | | 0.006675754 | 0.003861086 | 0.000302176 |
| **RGS5** | 7755.29199 | | 10529.0098 | 7423.11914 | 793.941589 | 3881.19043 | 947.816833 | | 3.685355068 | | 1.575243586 | 2.913703693 | | 5.08479E-06 | 0.001750472 | 2.04493E-05 |
| **RHOB** | 781.705444 | | 1385.69495 | 1152.27551 | 206.898438 | 326.66687 | 460.251099 | | 2.317676441 | | 2.107630406 | 1.583552851 | | 0.000292641 | 0.000354792 | 0.002909656 |
| **RHOBTB1** | 2538.69897 | | 2936.08325 | 3222.3252 | 1311.18774 | 592.690125 | 1332.95276 | | 1.301705922 | | 2.368954892 | 1.250587953 | | 0.007359708 | 0.000107763 | 0.009097226 |
| **RHOBTB3** | 3026.34302 | | 793.177856 | 4513.4624 | 8505.27051 | 13023.0117 | 7878.89795 | | -1.122480337 | | -3.929382643 | -1.050922388 | | 0.015080138 | 3.44359E-06 | 0.021227295 |
| **RIMKLA** | 1748.69714 | | 1075.23865 | 834.089294 | 143.333252 | 79.2409744 | 135.207306 | | 3.925845952 | | 3.711662446 | 3.035482856 | | 6.62828E-06 | 1.3151E-05 | 4.99335E-05 |
| **RIPK2** | 1725.65161 | | 1984.83765 | 4490.47217 | 978.405273 | 1048.72998 | 803.049438 | | 1.181896682 | | 0.988580461 | 2.484859126 | | 0.013893986 | 0.032027707 | 6.86748E-05 |
| **RNASET2** | 30103.0332 | | 28377.4043 | 36688.8945 | 5446.66357 | 5792.67676 | 3351.5022 | | 2.835039339 | | 2.355877232 | 3.188828146 | | 2.15181E-05 | 8.16673E-05 | 0.000896777 |
| **RNASET2** | 11390.96 | | 8690.88184 | 7922.46924 | 1346.0863 | 1951.10681 | 849.484253 | | 3.422302611 | | 2.266177816 | 3.176021157 | | 7.14465E-06 | 0.000118805 | 1.16936E-05 |
| **RNF126** | 771.565369 | | 658.77301 | 964.674438 | 178.231796 | 336.369843 | 305.923584 | | 2.488711065 | | 0.997819843 | 1.998398059 | | 0.000185984 | 0.04191849 | 0.000677937 |
| **RNF213** | 433.256531 | | 489.347198 | 535.214966 | 183.2173 | 233.680008 | 189.836517 | | 1.644113654 | | 1.045783339 | 1.968217552 | | 0.004826197 | 0.040130563 | 0.001297199 |
| **RPL13AP3** | 2529.48071 | | 3032.62744 | 6020.7085 | 533.448975 | 668.696777 | 360.552795 | | 2.631198503 | | 2.255297388 | 4.161848879 | | 5.43417E-05 | 0.000152541 | 3.07515E-06 |
| **RRM2** | 261.797577 | | 281.114349 | 709.021912 | 140.8405 | 71.9637375 | 132.47583 | | 1.26346246 | | 1.861693875 | 2.857571808 | | 0.029961085 | 0.004205977 | 8.66394E-05 |
| **RTP4** | 3555.46899 | | 1727.38611 | 1123.76758 | 442.463531 | 792.409729 | 333.23819 | | 3.393791066 | | 1.193295679 | 2.06368636 | | 9.57429E-06 | 0.013034483 | 0.000485121 |
| **RUNX1** | 1435.27747 | | 919.064087 | 2494.91113 | 127.130371 | 178.696472 | 200.76236 | | 3.815190734 | | 2.350188626 | 3.891237906 | | 8.75719E-06 | 0.00022764 | 5.59925E-06 |
| **RUNX3** | 809.360107 | | 659.719543 | 684.192322 | 104.695595 | 153.630463 | 131.110107 | | 3.28626431 | | 2.075047736 | 2.82724577 | | 3.30658E-05 | 0.000724306 | 9.66347E-05 |
| **S100A2** | 1159.65259 | | 1259.80872 | 3056.79492 | 23428.1328 | 8742.38184 | 34285.293 | | -3.950052909 | | -2.675788432 | -3.730943913 | | 3.31273E-06 | 3.48977E-05 | 4.25496E-06 |
| **S100A8** | 629.604736 | | 1263.59473 | 779.832153 | 403.825867 | 264.406097 | 345.529755 | | 1.061582439 | | 2.271824841 | 1.52712377 | | 0.033528388 | 0.000222102 | 0.004594943 |
| **SAMD3** | 293.139526 | | 214.858444 | 255.652512 | 100.956467 | 84.9010391 | 159.790436 | | 1.905583667 | | 1.214523115 | 1.249421521 | | 0.009657707 | 0.04198676 | 0.031593569 |
| **SASH3** | 495.018646 | | 465.684357 | 606.944824 | 292.898407 | 133.415924 | 244.465729 | | 1.191299615 | | 1.732467247 | 1.743119973 | | 0.022927744 | 0.003279385 | 0.002462464 |
| **SAT2** | 418.507385 | | 498.812317 | 85.5240402 | 1888.25989 | 1220.14929 | 822.169617 | | -1.786562677 | | -1.241073645 | -2.859118999 | | 0.001132263 | 0.011538552 | 0.00011698 |
| **SBK1** | 203.722763 | | 171.318848 | 171.04808 | 730.376404 | 342.838501 | 591.361206 | | -1.412269837 | | -1.063322186 | -1.340130255 | | 0.009151641 | 0.044452995 | 0.015096645 |
| **SCARB1** | 2198.54639 | | 2162.78198 | 3671.09644 | 496.057709 | 174.653564 | 411.084808 | | 2.535141228 | | 3.604538038 | 3.28987013 | | 7.4908E-05 | 8.7501E-06 | 1.14005E-05 |
| **SCD** | 3140.64893 | | 5934.63623 | 2983.22583 | 259.246246 | 254.703125 | 243.099991 | | 3.99334969 | | 4.598712553 | 3.836056628 | | 4.46909E-06 | 2.12615E-06 | 5.50359E-06 |
| **SCGN** | 9655.16797 | | 16000.8008 | 788.108643 | 135.854996 | 520.726379 | 232.174149 | | 6.473694177 | | 4.999752057 | 2.156061779 | | 8.93063E-07 | 1.41324E-06 | 0.000479012 |
| **SCIN** | 315.263275 | | 170.37233 | 408.308319 | 1303.70947 | 6792.89209 | 1711.26001 | | -1.649307847 | | -5.286954034 | -1.842287862 | | 0.002406111 | 1.34186E-06 | 0.000997781 |
| **SCMH1** | 528.204224 | | 542.351929 | 549.928772 | 228.086838 | 244.191574 | 286.803345 | | 1.644879823 | | 1.143779151 | 1.372600761 | | 0.003922587 | 0.025805159 | 0.01073238 |
| **SCNN1A** | 511.61145 | | 291.52597 | 366.006104 | 6750.37305 | 5191.90088 | 7346.26318 | | -3.332954438 | | -4.094229106 | -4.232088624 | | 9.56877E-06 | 3.30572E-06 | 2.99152E-06 |
| **SCO2** | 3483.56689 | | 2352.08472 | 9904.23633 | 2383.07129 | 1123.11951 | 1481.81738 | | 0.887163661 | | 1.135205454 | 2.614830835 | | 0.047630049 | 0.015910387 | 4.23158E-05 |
| **SDC1** | 3443.00684 | | 4276.34521 | 2683.43188 | 12690.6016 | 9783.83398 | 4875.65723 | | -1.538838975 | | -1.046487435 | -1.023032374 | | 0.002080971 | 0.021118548 | 0.027126793 |
| **SEC16B** | 477.504028 | | 591.570557 | 271.28595 | 134.608627 | 184.356552 | 170.716278 | | 2.184562633 | | 1.65951853 | 1.232483312 | | 0.000774479 | 0.003340091 | 0.031650983 |
| **SELENBP1** | 742.988892 | | 708.938171 | 800.063599 | 4328.66455 | 3179.3418 | 1810.95837 | | -2.179885026 | | -2.08393507 | -1.067220655 | | 0.000192706 | 0.000263953 | 0.024723329 |
| **SEMA5B** | 2280.58862 | | 2985.30176 | 2173.96606 | 335.275177 | 133.415924 | 258.123016 | | 3.15924917 | | 4.444234692 | 3.323719212 | | 1.74778E-05 | 3.01203E-06 | 1.34864E-05 |
| **SEPSECS** | 4180.46484 | | 4456.18311 | 6193.59619 | 2378.08569 | 2304.45679 | 2403.6853 | | 1.152086736 | | 1.048762776 | 1.199573937 | | 0.013647106 | 0.02192199 | 0.010644875 |
| **SERPINA1** | 1940.43616 | | 2987.19482 | 1925.67041 | 452.43454 | 516.683472 | 279.974701 | | 2.489482483 | | 2.580583956 | 3.040072552 | | 9.04617E-05 | 5.82047E-05 | 2.44802E-05 |
| **SERPINH1** | 12109.9814 | | 10395.5518 | 33497.8359 | 4129.24414 | 3869.87036 | 6521.3623 | | 1.892708369 | | 1.561007613 | 2.031257934 | | 0.000451761 | 0.001867505 | 0.000252227 |
| **SERTAD2** | 6033.32764 | | 6611.39307 | 6599.14551 | 2734.54932 | 3611.93286 | 2736.92358 | | 1.503310918 | | 1.006358397 | 1.085773573 | | 0.002552411 | 0.026026029 | 0.01817226 |
| **SEZ6L2** | 1225.10205 | | 2269.73804 | 2058.09473 | 558.376526 | 143.118896 | 714.276917 | | 1.521250525 | | 3.945514291 | 1.637117337 | | 0.003557323 | 5.55319E-06 | 0.001821366 |
| **SFMBT2** | 1430.66846 | | 1492.65088 | 3466.02271 | 580.811279 | 800.495544 | 543.560669 | | 1.686320029 | | 0.971609086 | 2.76802802 | | 0.001676947 | 0.036455795 | 3.46423E-05 |
| **SFRP1** | 167.771683 | | 216.751465 | 170.128479 | 4549.27295 | 8820.00586 | 2532.06396 | | -4.376270368 | | -5.297737245 | -3.640668325 | | 2.9978E-06 | 1.28008E-06 | 9.45671E-06 |
| **SGK2** | 552.171631 | | 978.694397 | 1001.45892 | 2184.89746 | 3037.84009 | 2471.97192 | | -1.60764449 | | -1.558451798 | -1.258592693 | | 0.002177873 | 0.002191491 | 0.009631483 |
| **SH3BP2** | 1980.99646 | | 2686.20361 | 3839.38574 | 348.985321 | 366.287354 | 527.171875 | | 2.898021511 | | 2.897672829 | 2.952400873 | | 3.21428E-05 | 2.77825E-05 | 2.18582E-05 |
| **SH3GL1** | 8928.77246 | | 6309.45508 | 13464.0596 | 5477.82324 | 2598.78052 | 5169.28906 | | 1.055638905 | | 1.392005684 | 1.099195461 | | 0.020351291 | 0.004213973 | 0.022243088 |
| **SH3PXD2B** | 463.676697 | | 725.975403 | 783.510559 | 226.840454 | 177.079315 | 294.997742 | | 1.469028892 | | 2.023579403 | 1.78218326 | | 0.008403575 | 0.000775049 | 0.001722691 |
| **SHC1** | 6325.54541 | | 5549.40527 | 13479.6924 | 3810.17188 | 3235.94263 | 3014.16675 | | 1.096179686 | | 0.896261472 | 1.940039443 | | 0.017118678 | 0.043955926 | 0.000375223 |
| **SIPA1L2** | 590.888184 | | 400.374969 | 823.973572 | 138.347748 | 152.821869 | 140.670227 | | 2.443947486 | | 1.320797408 | 2.961337115 | | 0.000288174 | 0.016427526 | 5.85194E-05 |
| **SLA** | 1047.19031 | | 992.89209 | 3537.75269 | 254.260742 | 211.848312 | 338.701111 | | 2.444413781 | | 2.229716276 | 3.548757218 | | 0.000155596 | 0.000304135 | 8.20178E-06 |
| **SLAMF8** | 1334.79883 | | 913.38501 | 4816.01514 | 255.507111 | 270.874756 | 198.030884 | | 2.782947497 | | 1.775361485 | 4.783437633 | | 5.4683E-05 | 0.001681731 | 0.001165472 |
| **SLC14A1** | 377.947205 | | 103.169907 | 143.459686 | 1552.98474 | 4722.92383 | 932.793762 | | -1.648638502 | | -5.537321676 | -2.313395432 | | 0.002164491 | 1.30842E-06 | 0.000365566 |
| **SLC15A3** | 729.16156 | | 857.54071 | 1087.90259 | 357.709961 | 442.294006 | 334.603912 | | 1.444215734 | | 0.994760311 | 2.014451097 | | 0.006528746 | 0.038404308 | 0.000587643 |
| **SLC15A4** | 2934.16089 | | 3491.68628 | 7624.51416 | 727.883667 | 1168.40002 | 1276.95776 | | 2.385910821 | | 1.66753238 | 2.4830579 | | 0.000102709 | 0.001343048 | 6.22385E-05 |
| **SLC16A1** | 1083.14136 | | 975.854858 | 1027.20813 | 314.086792 | 135.841675 | 303.192139 | | 2.184697474 | | 2.809632868 | 2.09487589 | | 0.000327744 | 6.72186E-05 | 0.000466201 |
| **SLC16A11** | 145.647949 | | 176.051407 | 158.173492 | 740.347412 | 402.673523 | 495.760101 | | -1.950090634 | | -1.248923348 | -1.167393605 | | 0.001373677 | 0.019888248 | 0.032706171 |
| **SLC16A3** | 688.601379 | | 1172.72949 | 927.889893 | 132.115875 | 118.861458 | 217.151123 | | 2.725150612 | | 3.254139618 | 2.471557133 | | 0.000308832 | 2.36716E-05 | 0.000163331 |
| **SLC1A4** | 1561.56714 | | 1269.2738 | 1251.59375 | 413.796875 | 298.366516 | 385.135925 | | 2.307322503 | | 2.111218211 | 1.975626601 | | 0.000174472 | 0.000370408 | 0.000614554 |
| **SLC25A25** | 1729.33887 | | 1387.58801 | 1604.72534 | 7409.70605 | 7439.75684 | 7527.90527 | | -1.740844134 | | -2.303716683 | -2.349049717 | | 0.000912538 | 0.000106066 | 9.78021E-05 |
| **SLC25A3** | 2132.17529 | | 1781.3374 | 749.484863 | 5801.88086 | 8979.2959 | 2418.70825 | | -1.105778643 | | -2.22298499 | -1.602631568 | | 0.016850863 | 0.000135703 | 0.002161932 |
| **SLC25A35** | 127.211494 | | 152.38858 | 289.678223 | 670.550354 | 344.455658 | 760.711792 | | -2.022090226 | | -1.248427945 | -1.019306562 | | 0.001168873 | 0.022416484 | 0.042831477 |
| **SLC25A5** | 2190.25 | | 1893.97241 | 1521.04053 | 8958.95215 | 10877.8447 | 3944.229 | | -1.659063719 | | -2.413753318 | -1.438825417 | | 0.001262581 | 7.18695E-05 | 0.003812618 |
| **SLC2A3** | 805.672791 | | 449.593658 | 549.928772 | 102.202843 | 135.033081 | 217.151123 | | 3.314663982 | | 1.662091869 | 1.798242042 | | 3.17576E-05 | 0.004341099 | 0.002226035 |
| **SLC2A5** | 3207.94214 | | 5237.05615 | 1847.5033 | 382.637482 | 76.0066452 | 372.84436 | | 3.458164386 | | 6.091357959 | 2.536070651 | | 8.9675E-06 | 1.11168E-06 | 8.30475E-05 |
| **SLC31A2** | 695.054138 | | 946.512939 | 680.513916 | 225.594086 | 257.128876 | 229.442688 | | 2.043953446 | | 1.897422802 | 1.986458714 | | 0.000759755 | 0.000951556 | 0.000952822 |
| **SLC33A1** | 2169.0481 | | 3090.36475 | 5009.13379 | 1418.3761 | 1636.5686 | 1567.8584 | | 0.957645959 | | 0.986789627 | 1.577258259 | | 0.03665404 | 0.030363686 | 0.001912839 |
| **SLC36A1** | 855.451233 | | 1075.23865 | 1141.24023 | 289.159271 | 203.762497 | 370.112915 | | 1.975755501 | | 2.397182302 | 1.917238482 | | 0.000789446 | 0.000173516 | 0.000803765 |
| **SLC37A4** | 2457.57861 | | 2141.95874 | 1614.84106 | 977.158936 | 462.508545 | 540.829224 | | 1.693388913 | | 2.242133796 | 1.76743545 | | 0.001326795 | 0.000181671 | 0.001176817 |
| **SLC38A1** | 2069.49146 | | 3300.49072 | 1910.95654 | 1018.28931 | 1668.10327 | 1021.56628 | | 1.384107763 | | 1.058980492 | 0.976422324 | | 0.005322135 | 0.02155751 | 0.035103146 |
| **SLC5A3** | 209.253693 | | 307.616699 | 150.816589 | 2019.12939 | 1970.5127 | 861.775818 | | -2.874129361 | | -2.662450003 | -2.11858073 | | 4.02702E-05 | 5.33532E-05 | 0.000708025 |
| **SLC6A8** | 2858.57129 | | 1651.66504 | 3662.82007 | 931.042969 | 779.472412 | 695.156738 | | 1.983318794 | | 1.1536069 | 2.445784552 | | 0.000755628 | 0.018004319 | 0.000163674 |
| **SLC7A8** | 86.6513062 | | 86.1326752 | 141.620453 | 960.955994 | 208.613983 | 893.187622 | | -3.131627647 | | -1.406369761 | -2.263603388 | | 4.98515E-05 | 0.021089127 | 0.000442723 |
| **SLC8A1** | 721.786987 | | 696.633545 | 1465.86365 | 310.347656 | 306.452332 | 572.240967 | | 1.636239099 | | 1.214150375 | 1.550391023 | | 0.003102071 | 0.016848346 | 0.002983473 |
| **SLPI** | 442.474762 | | 128.725754 | 1303.09216 | 2049.04248 | 3323.26929 | 8348.70898 | | -1.825584383 | | -4.724104277 | -2.774212228 | | 0.001483115 | 2.21216E-06 | 2.93044E-05 |
| **SLU7** | 738.379761 | | 1161.37134 | 884.66803 | 462.405548 | 636.353516 | 544.926392 | | 1.087037524 | | 0.937153115 | 0.964950763 | | 0.028095383 | 0.044854589 | 0.044630282 |
| **SMIM4** | 656.337585 | | 590.624084 | 386.23761 | 2386.8103 | 1864.58862 | 1147.21338 | | -1.493030636 | | -1.603122317 | -1.28319389 | | 0.00343073 | 0.002080473 | 0.01178399 |
| **SMIM5** | 469.207611 | | 381.444702 | 685.111938 | 10412.2266 | 16331.7266 | 9486.3623 | | -4.06761126 | | -5.378170957 | -3.802661949 | | 3.21161E-06 | 1.15664E-06 | 4.49636E-06 |
| **SMTNL2** | 226.768311 | | 361.567932 | 124.147804 | 1645.21655 | 787.558228 | 956.01123 | | -2.462161129 | | -1.088090103 | -2.557924566 | | 0.000130333 | 0.026372384 | 0.000186636 |
| **SNCB** | 365.963501 | | 774.247559 | 531.536499 | 3421.30249 | 2205.00146 | 4862 | | -2.836874229 | | -1.439867798 | -3.132095524 | | 3.47231E-05 | 0.003989493 | 0.0001178 |
| **SNX10** | 1061.93945 | | 2223.35889 | 1219.40735 | 343.999817 | 535.280884 | 359.187073 | | 2.025067983 | | 2.09881376 | 2.052144211 | | 0.000563977 | 0.000289106 | 0.000495428 |
| **SOCS3** | 3480.80151 | | 2058.66553 | 3341.875 | 135.854996 | 465.742859 | 1332.95276 | | 4.994351731 | | 2.175282066 | 1.298912801 | | 1.93542E-06 | 0.000230228 | 0.007227809 |
| **SOD2** | 5694.09717 | | 5324.13525 | 9336.83398 | 2004.17285 | 2994.17676 | 1551.4696 | | 1.86448551 | | 0.940533608 | 2.459205746 | | 0.000548797 | 0.035898782 | 6.49514E-05 |
| **SOD3** | 2380.14551 | | 1185.98071 | 1663.58057 | 7779.87988 | 4712.41211 | 13041.3584 | | -1.34690471 | | -1.883794104 | -3.107144459 | | 0.00525621 | 0.000516619 | 1.29928E-05 |
| **SOGA2** | 2229.88843 | | 4217.66162 | 4856.47803 | 807.651733 | 435.825348 | 535.366272 | | 1.83626879 | | 3.337865709 | 3.238154888 | | 0.000768724 | 9.97148E-06 | 1.15178E-05 |
| **SORT1** | 425.881958 | | 489.347198 | 383.47876 | 1823.44836 | 2293.13672 | 1307.00391 | | -1.711058245 | | -2.189174506 | -1.497787345 | | 0.001550607 | 0.000202729 | 0.004518106 |
| **SP110** | 995.568237 | | 1494.54395 | 1558.74463 | 449.941803 | 701.040039 | 611.847168 | | 1.543559153 | | 1.163852334 | 1.523064424 | | 0.003613832 | 0.015457139 | 0.003303152 |
| **SPAG4** | 1476.75952 | | 2110.72388 | 1774.85376 | 119.652115 | 175.462158 | 146.133148 | | 3.943580996 | | 3.562964673 | 3.912311686 | | 7.37796E-06 | 9.4133E-06 | 6.68317E-06 |
| **SPTBN2** | 265.484863 | | 633.217163 | 151.736206 | 10682.6904 | 8369.62598 | 16168.8809 | | -4.918074881 | | -3.606516479 | -6.573766131 | | 1.61825E-06 | 5.51613E-06 | 3.14145E-06 |
| **SQRDL** | 3926.96362 | | 3697.07959 | 2874.71143 | 1255.10083 | 2082.09692 | 1016.10333 | | 1.993949539 | | 0.911033435 | 1.525506187 | | 0.00035843 | 0.042254303 | 0.002674311 |
| **SRGAP3** | 361.354401 | | 495.026276 | 502.108887 | 2990.0564 | 1115.03369 | 2267.1123 | | -2.660436516 | | -1.120769697 | -2.014862548 | | 5.32779E-05 | 0.030904427 | 0.000451405 |
| **STK17B** | 734.692505 | | 967.336243 | 821.214722 | 321.565063 | 413.993652 | 422.010651 | | 1.60939721 | | 1.261297108 | 1.27550259 | | 0.003392734 | 0.011782832 | 0.012475258 |
| **STK19** | 105.087753 | | 117.367607 | 228.983734 | 1475.70935 | 882.162231 | 1449.03979 | | -3.492108345 | | -2.953594432 | -2.350852922 | | 1.70314E-05 | 4.82042E-05 | 0.000209192 |
| **STK39** | 644.353882 | | 785.605713 | 614.301697 | 256.753479 | 355.775787 | 297.729187 | | 1.752414004 | | 1.176187284 | 1.456956985 | | 0.002272717 | 0.030514134 | 0.007138261 |
| **SUCLG1** | 15626.7334 | | 12964.3877 | 16012.3076 | 70404.0469 | 38576.6094 | 29772.9199 | | -1.779340938 | | -1.579858921 | -1.231353806 | | 0.00074083 | 0.001740419 | 0.008462689 |
| **SULF2** | 481.191315 | | 348.316772 | 791.787109 | 300.376648 | 98.6469269 | 292.266296 | | 1.114950705 | | 1.722509908 | 1.809853097 | | 0.031347256 | 0.004782781 | 0.001543998 |
| **SUMF1** | 1740.40076 | | 1890.18628 | 1483.3363 | 4194.05566 | 6283.48584 | 2794.28418 | | -0.928896911 | | -1.623825144 | -0.933226529 | | 0.039666791 | 0.001471273 | 0.040877961 |
| **SUN1** | 5152.06543 | | 5769.94287 | 28112.5801 | 1429.59351 | 2310.92554 | 3609.62524 | | 2.202076336 | | 1.428090915 | 2.705752323 | | 0.000160095 | 0.003598676 | 2.99123E-05 |
| **SVOPL** | 77.4330826 | | 134.404831 | 106.675148 | 720.405396 | 834.455933 | 499.8573 | | -2.866320146 | | -2.6665043 | -1.743391235 | | 0.000123652 | 9.69463E-05 | 0.004559679 |
| **SYNGR1** | 124.44603 | | 143.869965 | 103.916313 | 511.014221 | 403.482086 | 367.381439 | | -1.65748277 | | -1.559433395 | -1.291361189 | | 0.005275196 | 0.006261805 | 0.02825384 |
| **TACSTD2** | 675.695862 | | 497.865814 | 695.227722 | 16839.7891 | 22342.7207 | 14628.3369 | | -4.26177314 | | -5.459800597 | -4.404285716 | | 2.47498E-06 | 1.09454E-06 | 2.24237E-06 |
| **TAP1** | 10918.0645 | | 7974.37158 | 13910.0713 | 3275.47656 | 2688.53296 | 2347.69043 | | 2.072495685 | | 1.682571648 | 2.376573625 | | 0.000228797 | 0.001159458 | 8.02817E-05 |
| **TAPBP** | 25828.543 | | 29465.8945 | 57160.4062 | 15693.1221 | 9502.44824 | 18005.7891 | | 1.095373025 | | 1.692605615 | 1.369710916 | | 0.0162795 | 0.000997064 | 0.004312679 |
| **TBC1D24** | 377.02536 | | 318.974854 | 299.793945 | 1820.95557 | 1880.76025 | 1386.21619 | | -1.882763503 | | -2.542032107 | -1.915605359 | | 0.000800639 | 7.51653E-05 | 0.000879653 |
| **TBC1D9B** | 3791.45557 | | 4430.62695 | 4446.33057 | 1779.8252 | 1845.18262 | 1955.72583 | | 1.431484182 | | 1.359660711 | 1.07076955 | | 0.003773158 | 0.005092441 | 0.02020476 |
| **TBXAS1** | 3171.06909 | | 3818.23315 | 8099.03467 | 1271.30371 | 1331.73352 | 1136.2876 | | 1.667780674 | | 1.611617902 | 2.75062718 | | 0.001381438 | 0.005638679 | 2.97674E-05 |
| **TCAIM** | 550.328003 | | 648.361389 | 432.218292 | 1633.99915 | 1526.60156 | 1304.27246 | | -1.191367927 | | -1.167571016 | -1.339384283 | | 0.014703979 | 0.014990781 | 0.008700791 |
| **TCF4** | 1308.06604 | | 2196.85645 | 1052.03772 | 255.507111 | 864.373474 | 484.834259 | | 2.753886496 | | 1.415511926 | 1.3809525 | | 5.8713E-05 | 0.004503902 | 0.00708997 |
| **TEK** | 1046.26843 | | 637.003235 | 542.571899 | 2899.07104 | 4581.42188 | 1641.60779 | | -1.12297158 | | -2.743785802 | -1.408726386 | | 0.017169507 | 3.26737E-05 | 0.005792314 |
| **TEP1** | 862.825806 | | 874.577942 | 1893.48389 | 542.173645 | 446.336914 | 695.156738 | | 1.071827279 | | 1.010188953 | 1.571899229 | | 0.028101699 | 0.035749029 | 0.00246973 |
| **TESPA1** | 577.982666 | | 420.25174 | 902.140686 | 332.78244 | 122.095787 | 364.649994 | | 1.22335273 | | 1.697750903 | 1.631106382 | | 0.018336611 | 0.004142799 | 0.002770358 |
| **TFCP2L1** | 336.465179 | | 198.767715 | 800.063599 | 8676.02441 | 4938.00635 | 9181.80469 | | -4.269254089 | | -4.60489815 | -3.556090806 | | 2.72681E-06 | 2.13768E-06 | 6.34058E-06 |
| **TFF1** | 136.429718 | | 286.793427 | 257.49173 | 968.434265 | 638.779297 | 782.563477 | | -2.454447748 | | -1.137000345 | -1.223786159 | | 0.000217753 | 0.023572549 | 0.01889468 |
| **TGFBR3** | 2093.45874 | | 2489.3291 | 1324.24329 | 5713.38818 | 4912.94043 | 3516.75537 | | -1.110756422 | | -0.880195266 | -1.440265981 | | 0.016485948 | 0.047675271 | 0.003888239 |
| **TGFBR3** | 326.325134 | | 460.951813 | 171.04808 | 1540.52087 | 1014.76959 | 950.548279 | | -1.846301173 | | -1.093798906 | -2.094435728 | | 0.001012198 | 0.023461148 | 0.000683459 |
| **THG1L** | 489.487701 | | 642.682312 | 771.555603 | 142.086884 | 235.29718 | 185.739319 | | 2.141335951 | | 1.454791234 | 2.468047343 | | 0.000849682 | 0.006745451 | 0.000198187 |
| **THSD4** | 94.0258865 | | 99.3838577 | 107.594765 | 448.695404 | 432.591034 | 363.284241 | | -1.889890397 | | -2.213835696 | -1.223353524 | | 0.002963149 | 0.000688704 | 0.035583027 |
| **TIMM23B** | 304.201416 | | 338.851624 | 175.646149 | 1657.6803 | 904.802551 | 1222.32861 | | -2.052576055 | | -1.384651776 | -2.45125219 | | 0.000461531 | 0.007261834 | 0.00018579 |
| **TIMP1** | 4377.73486 | | 3480.32812 | 7722.91309 | 1533.04272 | 1490.21545 | 1259.20337 | | 1.854239877 | | 1.305371736 | 2.523024476 | | 0.000598467 | 0.006776544 | 5.53007E-05 |
| **TIPARP** | 2486.15503 | | 2064.34473 | 6731.56982 | 170.75354 | 327.475433 | 221.248306 | | 4.209722281 | | 2.67448201 | 5.073961738 | | 3.99707E-06 | 5.34383E-05 | 1.53235E-06 |
| **TLE2** | 322.637848 | | 281.114349 | 490.153931 | 1764.86865 | 895.908142 | 2567.573 | | -2.059488908 | | -1.645575505 | -2.239787269 | | 0.000433089 | 0.002421352 | 0.000198204 |
| **TLR2** | 797.376404 | | 1251.29016 | 5663.89893 | 270.463623 | 382.458984 | 334.603912 | | 1.974784894 | | 1.740394866 | 4.195529077 | | 0.00084221 | 0.001432672 | 3.01799E-06 |
| **TLR3** | 2136.78442 | | 2109.77734 | 552.687622 | 230.57959 | 754.406372 | 213.053925 | | 3.608827544 | | 1.553977835 | 1.833784254 | | 8.68677E-06 | 0.002481687 | 0.001959691 |
| **TMCC1** | 7216.02588 | | 15210.4629 | 12550.8828 | 3989.65015 | 2122.52612 | 4220.10645 | | 1.22690133 | | 2.919731873 | 1.310618701 | | 0.009101816 | 1.83762E-05 | 0.005972852 |
| **TMED1** | 4188.76123 | | 3379.05127 | 7624.51416 | 1722.49182 | 1383.48267 | 2019.91504 | | 1.622043517 | | 1.369496479 | 1.759515755 | | 0.00158866 | 0.005065839 | 0.000823918 |
| **TMEM140** | 1291.47327 | | 1355.40649 | 448.771332 | 294.144775 | 401.864929 | 217.151123 | | 2.531106929 | | 1.783776782 | 1.535605596 | | 0.000117886 | 0.00390773 | 0.007293262 |
| **TMEM158** | 351.214355 | | 446.75412 | 748.565247 | 1792.28894 | 1479.70386 | 2456.94873 | | -1.960857013 | | -1.693431284 | -1.628614424 | | 0.000607163 | 0.001538568 | 0.001928398 |
| **TMEM159** | 1348.62622 | | 1961.1748 | 1403.32996 | 573.333008 | 731.766113 | 759.346069 | | 1.620649088 | | 1.492322876 | 1.038979778 | | 0.002252056 | 0.003314112 | 0.028433925 |
| **TMEM176A** | 4887.50244 | | 7212.42871 | 4974.18848 | 1686.34692 | 1077.83899 | 1304.27246 | | 1.882069502 | | 2.867611319 | 1.858850759 | | 0.000524891 | 2.26127E-05 | 0.000584181 |
| **TMEM178A** | 146.569763 | | 129.672272 | 221.626816 | 466.144684 | 694.571411 | 1098.04712 | | -1.258792278 | | -2.458006842 | -1.958130125 | | 0.023309777 | 0.000199229 | 0.000922518 |
| **TMEM2** | 1889.73596 | | 2546.11987 | 2768.03613 | 464.898315 | 852.244751 | 662.379211 | | 2.411636473 | | 1.649651281 | 2.151905316 | | 0.000118194 | 0.0015633 | 0.000227893 |
| **TMEM213** | 1692.46594 | | 1826.77002 | 3448.55005 | 17671.1211 | 25471.9297 | 15021.667 | | -3.028103309 | | -3.770329756 | -2.337611714 | | 1.46744E-05 | 4.00166E-06 | 9.19923E-05 |
| **TMEM248** | 1968.09094 | | 2814.92944 | 2838.84644 | 916.086487 | 1559.75342 | 1102.14429 | | 1.469410937 | | 0.915160399 | 1.381383945 | | 0.003760376 | 0.042595167 | 0.005111209 |
| **TMEM52B** | 279.312195 | | 551.817017 | 533.375732 | 18415.207 | 4052.60962 | 10385.0127 | | -5.630950252 | | -2.79637384 | -4.256560138 | | 1.0764E-06 | 2.95489E-05 | 2.7017E-06 |
| **TMEM91** | 1816.91199 | | 1950.76318 | 1159.63245 | 483.593964 | 221.551285 | 320.946625 | | 2.297416247 | | 3.13150627 | 2.164802001 | | 0.000165513 | 2.01665E-05 | 0.000957842 |
| **TNFAIP2** | 2153.3772 | | 2631.30591 | 3170.8269 | 745.332947 | 552.261047 | 1642.97351 | | 1.904968871 | | 2.300611411 | 0.898756499 | | 0.000597092 | 0.000138628 | 0.046328265 |
| **TNFAIP3** | 2693.56519 | | 1623.26965 | 2875.63086 | 663.072144 | 342.838501 | 804.415161 | | 2.401029686 | | 2.263657641 | 1.892196333 | | 0.000101202 | 0.000194981 | 0.000582542 |
| **TNFAIP8L3** | 75.5894394 | | 96.5443192 | 115.871284 | 332.78244 | 206.996826 | 621.407288 | | -1.769146984 | | -1.231151647 | -1.973793099 | | 0.006508543 | 0.036948424 | 0.001657422 |
| **TNFRSF4** | 618.542847 | | 499.75882 | 510.385406 | 220.608582 | 124.52153 | 161.156174 | | 1.908686959 | | 1.934780302 | 2.148208412 | | 0.001336462 | 0.00155326 | 0.000870862 |
| **TNIP1** | 1945.96716 | | 3313.7417 | 3435.67554 | 876.202454 | 742.27771 | 670.573547 | | 1.519151502 | | 2.245447762 | 2.419032058 | | 0.00299037 | 0.00015326 | 8.98361E-05 |
| **TNS1** | 582.591797 | | 1147.17371 | 836.848145 | 326.550568 | 409.950745 | 472.542664 | | 1.261940815 | | 1.519264265 | 1.120242813 | | 0.015652655 | 0.003656553 | 0.023734322 |
| **TNXB** | 614.85553 | | 246.093369 | 253.813293 | 2262.17261 | 667.07959 | 2829.79321 | | -1.507143054 | | -1.426691932 | -3.259799512 | | 0.003289927 | 0.007014857 | 1.58409E-05 |
| **TOX2** | 654.493896 | | 510.170471 | 1047.43958 | 421.275146 | 196.485275 | 587.264038 | | 1.054216455 | | 1.345608387 | 1.067265504 | | 0.03392268 | 0.012278507 | 0.027367871 |
| **TP53** | 802.907349 | | 1164.21094 | 1264.46838 | 418.782379 | 265.214691 | 572.240967 | | 1.348504766 | | 2.151580591 | 1.357002926 | | 0.009100199 | 0.000343974 | 0.007254638 |
| **TP53INP1** | 13911.2227 | | 14432.4297 | 20090.793 | 5067.76562 | 4814.29346 | 7149.59814 | | 1.811283071 | | 1.70868974 | 1.221248757 | | 0.000626574 | 0.000955975 | 0.008972215 |
| **TPM1** | 2582.02466 | | 1567.42542 | 3674.7749 | 390.115723 | 766.535095 | 1612.92749 | | 3.117693032 | | 1.103654373 | 1.123140082 | | 1.78289E-05 | 0.02000032 | 0.016161256 |
| **TPP1** | 3050.3103 | | 3590.12354 | 3233.3606 | 1586.63684 | 1220.95789 | 1442.21118 | | 1.285597241 | | 1.64528355 | 1.13064942 | | 0.007651217 | 0.001468427 | 0.015886119 |
| **TPX2** | 1120.93604 | | 1103.63403 | 6399.58936 | 194.434677 | 189.208038 | 204.859543 | | 2.898996079 | | 2.535278245 | 5.121334991 | | 4.8547E-05 | 0.000116455 | 1.51116E-06 |
| **TRABD2B** | 797.376404 | | 1510.63464 | 1104.45569 | 236.811462 | 80.0495529 | 390.598877 | | 2.167687092 | | 4.17916815 | 1.788461717 | | 0.000448593 | 5.76677E-06 | 0.001325176 |
| **TRADD** | 3506.61255 | | 4219.55469 | 4025.14771 | 1896.9845 | 1091.58484 | 1513.22913 | | 1.2269538 | | 2.055126721 | 1.344300086 | | 0.009833448 | 0.000278699 | 0.00568572 |
| **TRAF3IP3** | 683.070435 | | 582.105469 | 508.546204 | 413.796875 | 216.699799 | 252.660095 | | 1.140323516 | | 1.414851191 | 1.465982329 | | 0.02338982 | 0.00848856 | 0.007975094 |
| **TRAF7** | 2389.36377 | | 2773.28296 | 3964.45312 | 1621.5354 | 1406.12292 | 1817.78699 | | 0.902419838 | | 1.04469561 | 1.034471858 | | 0.046402082 | 0.023571141 | 0.024236413 |
| **TREM2** | 2479.70239 | | 2366.28223 | 5598.60596 | 105.941971 | 94.6040192 | 146.133148 | | 4.865399931 | | 4.583855629 | 5.436038712 | | 2.43473E-06 | 3.04209E-06 | 3.06532E-06 |
| **TRIB3** | 588.122681 | | 699.473083 | 482.797028 | 105.941971 | 101.881248 | 103.795502 | | 2.822390557 | | 2.731073062 | 2.719195694 | | 0.000119588 | 0.000116203 | 0.00019891 |
| **TRIM2** | 2287.96338 | | 3015.59033 | 2716.53784 | 10145.502 | 11027.4326 | 12824.207 | | -1.783940135 | | -1.753016725 | -2.43773061 | | 0.000740319 | 0.000802374 | 6.85906E-05 |
| **TRIM2** | 409.289154 | | 665.398621 | 137.022385 | 1571.6803 | 4597.59375 | 1253.74036 | | -1.552417678 | | -2.681783745 | -2.842212967 | | 0.003180791 | 3.82349E-05 | 7.05152E-05 |
| **TRIM21** | 322.637848 | | 612.39386 | 757.761414 | 150.811508 | 167.376343 | 307.289307 | | 1.470866353 | | 1.844132847 | 1.676792948 | | 0.012094866 | 0.001674343 | 0.002620003 |
| **TRNP1** | 148.413406 | | 115.474579 | 203.234558 | 654.347473 | 481.105896 | 609.115723 | | -1.736635593 | | -2.138845079 | -1.144044773 | | 0.003079977 | 0.000749407 | 0.034089744 |
| **TRPV2** | 1715.5116 | | 1302.40186 | 2824.13257 | 795.187988 | 291.897858 | 981.960083 | | 1.481438293 | | 2.178681173 | 1.555500969 | | 0.003672906 | 0.000291818 | 0.002355763 |
| **TSPAN33** | 1895.26697 | | 1360.13904 | 2196.9563 | 10657.7627 | 4136.70215 | 4536.95605 | | -2.130900632 | | -1.510312775 | -1.176597745 | | 0.000191295 | 0.002570935 | 0.012414442 |
| **TTC18** | 610.24646 | | 470.416931 | 473.600891 | 1688.83972 | 982.426331 | 1272.8606 | | -1.094201839 | | -1.015695593 | -1.182337336 | | 0.022306994 | 0.033261109 | 0.017234802 |
| **TTC28** | 1300.69141 | | 1178.40857 | 1475.05981 | 749.072083 | 472.211517 | 580.435364 | | 1.172366731 | | 1.36066884 | 1.535710931 | | 0.019635988 | 0.007116884 | 0.00316602 |
| **TUBA1B** | 7587.52051 | | 5754.79883 | 14116.0654 | 2341.94092 | 2633.54956 | 2043.13257 | | 2.064435206 | | 1.236377621 | 2.615095168 | | 0.000260126 | 0.008824968 | 3.98133E-05 |
| **TUBA4B** | 368.728973 | | 437.288971 | 504.867737 | 2968.86792 | 891.056641 | 3025.09253 | | -2.621372635 | | -0.986430948 | -2.456648745 | | 5.91531E-05 | 0.038864829 | 9.46502E-05 |
| **TUBB2B** | 94.0258865 | | 168.479309 | 177.485382 | 1555.47742 | 360.627289 | 917.770752 | | -3.728849836 | | -1.160222456 | -1.987220628 | | 1.17559E-05 | 0.030027889 | 0.000998988 |
| **TYMP** | 2462.18774 | | 2850.89697 | 5848.74121 | 722.898193 | 515.066345 | 588.629761 | | 2.143484079 | | 2.513672823 | 3.339238264 | | 0.000239501 | 7.12334E-05 | 9.20566E-06 |
| **TYROBP** | 12074.9521 | | 9350.60156 | 22998.6113 | 4444.57715 | 3286.07471 | 2939.05151 | | 1.781556581 | | 1.632464977 | 2.767054443 | | 0.000711531 | 0.001370047 | 2.58397E-05 |
| **UBD** | 7721.18457 | | 10061.4326 | 8253.53027 | 1056.927 | 487.574554 | 734.762878 | | 3.253934685 | | 4.454545458 | 3.462226207 | | 1.00771E-05 | 2.15547E-06 | 7.03623E-06 |
| **UCHL1** | 729.16156 | | 432.556427 | 288.758606 | 3923.59204 | 3467.19678 | 3042.84717 | | -2.06368161 | | -2.957272324 | -3.197931701 | | 0.000297828 | 2.13097E-05 | 1.69624E-05 |
| **UMOD** | 98.6350021 | | 156.174637 | 127.826256 | 81625.1719 | 52970.1641 | 18804.7402 | | -6.64385619 | | -6.64385619 | -6.64385619 | | 6.11372E-07 | 6.29108E-07 | 7.85468E-07 |
| **UNC5CL** | 1552.349 | | 1765.24658 | 2823.21313 | 634.405457 | 346.881409 | 1432.65112 | | 1.672734891 | | 2.366511345 | 0.960871419 | | 0.001707095 | 0.000135653 | 0.035432819 |
| **UQCRHL** | 940.25885 | | 2084.22144 | 1536.67395 | 3327.82422 | 4063.12134 | 3184.88306 | | -1.471024062 | | -0.875268471 | -1.091041723 | | 0.003427224 | 0.04936431 | 0.019524582 |
| **USH1C** | 2051.97681 | | 2442.94995 | 3668.33765 | 719.159058 | 119.670044 | 566.778076 | | 1.888422407 | | 4.295605815 | 2.775794266 | | 0.000647878 | 3.76427E-06 | 3.33732E-05 |
| **USP2** | 530.047913 | | 424.037781 | 391.75528 | 3245.56348 | 863.56488 | 1744.0376 | | -2.236544782 | | -0.986683993 | -1.925914548 | | 0.000176173 | 0.039226987 | 0.000722887 |
| **UXS1** | 1097.8905 | | 1610.01855 | 636.372437 | 6785.27197 | 5448.22119 | 4801.90771 | | -2.272586951 | | -1.653962881 | -2.882570991 | | 0.000126682 | 0.001312127 | 2.62975E-05 |
| **VASH1** | 353.057983 | | 565.068237 | 510.385406 | 210.637573 | 96.2211838 | 240.36853 | | 1.175374883 | | 2.487375935 | 1.546964395 | | 0.030961882 | 0.000273746 | 0.005878349 |
| **VAT1** | 1180.85449 | | 1741.58386 | 1236.88 | 802.66626 | 859.521973 | 572.240967 | | 0.931082261 | | 1.087334922 | 1.327937471 | | 0.046471067 | 0.021012012 | 0.008301349 |
| **VCAM1** | 25803.6543 | | 33892.7344 | 26059.084 | 3064.83887 | 4535.33301 | 6642.91211 | | 3.44861949 | | 2.91837932 | 1.675661563 | | 6.9105E-06 | 1.76372E-05 | 0.001087854 |
| **VCAN** | 2215.1394 | | 2620.89429 | 653.845093 | 137.101379 | 358.201538 | 251.294373 | | 4.330771414 | | 2.893546459 | 1.796152326 | | 3.84864E-06 | 2.83378E-05 | 0.001905109 |
| **VEGFA** | 9703.10254 | | 10295.2217 | 13310.4834 | 1066.89795 | 1600.99109 | 2825.69604 | | 3.549069247 | | 2.795784188 | 2.02138336 | | 5.97533E-06 | 3.07133E-05 | 0.00027477 |
| **VEGFA** | 2462.18774 | | 2057.71924 | 2009.35522 | 92.2318344 | 97.8383408 | 111.989883 | | 5.055118234 | | 4.333006872 | 4.463600104 | | 2.19646E-06 | 4.02707E-06 | 3.63781E-06 |
| **VIM** | 21757.7754 | | 27466.8594 | 11843.7002 | 5374.37402 | 7463.20605 | 4088.99658 | | 2.393730131 | | 1.957856684 | 1.278341841 | | 7.33754E-05 | 0.000462312 | 0.00848819 |
| **VTCN1** | 133.664246 | | 129.672272 | 178.404999 | 2010.40479 | 1273.51562 | 1242.81458 | | -3.562777748 | | -3.335716189 | -2.455494587 | | 1.17461E-05 | 1.71713E-05 | 0.000180616 |
| **VWA1** | 6653.71436 | | 4410.75049 | 10422.8975 | 4134.22949 | 2038.43359 | 3948.32617 | | 1.054899859 | | 1.209843724 | 1.153206837 | | 0.048425123 | 0.010268446 | 0.013322403 |
| **VWF** | 14086.3682 | | 15218.9814 | 22313.498 | 1895.73816 | 1087.54187 | 4691.28369 | | 3.25312723 | | 3.896220831 | 1.996895617 | | 9.36645E-06 | 3.57195E-06 | 0.000291976 |
| **WDFY4** | 292.217712 | | 326.546967 | 634.533203 | 122.144867 | 80.0495529 | 127.012917 | | 1.626234635 | | 1.928801028 | 2.777638857 | | 0.008181285 | 0.002772415 | 0.000118514 |
| **WDR36** | 190.817245 | | 367.247009 | 240.019089 | 77.275322 | 142.310318 | 158.424713 | | 1.676056577 | | 1.290556113 | 1.176532206 | | 0.013124478 | 0.019750489 | 0.042404234 |
| **WDR55** | 1163.33997 | | 1608.12549 | 1685.65125 | 668.057617 | 705.082947 | 905.479187 | | 1.181732539 | | 1.259891491 | 1.001794739 | | 0.01567122 | 0.009847612 | 0.032129459 |
| **WDR81** | 231.377426 | | 292.472504 | 206.91301 | 129.623123 | 95.4125977 | 120.184265 | | 1.206119597 | | 1.513143516 | 1.381313852 | | 0.040524751 | 0.011635833 | 0.025933048 |
| **WIPF1** | 2730.43799 | | 2933.24365 | 4724.05371 | 769.014099 | 1355.99097 | 1704.4314 | | 2.20090238 | | 1.18342536 | 1.367856622 | | 0.000189984 | 0.012266059 | 0.004982704 |
| **WIPF1** | 629.604736 | | 445.807587 | 1001.45892 | 168.260788 | 135.841675 | 200.76236 | | 2.277248523 | | 1.640984029 | 2.692146792 | | 0.000418103 | 0.004712418 | 0.000121259 |
| **WIPF1** | 761.425354 | | 904.866394 | 716.378784 | 160.782532 | 305.643738 | 198.030884 | | 2.600998194 | | 1.593788386 | 2.277395043 | | 0.000142195 | 0.003086543 | 0.000364739 |
| **XAF1** | 3613.54395 | | 3365.80005 | 5038.56152 | 1747.41931 | 1411.78308 | 1877.87915 | | 1.389086732 | | 1.333780025 | 1.299895488 | | 0.00461542 | 0.005974038 | 0.006857235 |
| **YEATS2** | 712.568726 | | 1132.97595 | 739.369141 | 127.130371 | 410.759338 | 236.271332 | | 2.828595553 | | 1.498833294 | 2.047662429 | | 9.14774E-05 | 0.004005219 | 0.000719954 |
| **ZNF395** | 23382.9473 | | 27417.6406 | 14238.374 | 4825.96826 | 3169.63892 | 3657.42578 | | 2.651871729 | | 3.144454239 | 1.720615027 | | 3.46369E-05 | 8.74505E-05 | 0.00094955 |
| **ZNF395** | 1581.84729 | | 3083.73926 | 597.748657 | 213.130325 | 186.782288 | 152.961792 | | 3.277198772 | | 4.035654877 | 2.427540815 | | 1.76646E-05 | 4.32972E-06 | 0.000296351 |
| **ZNF503** | 85.7294846 | | 131.565292 | 169.208862 | 1070.63708 | 536.89801 | 1057.0752 | | -3.308239203 | | -2.088194496 | -2.27624742 | | 3.22549E-05 | 0.000772214 | 0.000352608 |
| **ZNF541** | 375.181732 | | 255.558487 | 524.179626 | 180.724548 | 66.3036728 | 117.452805 | | 1.456349019 | | 1.835745666 | 2.647434496 | | 0.010816464 | 0.00523133 | 0.000206242 |
| **ZNF608** | 324.481506 | | 548.977478 | 905.819153 | 82.2608261 | 135.841675 | 152.961792 | | 2.345834157 | | 1.960511695 | 2.962510676 | | 0.000915747 | 0.001278498 | 5.26578E-05 |
| **ZNF654** | 323.559662 | | 377.658661 | 362.327667 | 112.173851 | 145.544647 | 128.378647 | | 1.89431971 | | 1.301153694 | 2.038897654 | | 0.003122801 | 0.018554865 | 0.001713776 |
| **ZNF710** | 1278.56775 | | 1439.64612 | 1542.19165 | 523.477966 | 439.868256 | 725.202759 | | 1.676835323 | | 1.741637925 | 1.23605567 | | 0.001841442 | 0.001327279 | 0.011527368 |
| **ZNF768** | 968.835388 | | 1058.20142 | 1033.64539 | 443.7099 | 439.059662 | 620.041565 | | 1.525976347 | | 1.307337703 | 0.962540379 | | 0.0039427 | 0.009302468 | 0.043012321 |
