# Supplementary material for: SERPINH1 overexpression in clear cell renal cell carcinoma: association with poor clinical outcome and its potential as a novel prognostic marker
Source: J Cell Mol Med. 2017 Dec 14;22(2):1224–35. doi: 10.1111/jcmm.13495 (PMC5783852; doi:10.1111/jcmm.13495)
Supplement: Supplementary file 12 — Table S2. Consistently dysregulated 35 genes and their correlation with TGFβ level. [file JCMM-22-1224-s012.docx]

Supplementary Table 2 Consistently dysregulated 35 genes and their correlation with TGFβ level

| **No.** | **Accession number** | **Gene symbol** | **Regulation** | **TGF core enrichment** |
| --- | --- | --- | --- | --- |
| 1 | P53396 | ACLY | Up | No |
| 2 | P09525 | ANXA4 | Up | No |
| 3 | P31146 | CORO1A | Up | Yes |
| 4 | Q9NZN4 | EHD2 | Up | Yes |
| 5 | Q969X5 | ERGIC1 | Up | Yes |
| 6 | P37235 | HPCAL1 | Up | Yes |
| 7 | P40261 | NNMT | Up | Yes |
| 8 | P07237 | P4HB | Up | Yes |
| 9 | Q01813 | PFKP | Up | Yes |
| 10 | Q99541 | PLIN2 | Up | No |
| 11 | P28065 | PSMB9 | Up | No |
| 12 | P08575 | PTPRC | Up | No |
| 13 | P50454 | SERPINH1 | Up | Yes |
| 14 | P04179 | SOD2 | Up | No |
| 15 | O15533 | TAPBP | Up | Yes |
| 16 | P19971 | TYMP | Up | Yes |
| 17 | P08670 | VIM | Up | Yes |
| 18 | P24752 | ACAT1 | Down | Yes |
| 19 | P05023 | ATP1A1 | Down | Yes |
| 20 | P00918 | CA2 | Down | Yes |
| 21 | O75309 | CDH16 | Down | Yes |
| 22 | P12277 | CKB | Down | No |
| 23 | P36269 | GGT5 | Down | No |
| 24 | P23378 | GLDC | Down | Yes |
| 25 | Q9UBQ7 | GRHPR | Down | Yes |
| 26 | Q16836 | HADH | Down | Yes |
| 27 | P07195 | LDHB | Down | Yes |
| 28 | P35580 | MYH10 | Down | No |
| 29 | P49821 | NDUFV1 | Down | Yes |
| 30 | Q96F10 | SAT2 | Down | Yes |
| 31 | Q13228 | SELENBP1 | Down | Yes |
| 32 | P05141 | SLC25A5 | Down | Yes |
| 33 | O15020 | SPTBN2 | Down | Yes |
| 34 | P53597 | SUCLG1 | Down | Yes |
| 35 | P07911 | UMOD | Down | Yes |
