# Supplementary material for: SERPINH1 overexpression in clear cell renal cell carcinoma: association with poor clinical outcome and its potential as a novel prognostic marker
Source: J Cell Mol Med. 2017 Dec 14;22(2):1224–35. doi: 10.1111/jcmm.13495 (PMC5783852; doi:10.1111/jcmm.13495)
Supplement: Supplementary file 13 — Table S3. The genes significantly correlated with poor prognosis by univariate cox regression analysis. [file JCMM-22-1224-s013.docx]

Supplementary Table 3 The genes significantly correlated with poor prognosis by univariate cox regression analysis

| **Low *vs.* High** | **Regulation** | ***P* values** | **HR** | **95% CI** |
| --- | --- | --- | --- | --- |
| **Overall Survival (OS)** | | | | |
| ACAT1 | Down | 0.000 | 2.129 | 1.545-2.935 |
| CA2 | Down | 0.003 | 1.617 | 1.183-2.208 |
| CDH16 | Down | 0.001 | 1.715 | 1.253-2.349 |
| GLDC | Down | 0.007 | 1.531 | 1.122-2.089 |
| GRHPR | Down | 0.003 | 1.607 | 1.177-2.194 |
| HADH | Down | 0.000 | 1.876 | 1.364-2.579 |
| MYH10 | Down | 0.002 | 1.636 | 1.195-2.240 |
| CORO1A | Up | 0.001 | 0.583 | 0.425-0.801 |
| NNMT | Up | 0.000 | 0.535 | 0.389-0.735 |
| P4HB | Up | 0.001 | 0.592 | 0.432-0.812 |
| SERPINH1 | Up | 0.000 | 0.494 | 0.360-0.679 |
| SOD2 | Up | 0.001 | 0.599 | 0.438-0.819 |
| TAPBP | Up | 0.002 | 0.615 | 0.449-0.842 |
| TYMP | Up | 0.002 | 0.602 | 0.439-0.825 |
| VIM | Up | 0.008 | 0.657 | 0.482-0.894 |
| **Disease-free Survival (DFS)** | | | | |
| ACAT1 | Down | 0.000 | 2.940 | 1.759-4.913 |
| GRHPR | Down | 0.000 | 2.449 | 1.496-4.010 |
| HADH | Down | 0.000 | 2.431 | 1.486-3.978 |
| NNMT | Up | 0.003 | 0.480 | 0.296-0.777 |
| P4HB | Up | 0.003 | 0.478 | 0.294-0.778 |
| SERPINH1 | Up | 0.000 | 0.324 | 0.193-0.541 |
| SOD2 | Up | 0.004 | 0.496 | 0.307-0.800 |
| TYMP | Up | 0.005 | 0.503 | 0.312-0.811 |
| VIM | Up | 0.000 | 0.383 | 0.233-0.632 |

The expression level data and clinical prognosis information were obtained from TCGA_KIRC dataset.
