# Supplementary material for: SERPINH1 overexpression in clear cell renal cell carcinoma: association with poor clinical outcome and its potential as a novel prognostic marker
Source: J Cell Mol Med. 2017 Dec 14;22(2):1224–35. doi: 10.1111/jcmm.13495 (PMC5783852; doi:10.1111/jcmm.13495)
Supplement: Supplementary file 14 — Table S4. The genes significantly differentially expressed between patients with good and poor prognosis. [file JCMM-22-1224-s014.docx]

Supplementary Table 4 The genes significantly differentially expressed between patients with good and poor prognosis

|  | **Regulation** | **Fold change (good/poor)** | ***P* values** |
| --- | --- | --- | --- |
| **OS** | | | |
| ACAT1 | Down | 1.524423601 | 0.0071548 |
| SELENBP1 | Down | 0.795406839 | 0.0256678 |
| PLIN2 | Up | 1.268412562 | 0.0058467 |
| TYMP | Up | 0.727566369 | 0.0026102 |
| SERPINH1 | Up | 0.666925705 | 0.000286 |
| **DFS** | | | |
| HADH | Down | 1.253667957 | 0.0095891 |
| PLIN2 | Up | 1.589795467 | 0.0005103 |
| ACAT1 | Up | 1.518444133 | 0.0058982 |
| SOD2 | Up | 0.785313295 | 0.0488484 |
| P4HB | Up | 0.727215744 | 7.611E-05 |
| TYMP | Up | 0.701795834 | 0.012977 |
| SERPINH1 | Up | 0.647675834 | 0.0001256 |

The expression level data and clinical prognosis information were obtained from TCGA_KIRC dataset.
