# Supplementary material for: SERPINH1 overexpression in clear cell renal cell carcinoma: association with poor clinical outcome and its potential as a novel prognostic marker
Source: J Cell Mol Med. 2017 Dec 14;22(2):1224–35. doi: 10.1111/jcmm.13495 (PMC5783852; doi:10.1111/jcmm.13495)
Supplement: Supplementary file 15 — Table S5. GSEA rank score of 35 genes for OS and DFS prognosis in ccRCC samples. [file JCMM-22-1224-s015.docx]

Supplementary Table 5 GSEA rank score of 35 genes for OS and DFS prognosis in ccRCC samples

|  | **Total-SCORE** | **Stage I/II-SCORE** | **Stage III/IV-SCORE** |
| --- | --- | --- | --- |
| **OS** | | | |
| SERPINH1 | -0.28184685 | -0.17092873 | -0.26248434 |
| TYMP | -0.22887446 | -0.1798566 | -0.079688005 |
| P4HB | -0.21213163 | -0.13918939 | -0.15482542 |
| SELENBP1 | -0.1705144 | -0.15815775 | -0.25145185 |
| TAPBP | -0.15120459 | 0.032944486 | -0.07100075 |
| PSMB9 | -0.13330406 | 0.069945484 | -0.042616405 |
| NNMT | -0.13202512 | -0.2506532 | 0.02217386 |
| VIM | -0.11651922 | -0.03335286 | -0.21175343 |
| GGT5 | -0.114059746 | -0.059962925 | -0.032885294 |
| SOD2 | -0.10466408 | -0.15638475 | 0.057041224 |
| CORO1A | -0.09241205 | 0.07143769 | 0.043168172 |
| LDHB | -0.084059246 | -0.12833044 | -0.22511151 |
| SAT2 | -0.07634445 | 0.13385838 | -0.2062721 |
| CKB | -0.073789515 | -0.12777491 | -0.15395127 |
| SPTBN2 | -0.06565045 | 0.050975937 | -0.10390181 |
| NDUFV1 | -0.031637255 | 0.04902292 | -0.14006841 |
| HPCAL1 | -0.023525096 | -0.010373422 | -0.06167546 |
| ERGIC1 | -0.01679945 | 0.048548207 | -0.061467707 |
| SUCLG1 | 0.009193255 | -0.028153606 | -0.038140874 |
| PTPRC | 0.015897889 | 0.07211921 | 0.068125315 |
| UMOD | 0.016901232 | -0.0759788 | -6.76228E-05 |
| EHD2 | 0.019684276 | -0.02426885 | 0.02014308 |
| CDH16 | 0.028354682 | 0.060206026 | 0.035507895 |
| SLC25A5 | 0.052593153 | 0.08049362 | -0.14974393 |
| GLDC | 0.057079844 | 0.051014412 | 0.01330831 |
| GRHPR | 0.101093605 | 0.12257346 | 0.05879218 |
| PFKP | 0.112993605 | 0.10093498 | -0.0329101 |
| MYH10 | 0.11368864 | 0.006465594 | 0.2059288 |
| ANXA4 | 0.11444018 | -0.028024273 | 0.22068875 |
| ATP1A1 | 0.13203272 | -0.037534893 | 0.13777351 |
| PLIN2 | 0.14584896 | 0.020156974 | 0.170461 |
| HADH | 0.15521425 | 0.10083367 | 0.1717032 |
| CA2 | 0.17618053 | 0.18125492 | 0.11665402 |
| ACLY | 0.2317051 | 0.1139578 | 0.106200084 |
| ACAT1 | 0.2508844 | 0.24896276 | 0.21782736 |
| **DFS** | | | |
| P4HB | -0.33107185 | -0.3376369 | -0.24926773 |
| SERPINH1 | -0.32289144 | -0.43445697 | -0.3167207 |
| TYMP | -0.24194922 | -0.3476687 | -0.074825004 |
| TAPBP | -0.22715054 | 0.0895537 | -0.12698339 |
| SOD2 | -0.17174786 | 0.003581525 | -0.06141821 |
| GGT5 | -0.16333552 | -0.05911322 | -0.11382027 |
| NNMT | -0.15351088 | -0.39154482 | 0.007309243 |
| CORO1A | -0.14843525 | -0.04739655 | -0.026633592 |
| SELENBP1 | -0.12877578 | -0.29537603 | -0.1814155 |
| PSMB9 | -0.11343119 | 0.04796616 | -0.007519866 |
| SAT2 | -0.082692385 | 0.14497001 | -0.24345279 |
| VIM | -0.07444161 | -0.05921754 | -0.19055721 |
| CKB | -0.06288556 | -0.019884985 | -0.16290008 |
| SPTBN2 | -0.024352048 | 0.20040263 | -0.049519707 |
| EHD2 | -0.012459359 | -0.21358745 | -0.040059894 |
| CDH16 | -0.011405231 | 0.2127245 | 0.017775364 |
| LDHB | -0.00441093 | 0.18947572 | -0.19224541 |
| PTPRC | 0.017205823 | 0.1208283 | 0.05650656 |
| ANXA4 | 0.024271592 | -0.21363206 | 0.101585194 |
| GLDC | 0.038777135 | 0.47215536 | -0.011003339 |
| ERGIC1 | 0.0485804 | 0.108861476 | -0.01146777 |
| SUCLG1 | 0.067753725 | 0.19042361 | 0.008120996 |
| NDUFV1 | 0.06950032 | 0.3056925 | -0.0213546 |
| HPCAL1 | 0.070472926 | -0.108094476 | 0.085059084 |
| PFKP | 0.09381304 | -0.1670939 | 0.038075443 |
| MYH10 | 0.09733673 | 0.06901731 | 0.15795308 |
| SLC25A5 | 0.10207111 | 0.1500043 | -0.063377514 |
| UMOD | 0.10566613 | 0.2201309 | 0.046433896 |
| CA2 | 0.14158273 | 0.23957914 | -0.07499743 |
| ATP1A1 | 0.18122378 | 0.2715607 | 0.12173556 |
| HADH | 0.2194536 | 0.33529416 | 0.2638524 |
| GRHPR | 0.22226365 | 0.35194555 | 0.24115635 |
| ACLY | 0.23255211 | 0.363191 | 0.1645353 |
| ACAT1 | 0.24472404 | 0.38081273 | 0.22967833 |
| PLIN2 | 0.2940191 | 0.37893716 | 0.32732993 |

The expression level data and clinical prognosis information were obtained from TCGA_KIRC dataset.
