# Supplementary material for: SERPINH1 overexpression in clear cell renal cell carcinoma: association with poor clinical outcome and its potential as a novel prognostic marker
Source: J Cell Mol Med. 2017 Dec 14;22(2):1224–35. doi: 10.1111/jcmm.13495 (PMC5783852; doi:10.1111/jcmm.13495)
Supplement: Supplementary file 16 — Table S6. VHL mutation in TCGA_KIRC dataset. [file JCMM-22-1224-s016.docx]

Supplementary Table 6 *VHL* mutation in TCGA_KIRC dataset

| **Sample ID** | ***VHL* mutation** |
| --- | --- |
| TCGA-CZ-4858 | Mut |
| TCGA-B0-4712 | Mut |
| TCGA-B0-4700 | Mut |
| TCGA-CJ-5677 | Mut |
| TCGA-CJ-6033 | Mut |
| TCGA-B0-4703 | Mut |
| TCGA-BP-5175 | Mut |
| TCGA-BP-5177 | Mut |
| TCGA-B0-4810 | Mut |
| TCGA-B0-5095 | Mut |
| TCGA-BP-5169 | Mut |
| TCGA-B0-5097 | Mut |
| TCGA-BP-5198 | Mut |
| TCGA-B0-4822 | Mut |
| TCGA-BP-4960 | Mut |
| TCGA-BP-5196 | Mut |
| TCGA-B0-5107 | Mut |
| TCGA-B0-5107 | Mut |
| TCGA-B8-5163 | Mut |
| TCGA-BP-4159 | Mut |
| TCGA-B0-5108 | Mut |
| TCGA-A3-3308 | Mut |
| TCGA-CZ-5469 | Mut |
| TCGA-CJ-5676 | Mut |
| TCGA-B0-5081 | Mut |
| TCGA-CW-6090 | Mut |
| TCGA-B0-4842 | Mut |
| TCGA-CJ-4872 | Mut |
| TCGA-CJ-4920 | Mut |
| TCGA-BP-4166 | Mut |
| TCGA-BP-4167 | Mut |
| TCGA-CJ-5679 | Mut |
| TCGA-B0-5116 | Mut |
| TCGA-BP-4762 | Mut |
| TCGA-BP-4759 | Mut |
| TCGA-AK-3445 | Mut |
| TCGA-CJ-4908 | Mut |
| TCGA-BP-4974 | Mut |
| TCGA-BP-5009 | Mut |
| TCGA-B0-5710 | Mut |
| TCGA-BP-4342 | Mut |
| TCGA-BP-5195 | Mut |
| TCGA-BP-5170 | Mut |
| TCGA-EU-5905 | Mut |
| TCGA-BP-5176 | Mut |
| TCGA-CZ-5470 | Mut |
| TCGA-BP-4798 | Mut |
| TCGA-A3-3349 | Mut |
| TCGA-BP-4758 | Mut |
| TCGA-BP-4986 | Mut |
| TCGA-B0-4827 | Mut |
| TCGA-B0-5088 | Mut |
| TCGA-BP-4968 | Mut |
| TCGA-B0-4690 | Mut |
| TCGA-B0-4706 | Mut |
| TCGA-BP-4782 | Mut |
| TCGA-CJ-4912 | Mut |
| TCGA-CZ-5460 | Mut |
| TCGA-B8-5550 | Mut |
| TCGA-B0-5106 | Mut |
| TCGA-CW-6093 | Mut |
| TCGA-BP-5174 | Mut |
| TCGA-BP-5194 | Mut |
| TCGA-BP-4991 | Mut |
| TCGA-B0-5695 | Mut |
| TCGA-B0-5695 | Mut |
| TCGA-CZ-5987 | Mut |
| TCGA-BP-4341 | Mut |
| TCGA-B0-4816 | Mut |
| TCGA-B8-4153 | Mut |
| TCGA-CW-5588 | Mut |
| TCGA-CZ-5466 | Mut |
| TCGA-BP-4789 | Mut |
| TCGA-B0-5696 | Mut |
| TCGA-CJ-6030 | Mut |
| TCGA-BP-4964 | Mut |
| TCGA-B0-4819 | Mut |
| TCGA-A3-3365 | Mut |
| TCGA-CJ-6027 | Mut |
| TCGA-BP-4988 | Mut |
| TCGA-A3-3316 | Mut |
| TCGA-AS-3778 | Mut |
| TCGA-B0-4713 | Mut |
| TCGA-BP-5184 | Mut |
| TCGA-BP-4355 | Mut |
| TCGA-B0-4845 | Mut |
| TCGA-BP-4961 | Mut |
| TCGA-BP-4961 | Mut |
| TCGA-BP-5200 | Mut |
| TCGA-BP-5007 | Mut |
| TCGA-A3-3376 | Mut |
| TCGA-CJ-4637 | Mut |
| TCGA-BP-4765 | Mut |
| TCGA-CW-5580 | Mut |
| TCGA-BP-4999 | Mut |
| TCGA-B0-5693 | Mut |
| TCGA-B0-5713 | Mut |
| TCGA-B0-5099 | Mut |
| TCGA-B0-4710 | Mut |
| TCGA-EU-5906 | Mut |
| TCGA-EU-5906 | Mut |
| TCGA-A3-3383 | Mut |
| TCGA-CJ-4634 | Mut |
| TCGA-BP-5173 | Mut |
| TCGA-B0-5115 | Mut |
| TCGA-BP-5008 | Mut |
| TCGA-CJ-4875 | Mut |
| TCGA-BP-4349 | Mut |
| TCGA-B0-5077 | Mut |
| TCGA-CJ-5684 | Mut |
| TCGA-B0-5812 | Mut |
| TCGA-CJ-4644 | Mut |
| TCGA-B0-5096 | Mut |
| TCGA-BP-5001 | Mut |
| TCGA-BP-4970 | Mut |
| TCGA-BP-4989 | Mut |
| TCGA-B0-5110 | Mut |
| TCGA-B0-5110 | Mut |
| TCGA-CJ-4885 | Mut |
| TCGA-BP-4982 | Mut |
| TCGA-BP-4998 | Mut |
| TCGA-B0-5709 | Mut |
| TCGA-BP-4771 | Mut |
| TCGA-CW-5591 | Mut |
| TCGA-BP-4763 | Mut |
| TCGA-B0-5711 | Mut |
| TCGA-B8-4621 | Mut |
| TCGA-B0-5399 | Mut |
| TCGA-B0-5703 | Mut |
| TCGA-BP-4335 | Mut |
| TCGA-AK-3451 | Mut |
| TCGA-AK-3451 | Mut |
| TCGA-B0-4707 | Mut |
| TCGA-CW-5581 | Mut |
| TCGA-CJ-4638 | Mut |
| TCGA-BP-4807 | Mut |
| TCGA-BP-5186 | Mut |
| TCGA-CJ-4894 | Mut |
| TCGA-B0-4823 | Mut |
| TCGA-B0-5102 | Mut |
| TCGA-B0-5085 | Mut |
| TCGA-BP-4967 | Mut |
| TCGA-EU-5907 | Mut |
| TCGA-B0-4945 | Mut |
| TCGA-A3-3373 | Mut |
| TCGA-BP-4975 | Mut |
| TCGA-BP-5006 | Mut |
| TCGA-B0-4714 | Mut |
| TCGA-CJ-4636 | Mut |
| TCGA-CJ-4636 | Mut |
| TCGA-BP-4963 | Mut |
| TCGA-BP-4963 | Mut |
| TCGA-B8-5551 | Mut |
| TCGA-A3-3362 | Mut |
| TCGA-BP-4329 | Mut |
| TCGA-CZ-4856 | Mut |
| TCGA-B8-5165 | Mut |
| TCGA-B0-5692 | Mut |
| TCGA-CZ-5465 | Mut |
| TCGA-CZ-5982 | Mut |
| TCGA-BP-5192 | Mut |
| TCGA-EU-5904 | Mut |
| TCGA-B0-4852 | Mut |
| TCGA-CZ-5458 | Mut |
| TCGA-CJ-5680 | Mut |
| TCGA-BP-5180 | Mut |
| TCGA-CJ-4884 | Mut |
| TCGA-BP-5187 | Mut |
| TCGA-BP-4161 | Mut |
| TCGA-CZ-5984 | Mut |
| TCGA-B8-5549 | Mut |
| TCGA-A3-3382 | Mut |
| TCGA-BP-4977 | Mut |
| TCGA-B8-4151 | Mut |
| TCGA-CJ-5672 | Mut |
| TCGA-B8-4154 | Mut |
| TCGA-CJ-5686 | Mut |
| TCGA-CJ-4892 | Mut |
| TCGA-B0-5113 | Mut |
| TCGA-B0-4847 | Mut |
| TCGA-CJ-4916 | Mut |
| TCGA-CJ-4903 | Mut |
| TCGA-B0-4818 | Mut |
| TCGA-CZ-4861 | Mut |
| TCGA-CZ-5455 | Mut |
| TCGA-B0-4833 | Mut |
| TCGA-A3-3380 | Mut |
| TCGA-B2-5635 | Mut |
| TCGA-AK-3444 | Mut |
| TCGA-BP-4992 | Mut |
| TCGA-BP-4976 | Mut |
| TCGA-AK-3450 | Mut |
| TCGA-CZ-5457 | Mut |
| TCGA-B0-4824 | Mut |
| TCGA-B0-5402 | Mut |
| TCGA-A3-3324 | Mut |
| TCGA-CJ-6032 | Mut |
| TCGA-CJ-6032 | Mut |
| TCGA-B0-5691 | Mut |
| TCGA-B8-5159 | Mut |
| TCGA-BP-5183 | Mut |
| TCGA-B8-5164 | Mut |
| TCGA-BP-5190 | Mut |
| TCGA-CW-5583 | Mut |
| TCGA-CZ-5985 | Mut |
| TCGA-B0-5104 | Mut |
| TCGA-B2-3924 | Mut |
| TCGA-B0-5699 | Mut |
| TCGA-B0-5699 | Mut |
| TCGA-BP-4162 | Mut |
| TCGA-BP-4162 | Mut |
| TCGA-A3-3322 | Mut |
| TCGA-B0-4814 | Mut |
| TCGA-CJ-5675 | Mut |
| TCGA-CZ-5986 | Mut |
| TCGA-B0-5705 | Mut |
| TCGA-B2-4102 | Mut |
| TCGA-B2-4102 | Mut |
| TCGA-AK-3458 | Mut |
| TCGA-B0-4828 | Mut |
| TCGA-BP-4995 | Mut |
| TCGA-BP-4340 | Mut |
| TCGA-B8-4148 | Mut |
| TCGA-CZ-4853 | Mut |
| TCGA-B0-5075 | Mut |
| TCGA-A3-3385 | Mut |
| TCGA-AK-3455 | Mut |
| TCGA-BP-4164 | Mut |
| TCGA-BP-4164 | Mut |
| TCGA-BP-4164 | Mut |
| TCGA-BP-4164 | Mut |
| TCGA-BP-4164 | Mut |
| TCGA-BP-4164 | Mut |
| TCGA-CZ-5989 | Mut |

The information of *VHL* mutations was extracted from raw MAF files at the Broad GDAC FireHose (gdac.broadinstitute.org)
