# Supplementary material for: SERPINH1 overexpression in clear cell renal cell carcinoma: association with poor clinical outcome and its potential as a novel prognostic marker
Source: J Cell Mol Med. 2017 Dec 14;22(2):1224–35. doi: 10.1111/jcmm.13495 (PMC5783852; doi:10.1111/jcmm.13495)
Supplement: Supplementary file 17 [file JCMM-22-1224-s017.docx]

**Supplementary materials and methods**

**Gene Set Enrichment Analysis**

The association between phenotypes, gene set expression level and expression levels of specific genes was analyzed using Gene Set Enrichment Analysis (GSEA v2.2, http://www.broad.mit.edu/gsea) as previously reported (Zheng *et al*, 2017). A gene set is considered significantly enriched when the false discovery rate (FDR) score is < 0.25.

**Cell culture**

Human ccRCC ACHN, 786-O cell line and human embryonic kidney 293 cell (HEK293, all from American Type Culture Collection, ATCC, Manassas, VA) were grown in RPMI 1640 medium and Dulbecco's modified Eagle's medium (DMEM) (both from Gibco, Waltham, MA), respectively. These media contain 10% fetal bovine serum (FBS, Hyclone, Logan, UT) and 1% antibiotic-antimycotic agent (Life Technologies, Inc., Grand Island, NY). Cells were grown at 37°C and 5% CO_2_.

**siRNA-mediated transient SERPINH1 knockdown**

Small interfering RNA (siRNA) duplexes directed against SERPINH1 (sc-35600) and control scrambled RNAi (sc-37007) were synthesized by Santa Cruz (Dallas, TX). Cells were grown to 80% confluency in 35-mm dishes, transfected with 2µl Lipofectamine 2000 (Invitrogen, Carlsbad, CA), and mixed with 50pmol of the synthetic SERPINH1 siRNA pool. The cells were then harvested and analyzed after 48h of transfection.

**Western blotting**

The primary antibodies specific for E-Cadherin, Vimentin, Slug and Snail were all bought from Cell Signaling Technology (Beverly, MA).

**The Human Protein Atlas**

The IHC-based protein expression data including high-resolution images were viewed and downloaded from the Human Protein Atlas (THPA) web portal (www.proteinatlas.org).

**Statistical analysis**

ROC curve and AUC analyses were applied to discriminate normal and tumor tissues. The correlation analysis of genes expression level was demonstrated by Pearson correlation test.

**Supplementary figure legends**

Supplementary Figure 1

The levels of 35 differentially expressed genes are significantly correlated with the level of TGFβ in ccRCC. The mRNA levels were downloaded from TCGA_KIRC dataset. False discovery rate (FDR) gives the estimated probability that a gene set with a given normalized ES (NES) represents a false-positive finding; FDR < 0.25 is a widely accepted cutoff for the identification of biologically significant gene sets.

Supplementary Figure 2

SERPINH1 is correlated with poor prognosis of all stage ccRCC patients. **(A)** Left: Venn diagrams showing the overlap between genes which predict meaningful risk ratio of DFS (univariate cox analysis, low and high level was divided according to median value; blue) and genes associated with DFS prognosis (two-tailed *t* test; good prognosis*/*poor prognosis; pink) from the TCGA_KIRC dataset. Upregulated genes (UP, low/high level group) with HR < 1, *P* < 0.01 and downregulated (DN) genes with HR > 1, *P* < 0.01 among 35 genes were exhibited in blue color. Upregulated genes with Fold change (FC, good prognosis/poor prognosis group) < 0.8, *P* < 0.05 and downregulated genes with FC > 1.25, *P* < 0.05 among 35 genes were exhibited in pink color. The gene names shown below are the genes shared by blue and pink groups for DFS, respectively. Right: Enrichment plots showed that the expression of *SERPINH1* got the second ranking metric score among 35 dysregulated genes enriched in poor prognosis group according to DFS prognosis status (good or poor) of samples from TCGA_KIRC dataset. **(B)** The expression level of *SERPINH1* was increased in patients with poor prognosis for OS and DFS. ****P* < 0.001**. (C)** Enrichment plots showed that the expression level of *SERPINH1* got the highest ranking metric score among 35 dysregulated genes enriched in poor prognosis group according to OS and DFS prognosis status (good or poor) of samples from TCGA_KIRC (RNA Seq v2) early stage (Stage I and II) and advanced stage (Stage III and IV) dataset, respectively.

Supplementary Figure 3

*SERPINH1* is positively correlated with TGFβ signaling in EMT and the level of EMT gene set/EMT markers in ccRCC. **(A)** Pearson correlation analysis results revealed *SERPINH1* expression level was significantly correlated with TGFβ signaling in EMT of ccRCC tissues. **(B)** GSEA results revealed that high *SERPINH1* level was positively correlated with EMT phenotype (FDR < 0.25). **(C)** The expression level of *SERPINH1* was negatively correlated with the level of epithelial cell marker (E-Cadherin) and positively correlated with the level of mesenchymal cell markers (Snail, Vimentin and Slug).

Supplementary Figure 4

SERPINH1 regulates the expression of EMT markers in ccRCC and HEK293 cell lines. Knockdown of SERPINH1 reversed the expression of EMT markers in ccRCC and normal kidney cell lines. The expression level of epithelial cell marker (E-Cadherin) was increased in SERPINH1 knockdown cells. Meantime, the expression level of mesenchymal cell markers (Snail, Vimentin and Slug) was decreased in SERPINH1 knockdown cells.

Supplementary Figure 5

SERPINH1 expression level can discriminate normal and tumor tissues and its expression level is abnormally upregulated in ccRCC tissues as T stage progresses. **(A)** ROC curve for evaluating the effectiveness of *SERPINH1* mRNA expression level in discriminating normal and ccRCC tissues. The area under curve(AUC) was 0.9487 (95% CI: 0.9280-0.9649). **(B)** *SERPINH1* expression level increased in all four TNM stages of ccRCC tissues. ****P* < 0.001**. (C)** The Human Protein Atlas database results showed that SERPINH1 protein level was abnormally upregulated in ccRCC tissues (*n* = 12) compared with adjacent normal kidney tissues (*n* = 3). ***P* < 0.01. **(D)** SERPINH1 expression level increased as T stage progressed and failed to discriminate between ccRCC patients with and without lymph node metastasis. ******P* < 0.001, NS: not significant.

Supplementary Figure 6

SERPINH1 is correlated with poor clinical outcome of early stage ccRCC patients. **(A)** High *SERPINH1* mRNA level predicted a shorter OS and DFS time in stage I ccRCC patients. **(B)** High SERPINH1 protein level predicted a shorter OS time in stage I ccRCC patients. *P* values were calculated with a log-rank test.

Supplementary Figure 7

External comparison with reported prognostic markers-1. mRNA levels of previously reported prognostic markers were extracted from TCGA_KIRC dataset. **(A)** *HADHA* and *DIABLO* mRNA levels are not correlated with poor clinical outcome of ccRCC patients. *HADHA* and *DIABLO* mRNA levels between patients with and without recurrence or metastasis were compared by independent sample *t*-test. Outlier values (> Mean ± 3 SD) were removed. **P* < 0.05, ***P* < 0.01, NS: not significant. **(B)** *SERPINH1* expression level presents the stronger DFS prognosis predicting ability than *PDZK1*. ROC curve for *PDZK1* and *SERPINH1* mRNA levels in classifying DFS of ccRCC patients. The area under curve (AUC) and the corresponding 95% CI are shown in the plots.

Supplementary Figure 8

External comparison with reported prognostic markers-2. mRNA levels were extracted from TCGA_KIRC dataset. *LDHA*, *BIRC*, *CA9*, *FSCN2* and *IMP3* mRNA levels between patients with and without metastasis were compared by independent sample *t*-test. Outlier values (> Mean ± 3 SD) were removed. ****P* < 0.01, NS: not significant.

Supplementary Figure 9

SERPINH1 level shows no difference between *VHL*-WT and *VHL*-MT patients and predicts the OS prognosis of *VHL*-WT ccRCC patients. **(A)** *SERPINH1* level showed no difference between *VHL*-WT and *VHL*-MT patients. NS: not significant. **(B)** *SERPINH1* mRNA level presented superior capability in predicting the OS prognosis of *VHL*-WT ccRCC patients to *VHL*-MT patients. **(C)** *PDZK1* mRNA level could also better predict the OS prognosis of *VHL*-WT patients than *VHL*-MT patients, *P* values were calculated with a log-rank test.

Supplementary Figure 10

SERPINH1 is an independent DFS prognostic marker in *VHL*-WT ccRCC patients. SERPINH1 was an independent prognostic factor for DFS in ccRCC. Univariate (OS-U) and multivariate (OS-M) cox regression models in ccRCC. ****P* < 0.001.
